# Modelling the global burden of drug-resistant tuberculosis avertable by a post-exposure vaccine

**DOI:** 10.1038/s41467-020-20731-x

**Published:** 2021-01-18

**Authors:** Han Fu, Joseph A. Lewnard, Isabel Frost, Ramanan Laxminarayan, Nimalan Arinaminpathy

**Affiliations:** 1 MRC Centre for Global Infectious Disease Analysis; and the Abdul Latif Jameel Institute for Disease and Emergency Analytics (J-IDEA), School of Public Health, Imperial College London, London, W2 1PG UK; 2 Division of Epidemiology, School of Public Health, University of California, Berkeley, Berkeley, CA 94720 USA; 3 Center for Disease Dynamics, Economics & Policy, New Delhi, India; 4 Department of Infectious Disease, Imperial College London, London, W2 1NY UK; 5 Princeton University, Princeton, NJ 08544 USA

**Keywords:** Computational models, Vaccines, Tuberculosis, Epidemiology

## Abstract

There have been notable advances in the development of vaccines against active tuberculosis (TB) disease for adults and adolescents. Using mathematical models, we seek to estimate the potential impact of a post-exposure TB vaccine, having 50% efficacy in reducing active disease, on global rifampicin-resistant (RR-) TB burden. In 30 countries that together accounted for 90% of global RR-TB incidence in 2018, a future TB vaccine could avert 10% (95% credible interval: 9.7–11%) of RR-TB cases and 7.3% (6.6–8.1%) of deaths over 2020–2035, with India, China, Indonesia, Pakistan, and the Russian Federation having the greatest contribution. This impact would increase to 14% (12–16%) and 31% (29–33%) respectively, when combined with improvements in RR-TB diagnosis and treatment relative to a scenario of no vaccine and no such improvements. A future TB vaccine could have important implications for the global control of RR-TB, especially if implemented alongside enhancements in management of drug resistance.

## Abstract (web summary)

Vaccines preventing tuberculosis disease progression have shown promising results in recent trials. Here, the authors use mathematical modelling to estimate that this type of vaccine could avert 10% of cases of rifampicin-resistant tuberculosis and 7% of deaths from 2020-2035.

## Introduction

The emergence of drug resistance poses a pressing challenge in the control of tuberculosis (TB) worldwide^1^. In 2018, an estimated 500,000 incident TB cases worldwide (out of a total of 10 million incident cases) were resistant to rifampicin, one of the key drugs in first-line anti-TB treatment^2^. Second-line treatment for these cases is costly, protracted and toxic, with an average treatment success of only 56%, compared to 85% for drug-sensitive TB^2^. Recent years have seen important advances in the development of shorter and safer regimens for treatment of rifampicin-resistant (RR-) TB^3–5^, as well as the potential for regimens to avoid using ineffective drugs by early detection of broader resistance profiles^6,7^; nonetheless, global uptake of these new regimens remains slow^3^.

Prevention of drug-resistant TB may be as important as improving patient outcomes for achieving desired reductions in the burden of disease^8^. Although recent years have seen notable advances in the development of simplified preventive regimens against drug-sensitive TB^9,10^, preventive therapy against drug-resistant infection is complicated by the fact that there is currently no assay for latent TB infection (LTBI) that can determine drug susceptibility of the infecting strain. In recent contacts of multidrug-resistant TB, preventive treatment can be tailored to the drug sensitivity status of the index case, but this approach does not address those with immunity to TB-specific antigens and no known contact. However, recent developments have raised the prospect of prevention through vaccination^11^. Informed in part by seminal modelling analysis^12^, in 2018 the World Health Organization (WHO) defined a preferred product profile for TB vaccine^13^. Amongst priorities for vaccine development, this document identified the ability to protect adults and adolescents with LTBI from developing the active form of the disease, with at least 50% efficacy, and offering at least ten years of protection^13^. In subsequent results from a phase 2b trial, amongst adults and adolescents with LTBI in Kenya and South Africa, the M72/AS01_E_ (hereafter M72) vaccine decreased TB incidence by almost half^14^.

By offering protection regardless of drug sensitivity status, future TB vaccines may offer an important opportunity to reduce the transmission of drug-resistant TB, as well as the overall burden of TB. Prevention of TB through vaccination may have the corollary benefit of reducing population-level selection of resistant strains, as the use of anti-TB drugs declines. However, the potential impact of a future TB vaccine on the global burden of drug-resistant TB remains unknown. It is also uncertain how the impact of such a vaccine may compare with the uptake of new second-line regimens, that are shorter, safer and potentially more effective than earlier regimens involving injectable drugs^5^.

In the present work, we seek to address these questions using mathematical modelling of TB transmission dynamics, in 30 countries with a high burden of drug-resistant TB (Table 1). We modelled the potential impact of a rollout of an M72-like, ‘post-exposure’ vaccine (i.e. one intended for use amongst those already infected with TB) amongst adults and adolescents, and assessed health impacts between 2020 and 2035, with a focus on RR-TB. As a secondary analysis, we examined the potential contribution of a TB vaccine, to antimicrobial resistance more broadly than TB. There is evidence indicating that TB cases are often treated with empiric antibiotic therapy in primary care settings^15^, including the use of quinolones^16,17^. Therefore, if a vaccine reduces overall TB burden, it may also reduce the prevalence of symptoms that would trigger antibiotic consumption, reducing selection for resistance not only in TB but other pathogens or commensal bacteria that might be exposed^18^. We estimated the potential impact of a future TB vaccine, on reducing antibiotic consumption in this way.

**Table 1:** List of the 30 countries selected for the modelling analysis.

Countries having a strong private healthcare sector (i)	Countries where HIV is a driver of TB epidemiology (ii)	Countries with both a private sector and driving role of HIV in TB	All remaining countries
• India (IND) • Pakistan (PAK) • Indonesia (IDN) • Philippines (PHL) • Myanmar (MMR) • Bangladesh (BGD) • Ethiopia (ETH)	• Russia Federation (RUS) • Ukraine (UKR) • South Africa (ZAF) • Mozambique (MOZ) • DR Congo (COD) • Zimbabwe (ZWE)	• Nigeria (NGA) • Thailand (THA) • Angola (AGO) • Kenya (KEN)	• China (CHN) • Viet Nam (VNM) • DPR Korea (PRK) • Kazakhstan (KAZ) • Uzbekistan (UZB) • Somalia (SOM) • Peru (PER) • Kyrgyzstan (KGZ) • Papua New Guinea (PNG) • Tajikistan (TJK) • Belarus (BLR) • Republic of Moldova (MDA) • Azerbaijan (AZE)

These are the countries with the largest absolute burden of RR-TB, and that accounted in 2018 for 90% of global RR-TB incidence (Supplementary Fig. 16). The table shows countries grouped into four different categories, for the purpose of modelling. The ISO alpha-3 code for each country is also shown in brackets. We developed a specific model structure for each of the country categories, and calibrated that model separately for each country within each category. Footnotes: (i) We drew these countries from the World Health Organization list of priority countries for public-private-mix programmes^46^, namely those countries where the private sector plays a strong role in managing TB patients, and which account for the bulk of unreported cases globally. (ii) We identified these countries as those in which HIV coinfection accounted for at least 10% of estimated TB incidence in 2018.

*HIV* human immunodeficiency virus, *RR-TB* rifampicin-resistant tuberculosis.

## Results

### Overview

**Fig. 1:**
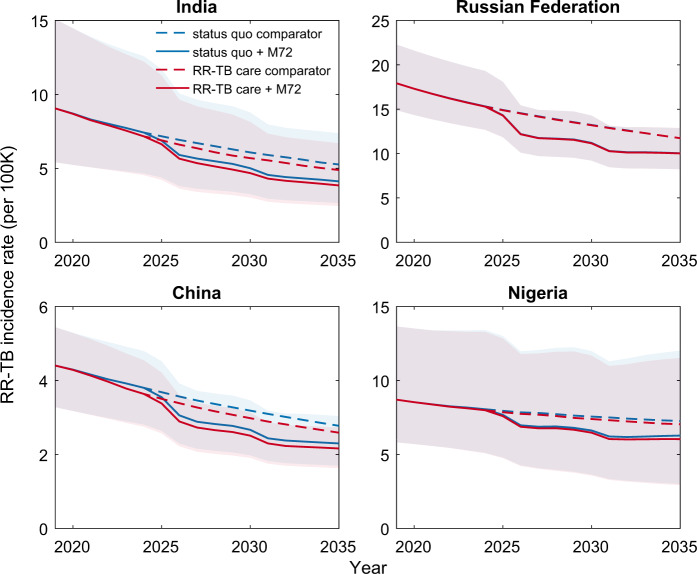
Illustration of the projected impact on RR-TB of an M72-like vaccine. *n* = 200 posterior samples. Median (solid and dashed lines) and 95% Bayesian credible intervals (CrIs) of rifampicin-resistant tuberculosis (RR-TB) incidence rates over 2019–2035 are presented. For clarity, uncertainty regions with CrIs are only shown for the vaccine (not comparator) scenarios. These projections correspond to a post-exposure vaccine that reduces the risk of reactivation of latent TB infection by 50%, and that is implemented through routine vaccination and catch-up campaign amongst those over 15-years old. The figure shows projections for the countries with the highest absolute burden of RR-TB from each of the country categories listed in Table 1. Blue dashed curves illustrate a ‘status quo’ baseline, assuming no vaccine implementation and no change in the management of RR-TB. Blue solid curves show the impact of vaccination. Red curves show corresponding dynamics, under an alternative ‘improved RR-TB management’ baseline where the detection of RR-TB at the point of TB diagnosis is increased to 85%, and second-line treatment success is increased to 75%, in countries that have not yet achieved these targets. The Russian Federation in particular shows a lower impact of improved RR-TB management than other countries, owing to its already-higher coverage of drug susceptibility tests (Supplementary Fig. 10). Although the 95% CrIs for these projected dynamics with vaccination overlap in these regions, the overall incidence reductions are significant, in the sense of having uncertainty intervals that are strictly positive (Table 2, Supplementary Fig. 9).

Supplementary Figs. 1–6 and Supplementary Table 1 show the results of model calibration in the 30 countries accounting for 90% of RR-TB incidence in 2018, based on four different model structures that capture variations in the epidemiology of TB and health systems across these countries (Table 1). Additional results show model validation with data that was not used for calibration, including prevalence surveys where available (Supplementary Fig. 7) and model-based estimates for the proportion of incident, drug-resistant TB that arises through transmission, rather than through treatment (Supplementary Fig. 8). Figure 1 shows illustrative results for RR-TB incidence trajectories in India, the Russian Federation, China, and Nigeria, as example countries representing each of the model structures (Table 1); Supplementary Fig. 9 shows these same dynamics, but illustrating the cases averted over time.

### Vaccine impact on RR-TB incidence

**Fig. 2:**
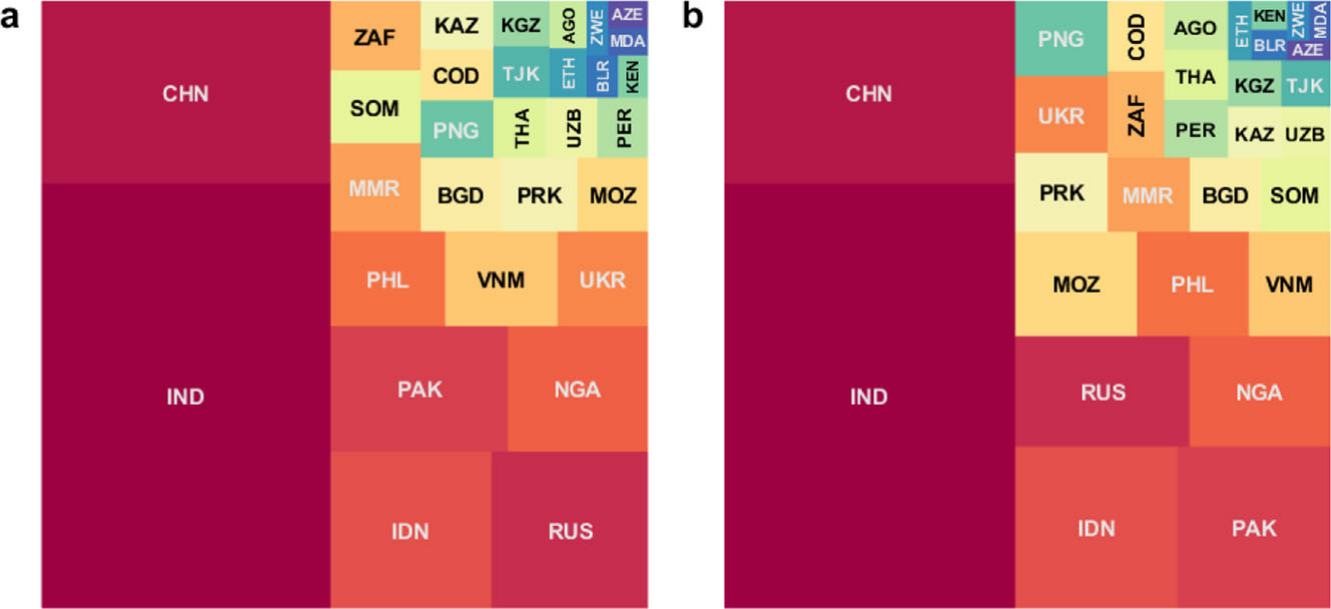
Country-wise contribution to vaccine-avertable incidence of RR-TB. The area of each square is proportional to the median estimate from *n* = 200 posterior samples, for the absolute number of avertable rifampicin-resistant tuberculosis (RR-TB) cases by a post-exposure, M72-like vaccine, between 2020 and 2035. Panel **a** shows cases averted by a vaccine alone, while panel **b** shows cases averted by a combination of a vaccine and measures to improve the management of RR-TB (both relative to a scenario of no vaccine, and no improvement in RR-TB, i.e. status quo). Countries are denoted by their ISO alpha-3 codes and their corresponding full names are listed in Table 1. Colours for each country are only for the purpose of display, and do not designate any quantitative scale.

Figure 2 summarises the overall impact in terms of cumulative RR-TB cases averted between 2020 and 2035, illustrating dominant contributions from India and China, as well as from high-burden countries including Indonesia, Pakistan, the Russian Federation and Nigeria. Globally, relative to a status quo comparator, an M72-like vaccine would avert 620,000 cases (95% credible intervals [CrI]: 516,000–867,000) of RR-TB between 2020 and 2035 (Table 2). Molecular diagnostic tools are offering new opportunities for early recognition of rifampicin resistance^19^, while new, shorter, safer second-line regimens may facilitate improved treatment outcomes^5^. If use of an M72-like vaccine is combined with increased uptake of these tools (i.e. improved management of RR-TB), the overall impact would increase to 831,000 RR-TB cases (95% CrI: 643,000–1,170,000) averted.

**Table 2:** Summary of model-projected impact of a post-exposure, M72-like vaccine on RR-TB burden.

Country/Region	M72-like vaccine				M72-like vaccine and improved RR-TB management			
	Averted RR-TB cases		Averted RR-TB deaths		Averted RR-TB cases		Averted RR-TB deaths	
	Number, thousands	Proportion, %	Number, thousands	Proportion, %	Number, thousands	Proportion, %	Number, thousands	Proportion, %
India	201(116, 428)	11(9.6, 12)	47(26, 99)	7.3(5.8, 9.1)	263(141, 591)	14(11, 18)	232(132, 453)	36(33, 39)
Indonesia	41(29, 63)	12(11, 13)	8.1(5.9, 12)	8.1(7.2, 8.9)	56(38, 93)	16(13, 22)	42(30, 61)	41(39, 44)
Myanmar	13(7.5, 21)	9.5(7.7, 11)	2.6(1.5, 4.5)	7.5(5.9, 9.0)	13(7.5, 21)	9.5(7.7, 11)	2.6(1.5, 4.5)	7.5(5.9, 9.0)
Bangladesh	9.8(4.8, 18)	11(10, 12)	1.4(0.67, 2.6)	8.4(7.1, 9.2)	11(5.4, 21)	13(11, 17)	2.1(0.97, 4.4)	13(10, 16)
DPR Korea	9.1(3.9, 31)	14(11, 18)	1.0(0.40, 3.7)	10(8.2, 14)	16(4.6, 66)	23(12, 39)	2.6(0.72, 10)	25(14, 39)
Thailand	5.2(1.9, 9.2)	10(8.5, 11)	1.1(0.40, 1.9)	7.8(6.4, 8.7)	6.8(2.0, 15)	13(9.3, 18)	3.7(1.4, 6.9)	26(23, 30)
**South-East Asia Region**	**286(197, 525)**	**11(10, 12)**	**62(42, 116)**	**7.5(6.3, 8.8)**	**375(251, 712)**	**14(12, 17)**	**286(190, 513)**	**35(32, 37)**
China	86(55, 133)	9.7(8.8, 12)	8.2(5.3, 12)	7.2(6.6, 9.0)	113(62, 274)	13(9.9, 24)	39(25, 58)	34(32, 41)
Philippines	18(8.6, 36)	11(8.4, 12)	2.0(0.97, 4.1)	6.4(5.1, 8.0)	25(12, 62)	15(10, 23)	11(6.6, 23)	39(36, 41)
Viet Nam	18(7.0, 43)	13(9.8, 17)	2.2(0.88, 5.6)	10(6.8, 13)	18(7.2, 45)	14(10, 18)	4.5(2.1, 9.7)	20(16, 23)
Papua New Guinea	6.8(2.9, 16)	15(12, 19)	0.98(0.41, 2.3)	12(8.8, 16)	15(5.6, 40)	33(25, 47)	2.2(0.82, 5.8)	27(19, 40)
**West Pacific Region**	**134(89, 186)**	**11(9.6, 12)**	**14(9.6, 19)**	**7.8(7.0, 9.3)**	**181(113, 338)**	**14(11, 23)**	**60(41, 81)**	**33(31, 38)**
Russian Federation	40(30, 53)	9.4(8.9, 9.8)	7.8(5.9, 10)	7.3(6.9, 7.7)	41(31, 55)	9.5(8.8, 11)	40(30, 52)	37(35, 38)
Ukraine	14(9.2, 20)	9.5(9.0, 9.9)	3.0(2.1, 4.2)	7.3(6.7, 7.6)	15(10, 21)	10(9.2, 12)	20(13, 27)	48(45, 49)
Kazakhstan	5.8(3.5, 8.7)	11(9.9, 13)	0.55(0.34, 0.87)	8.8(7.9, 10)	5.8(3.5, 8.7)	11(9.9, 13)	0.55(0.34, 0.87)	8.8(7.9, 10)
Uzbekistan	5.1(3.1, 7.6)	9.5(8.4, 11)	0.94(0.61, 1.4)	7.4(6.3, 8.6)	5.3(3.4, 7.9)	10(8.7, 12)	4.2(2.9, 6.2)	33(30, 35)
Kyrgyzstan	4.2(3.1, 5.5)	12(11, 13)	0.61(0.46, 0.79)	9.4(8.8, 9.8)	5.3(3.7, 6.9)	15(11, 17)	3.2(2.5, 4.2)	50(49, 51)
Tajikistan	4.7(2.4, 9.1)	16(13, 18)	0.78(0.41, 1.5)	12(9.2, 13)	4.8(2.3, 9.2)	16(13, 18)	2.2(1.3, 3.7)	31(29, 33)
Belarus	2.2(1.3, 3.4)	11(9.5, 12)	0.24(0.16, 0.36)	8.3(7.3, 9.8)	2.3(1.4, 3.6)	11(9.7, 13)	0.68(0.43, 1.0)	23(21, 26)
Republic of Moldova	1.8(1.3, 2.3)	10(9.5, 11)	0.30(0.22, 0.39)	7.8(7.3, 8.4)	1.8(1.3, 2.5)	10(9.3, 12)	1.8(1.4, 2.3)	47(46, 48)
Azerbaijan	1.7(1.1, 2.8)	10(9.2, 13)	0.30(0.19, 0.47)	7.7(6.7, 9.9)	1.8(1.1, 3.3)	11(9.1, 15)	1.3(0.90, 2.0)	36(32, 38)
**European Region**	**81(68, 92)**	**10(9.6, 10)**	**15(12, 17)**	**7.6(7.3, 7.9)**	**84(72, 96)**	**10(9.9, 11)**	**74(61, 86)**	**38(37, 39)**
Pakistan	36(22, 60)	9.4(8.5, 10)	6.2(4.0, 10)	6.3(5.4, 7.0)	53(31, 93)	14(11, 17)	24(15, 39)	24(22, 25)
Somalia	11(3.0, 29)	9.6(7.1, 13)	2.1(0.60, 5.9)	7.7(5.2, 11)	11(3.1, 29)	10(7.3, 13)	2.2(0.63, 6.1)	8.2(5.4, 11)
**Eastern Mediterranean Region**	**47(30, 79)**	**9.5(8.4, 11)**	**8.5(5.5, 14)**	**6.7(5.6, 7.7)**	**66(42, 108)**	**13(11, 15)**	**26(17, 42)**	**21(17, 23)**
Nigeria	29(14, 55)	8.5(6.7, 10)	8.3(4, 17)	7.1(5.5, 8.4)	33(16, 65)	10(7.9, 11)	10(4.9, 21)	8.8(7.0, 10)
South Africa	10(6.0, 19)	8.3(7.5, 9.2)	2.9(1.6, 5.7)	6.7(5.5, 7.8)	10(5.9, 21)	8.6(7.3, 11)	14(8.2, 23)	31(27, 33)
Mozambique	8.5(4.5, 16)	5.2(3.5, 6.6)	3.2(1.6, 5.9)	5.2(3.7, 6.3)	27(9.8, 100)	16(10, 33)	15(6.2, 42)	24(16, 38)
DR Congo	6.1(3.1, 13)	7.5(6.1, 9.3)	0.81(0.40, 2.2)	5.6(4.5, 7.1)	8.5(3.9, 25)	11(7.6, 16)	1.7(0.68, 5.9)	12(9.0, 17)
Angola	2.9(1.1, 9.5)	7.7(5.3, 9.9)	0.31(0.09, 1.6)	6.1(3.9, 7.7)	6.7(2.0, 33)	19(10, 29)	1.7(0.70, 7.8)	35(29, 40)
Kenya	2.1(1.1, 4.4)	8.0(6.2, 9.9)	0.46(0.22, 1.2)	6.3(4.6, 7.9)	2.2(1.1, 5.0)	8.3(6.2, 11)	0.90(0.46, 2.0)	12(11, 13)
Ethiopia	3.0(1.8, 5.0)	10(8.7, 11)	0.39(0.21, 0.69)	7.6(6.2, 8.5)	3.0(1.8, 5.0)	10(8.7, 11)	0.40(0.22, 0.71)	7.7(6.3, 8.7)
Zimbabwe	1.9(1.2, 3.0)	9.8(8.8, 11)	0.61(0.38, 0.94)	7.9(7.1, 8.8)	1.8(1.1, 2.8)	9.3(8.3, 10)	2.6(1.7, 3.7)	33(31, 35)
**African Region**	**66(47, 97)**	**7.7(6.5, 8.8)**	**18(12, 28)**	**6.5(5.6, 7.4)**	**101(66, 170)**	**12(9.8, 17)**	**48(33, 78)**	**18(14, 24)**
Peru/**Region of America**	**5.0(3.3, 8.2)**	**12(11, 14)**	**0.45(0.31, 0.75)**	**9.0(8.3, 11)**	**7.8(4.7, 13)**	**18(15, 24)**	**2.0(1.3, 3.0)**	**39(36, 43)**
**Total**	**620(516, 867)**	**10(9.7, 11)**	**119(96, 174)**	**7.3(6.6, 8.1)**	**831(643, 1170)**	**14(12, 16)**	**499(391, 729)**	**31(29, 33)**

Estimates show median and (in brackets) 95% Bayesian credible intervals, both estimated from *n* = 200 posterior samples, all relative to a ‘status quo’ scenario. Estimates at regional level and global level are in bold. We show averted RR-TB cases and deaths by an M72-like vaccine alone and in combination with improved RR-TB management. The vaccine is assumed to reduce the risk of reactivation by 50%, and to be deployed amongst adults and adolescents with latent TB infection in the population.

*RR-TB* rifampicin-resistant tuberculosis.

**Fig. 3:**
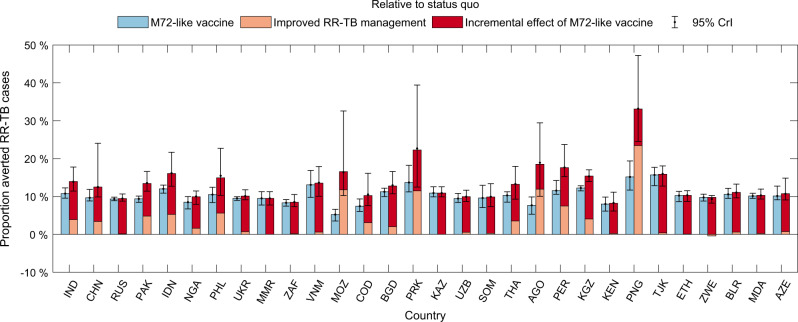
Projected percent cases of RR-TB averted by an M72-like vaccine. Rectangular bars show median estimates and error bars show 95% Bayesian credible intervals (CrIs), both estimated from *n* = 200 posterior samples for each country. Blue bars show the vaccine-avertable proportions of rifampicin-resistant tuberculosis (RR-TB) cases relative to a ‘status quo’ scenario, while adjacent bars together show averted proportions in combination with ‘improved RR-TB management’, as outlined in the caption to Fig. 1. The latter bars are stratified to show: (i) the impact of improved management of RR-TB alone, i.e. in the absence of vaccination (orange segment), and (ii) the incremental impact of vaccination, acting in combination with these improvements (red segment). Error bars on the stacked orange and red bars show the 95% CrIs of the total impact of a vaccine combined with improved management of RR-TB. Countries are ranked in a descending order according to the number of RR-TB incident cases in 2018.

Figure 3 shows percent reductions of RR-TB cases by country. Globally, the effect of vaccination would be to avert 10% (95% CrI: 9.7–11%) of RR-TB cases between 2020 and 2035, relative to a status quo comparator (Table 2). Combined with improved RR-TB management, this impact would increase to 14% (95% CrI: 12–16%) of RR-TB cases. For countries having only small improvement gaps in coverage of drug susceptibility testing and success of second-line treatment, like Myanmar and Kazakhstan (Supplementary Fig. 10), improved management of RR-TB would have only limited incremental impact on RR-TB burden, in addition to vaccination. Supplementary Fig. 11 illustrates that, at the global level, ~2500 adolescents and adults with LTBI would need to be vaccinated to avert 1 RR-TB case.

### Vaccine impact on RR-TB mortality

**Fig. 4:**
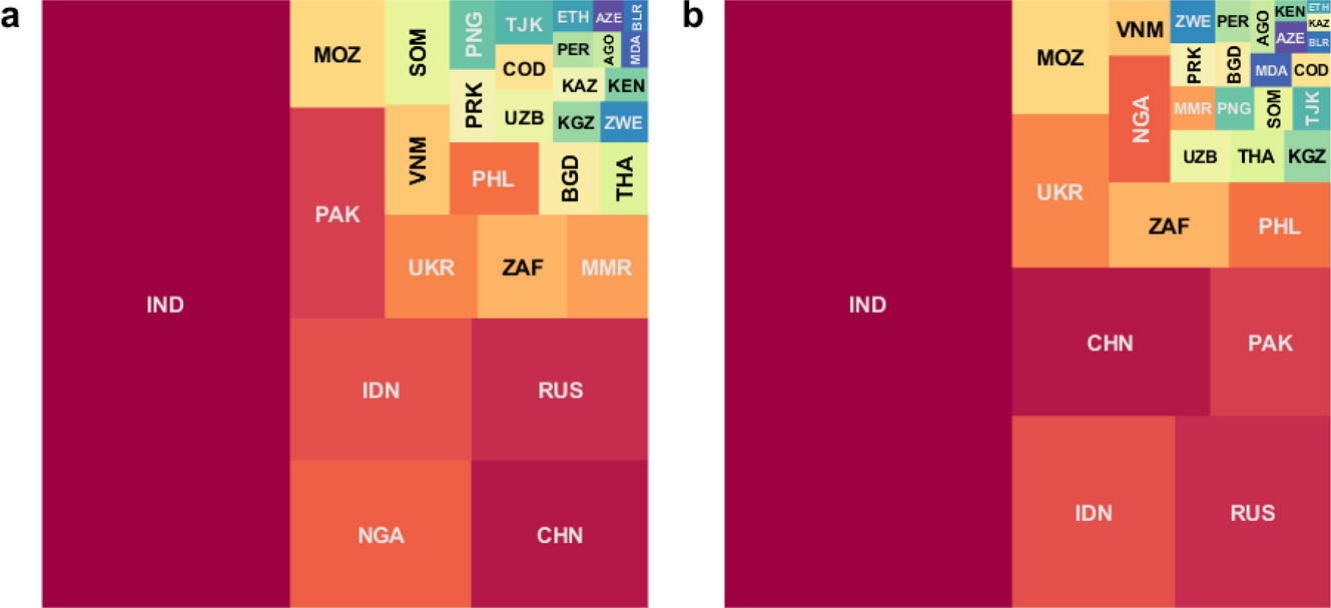
Country-wise contribution to vaccine-avertable deaths from RR-TB. As for Fig. 2, the area of each square here is proportional to the median estimate from *n* = 200 posterior samples, for the absolute number of avertable rifampicin-resistant tuberculosis (RR-TB) deaths by a post-exposure, M72-like vaccine, between 2020 and 2035. Panel **a** shows deaths averted by a vaccine alone, while panel **b** shows deaths averted by a combination of a vaccine and measures to improve the management of RR-TB (both relative to a scenario of no vaccine, and no improvement in RR-TB, i.e. status quo). Countries are denoted by their ISO alpha-3 codes and their corresponding full names are listed in Table 1. Colours for each country are only for the purpose of display, and do not designate any quantitative scale.

**Fig. 5:**
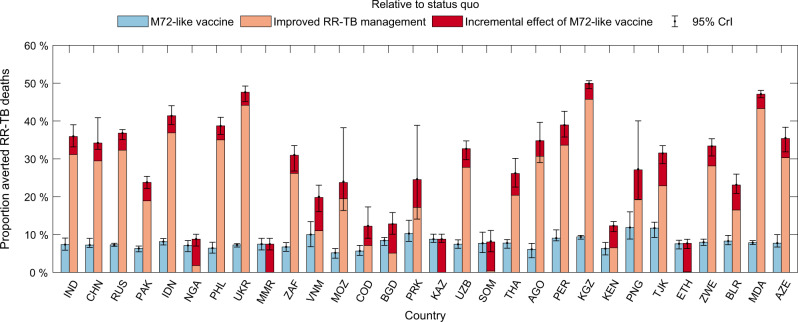
Projected percent deaths from RR-TB averted, as a result of anti-TB vaccination. Rectangular bars show median estimates and error bars show 95% Bayesian credible intervals (CrIs), both estimated from *n* = 200 posterior samples for each country. As in Fig. 3, blue bars here show the vaccine-avertable proportions of rifampicin-resistant tuberculosis (RR-TB) deaths relative to a ‘status quo’ scenario, while adjacent bars show averted proportions in combination with ‘improved RR-TB management’, as outlined in the caption to Fig. 1. The latter bars are stratified to show: (i) the impact of improved management of RR-TB alone, i.e. in the absence of vaccination (orange segment), and (ii) the incremental impact of vaccination, acting in combination with these improvements (red segment). Error bars on the stacked orange and red bars show the 95% CrIs of the total impact of a vaccine combined with improved management of RR-TB. Countries are ranked in a descending order according to the number of RR-TB incident cases in 2018.

Figures 4 and 5 show corresponding results in terms of RR-TB mortality: globally, vaccination would avert 119,000 (95% CrI: 96,000–174,000) RR-TB deaths between 2020 and 2035, amounting to a reduction of 7.3% (95% CrI: 6.6–8.1%). Improvement in RR-TB management showed a greater impact on preventing mortality than incidence of RR-TB burden. When combined with vaccination, 499,000 (95% CrI: 391,000–729,000) RR-TB deaths would be averted between 2020 and 2035, amounting to a reduction of 31% (95% CrI: 29–33%) (Table 2). Supplementary Fig. 11 illustrates that, at the global level, ~13,000 adolescents and adults with LTBI would need to be vaccinated to avert 1 death from RR-TB. This estimate may vary by local settings, depending on country-specific rates of TB mortality.

In all of these estimates we assumed the vaccine would have the same efficacy amongst individuals with and without human immunodeficiency virus (HIV) infection. Supplementary Table 2 shows sensitivity analyses when assuming that vaccine efficacy is reduced by half amongst those with HIV. Such a scenario shows only a modest effect on the global impact of vaccination on RR-TB: for example, with 10% (95% CrI: 9.7–11%) cases averted under the status quo scenario, being reduced to 10% (95% CrI: 9.5–11%).

### Vaccine impact on antibiotic consumption

Supplementary Fig. 12 and Supplementary Table 3 additionally illustrate how vaccination reduces the total amount of second-line TB treatment needed, because of its impact on RR-TB. By contrast, efforts to improve the management of RR-TB would increase the demand for second-line drugs, as a result of increased identification of resistant cases (Supplementary Table 4). Finally, Supplementary Fig. 13 illustrates that future TB vaccines are likely to have only modest impact on antibiotic consumption beyond TB drugs, typically amounting to <1% of total estimated antibiotic consumption in each country. This limited impact arises because of the relative rarity of TB amongst those with respiratory symptoms receiving antibiotics, when compared against other aetiologies such as acute respiratory infections.

## Discussion

Our results suggest that an M72-like vaccine, when deployed as a post-exposure vaccine amongst adults and adolescents, could have an important impact on drug-resistant TB. Such a vaccine, acting alone, could avert 10% (95% CrI: 9.7–11%) of cumulative RR-TB cases over the next 15 years. Indeed, this impact is comparable to that of combining improvements in the management (detection and treatment) of RR-TB, projected to be 14% (95% CrI: 12–16%) of RR-TB cases over the same period (Table 2). The combined effect of vaccination and improved management is roughly additive (Figs. 3 and 5).

The impact of vaccination on relative reductions in RR-TB burden is roughly consistent across countries, reducing RR-TB cases between 5 and 16% (Fig. 2). Similar figures apply to relative reductions in RR-TB deaths (Fig. 4). In terms of country contributions to the projected global impact of vaccination, India and China are consistently the leading countries (Figs. 2 and 4), owing to their relatively large shares of global TB burden^2^. Countries in the former Soviet Union have the world’s highest rates of drug resistance amongst TB cases, however, their relative contributions to the global vaccine-avertable burden of RR-TB are lower, as a result of having smaller TB epidemics in absolute terms^2^.

We have presented results for the number of second-line TB treatments that would be avertable by a TB vaccine between 2020 and 2035, but a cost impact is outside the scope of this study. This is an important component of any costing analysis, because of the disproportionate cost of managing drug-resistant TB, for any national TB programme^20,21^. Notably, our results illustrate that—while vaccination could reduce the need for second-line drugs by reducing incidence of RR-TB—measures to improve the detection of RR-TB would have the opposite effect, by increasing the number of patients needing to be treated with second-line drugs (Supplementary Fig. 12). Appropriate TB treatment is critical for patient outcomes^7,22^. However, our results illustrate that a combined strategy—of vaccination with improved RR-TB management—could help to mitigate the budgetary burden on TB programmes, by reducing overall RR-TB burden while minimising the numbers of second-line treatments required (Table 2, Supplementary Fig. 12). Any resulting cost savings would release programmatic funds, potentially for other TB control efforts such as intensified case-finding that could—in turn—have a synergistic effect on reducing the burden of drug-resistant TB.

As a grounding example for our analysis, we have drawn data from the phase 2b trial of the M72/AS01_E_ vaccine, which was limited to individuals with evidence of latent TB infection. Accordingly, we modelled a scenario where a vaccine is deployed solely for post-exposure protection, i.e. to reduce the risk of TB disease amongst those already infected. We expect that incorporating pre-exposure protection, and broadening eligibility to include all individuals regardless of infection status, would substantially increase the projected impact of vaccination. Future trial data will be valuable in informing these projections. Another important area for future work is the feasibility of, and best implementation strategies for, the type of mass vaccination programme considered here. School-based vaccination may be important in reaching 15-year-olds, and is already used for the delivery of tetanus and HPV vaccines in low- and middle-income countries^23,24^. To ensure equitable access, however, such strategies will need to be supplemented by additional measures such as mobile vaccine delivery, to reach those not in the school system. Supplementary analysis illustrates the importance of mass vaccination campaigns amongst adults, showing that without these additional efforts, overall impact on RR-TB incidence and mortality would drop by 85% (Supplementary Table 2). In practice, such catch-up efforts would need to be planned carefully: the existing coverage of adult immunisation in countries such as India presents substantial challenges^25,26^, which would likely apply to a future TB vaccine as well. In both types of vaccination effort, there are important considerations around whether the vaccine should be offered only to those with evidence of LTBI, or regardless. In the present work, we have assumed the former, a choice necessitated by the available data: the recent phase 2b trial of the M72/AS01_E_ vaccine was restricted to those with LTBI^14^. As more data emerges about the effectiveness of the vaccine in uninfected individuals, an important area for further analysis would be the relative value of limiting vaccination coverage to those with LTBI (incurring the cost and effort of identifying these individuals), vs universal eligibility within defined age groups (incurring the cost of additional vaccinations). The per-dose cost of any future TB vaccine will likely be a driving factor in these considerations.

Another approach to prevention, currently part of WHO guidelines for programmatic management of TB, is the use of preventive therapy regimens amongst those known to have TB infection^27^. Although recent years have seen the emergence of shortened and simplified preventive therapy regimens^9,10^, these are rifampicin-based regimens, and thus unlikely to be effective against RR-TB infection. There is increasing recognition of the potential value of fluoroquinolone-based preventive regimens amongst contacts of those with known drug-resistant TB^28,29^ although there remains a pressing need for more clinical trials to demonstrate efficacy^8^. On the other hand, by preventing TB at the population level, not just amongst contacts of known cases, a future vaccine might have further-reaching impact than preventive therapy: however, a strategy, such as the one we have modelled here, may take several years to fully realise this impact (Supplementary Fig. 9). Any future strategy for TB prevention is, therefore, likely to require a combination of preventive therapy and vaccination.

In addition to mass vaccination, supplementary immunisation approaches may also have an important impact, for example, vaccination of close contacts of diagnosed TB cases. Recent modelling work (albeit in the context of preventive therapy) suggests that preventing 60% of incident TB amongst household contacts of TB cases in the WHO South-East Asian Region could reduce annual incidence rates in the Region by ~8%, by 2030^30^. That analysis assumed preventive therapy to be ineffective amongst those with drug-resistant infection; a vaccine in place of preventive therapy, therefore, might be expected to have a similar impact on RR-TB, if not higher. Our analysis also does not address the potential use of therapeutic vaccination, that is administered following completion of treatment for active TB, in order to minimise the risk of recurrence^31^. By potentially reducing the risk of treatment-acquired drug resistance developing into active, infectious TB, such approaches could also have important implications for future RR-TB incidence.

Our focus on post-exposure vaccination amongst adults and adolescents with LTBI is motivated by the recent phase 2b trial of the M72 vaccine^14^, which suggested a 50% reduction in incidence amongst those receiving the vaccine. However, these numbers should be interpreted with caution, primarily because they come from a trial that was only designed to assess safety and immunogenicity, not efficacy; larger trials are needed to estimate protective efficacy with precision. Moreover, the protective effect was seen mostly after a year, in males and those under 25 years of age. The study was conducted in South Africa, Kenya and Zambia, settings with a high overall burden of TB (for example with South Africa having an annual TB incidence of 520 per 100,000 population^2^), and it is unclear how these findings might generalise to other settings. Therefore, phase 3 trial results in different settings will be invaluable in gaining more reliable estimates of efficacy. These limitations notwithstanding, our modelling results would apply generally to any post-exposure vaccine having 50% efficacy amongst adults and adolescents. For the purpose of our current study, the important findings reported in ref. ^14^ show that such a vaccine is indeed plausible.

As with any modelling analysis, our work has some limitations to note. For simplicity, and to cover 30 countries in a systematic way, we captured key features of TB transmission with four different model structures, using country-specific calibrations. Some model parameters are subject to substantial uncertainty, such as the quality of TB care in the private healthcare sector; we have aimed to address this uncertainty by adopting wide uncertainty intervals for these parameters (Supplementary Table 5), and by propagating this uncertainty to model projections using Bayesian calibration methods. For simplicity we adopted a simple model mechanism for ageing, assuming a constant per-capita hazard of transitioning between child and adult strata. This simplification allows us to accommodate additional, country-specific complexities such as public and private healthcare providers, and the role of HIV in driving TB dynamics. Nonetheless, our results for TB impact remain broadly consistent with those reported in earlier studies which use more complex demographic structure^12^. In the case of China, where most TB burden is concentrated in those aged over 65 years, additional sensitivity analysis suggests that model results do not change substantially, with the inclusion of a third age group to represent the elderly (Supplementary Fig. 14). Our model also entails simplifications around HIV and TB, adopting a relatively simple HIV care cascade (Supplementary Fig. 15c), as well as borrowing from previous work^32^ to incorporate simplified scenarios for the scale-up and maintenance of antiretroviral treatment (ART) (Supplementary Methods 1). Sensitivity analysis suggests that the influence of HIV on effectiveness of a future TB vaccine would have important implications for vaccine impact in countries with high HIV burden but would not substantially change our estimates for vaccine impact at the global level (Supplementary Table 2).

Such limitations notwithstanding, the essential findings of our analysis are likely to hold true: that an M72-like vaccine, even with an efficacy of only 50%, at 72–76% coverage of LTBI adolescents and adults, would have an important impact on the global burden of RR-TB, as well as important synergistic effects with ongoing efforts to improve the detection and treatment of RR-TB. As with many challenges in TB control, optimal control strategies in the future are likely to involve a combination of different approaches: our work illustrates the critical role that vaccination could play, as part of these strategies.

## Methods

### Model structure

We limited our analysis to the 30 countries shown in Table 1 and Supplementary Fig. 16, which together accounted for 90% of global RR-TB incidence in 2018^2^. To account for the wide variation in TB epidemiology and health systems between these different countries, we developed four different TB models, relating to each of the categories shown in Table 1, and described in further detail in Supplementary Methods 1. For countries where HIV is a driving factor in TB burden (Supplementary Fig. 17), we modelled the historical dynamics of HIV using data obtained from The Joint United Nations Programme on HIV/AIDS (UNAIDS)^33^. We also modelled the impact of HIV on TB progression, as well as the mitigating effect of ART and isoniazid preventive therapy for TB (assuming such preventive therapy to be effective only against drug-sensitive infection). We did not explicitly model the dynamics of HIV and ART, instead taking these as model inputs. For countries where the private sector has a substantial presence, we distinguished between those receiving care in the public and private sectors, and assumed a lower standard of TB care in the latter than in the former^34–37^. Given little systematic data for care provided by the private sector in different countries, we adopted broad uncertainty intervals for the accuracy of TB diagnosis and for treatment completion rates in this sector.

For each of four TB models we stratified the population into those above and below 15 years of age, and modelled ageing as a constant rate-of-transition between these strata, chosen to capture the country demographics as of 2018^38^. Model equations and parameters can be found in Supplementary Methods 1, Supplementary Fig. 15, and Supplementary Tables 5, 6.

### Data and calibration

For each country we calibrated the model to a range of epidemiological and programmatic indicators, including the WHO estimates for TB incidence and mortality, stratified by age group; the proportion of incident TB cases being RR-TB; and the proportion of TB incidence that occurred in those coinfected with HIV (Supplementary Table 7). For countries having a strong private sector healthcare presence, we assumed that all those surviving to the point of care-seeking are managed by either the public or private sector: by combining data for public sector notifications with model projections for incidence and mortality, therefore, we aimed to estimate the proportion of patients being managed by the public vs private sectors. For countries with a driving role of HIV in the TB epidemic, we obtained ART coverage from UNAIDS, for simplicity assuming a linear scale-up to current levels^32^. We calibrated the model using Adaptive Markov Chain Monte Carlo^39^, which allows systematic propagation of uncertainty from model inputs to model projections, while updating the proposal distribution at each iteration. We reduced autocorrelation by taking every 150th sample and discarded the first 20,000 samples, to yield 200 samples overall (Supplementary Methods 2). We calculated 2.5th and 97.5th percentiles as the 95% credible intervals, and the 50th percentile as point estimates, on these samples and on model projections. As a form of model validation, we compared model projections for TB prevalence to findings from prevalence surveys, where available. For any countries where model-projected prevalence did not match those from the prevalence surveys (in particular, where the uncertainty intervals did not overlap), we recalibrated the models using the prevalence survey data, to ensure consistency with all available information.

### Intervention simulations

Consistent with the WHO preferred product characteristics^13^ and the outcome of the M72/AS01_E_ trial^14^, we assumed that the effect of the vaccine is to attenuate the rates of primary progression and reactivation of latent TB amongst adults and adolescents, thereby reducing TB incidence rates amongst those vaccinated by 50%. We did not consider the vaccine protection against recurrence of TB. We assumed an average duration of protection of 10 years, and that after this period, vaccinated individuals revert to their unvaccinated status of LTBI. In the main analysis, we assumed for simplicity that the effectiveness of a future TB vaccine would be unaffected by the HIV status of recipients. Currently, there is no evidence for the effect of HIV coinfection on the performance of M72-like vaccines, as the recent phase 2b trial was limited to HIV-negative individuals. However, the RTS,S malaria and pneumococcal conjugate vaccines offer examples of vaccines whose effectiveness is impaired by HIV^40,41^. Therefore, as a sensitivity analysis, we modelled a scenario where a future TB vaccine has half the efficacy amongst HIV positive individuals, compared to those without HIV.

We assumed a routine TB immunisation programme which vaccinates adolescents on their 15th birthday from 2025 to 2035, with country-specific vaccination coverage derived from secondary school attendance^42^ (Supplementary Fig. 18). In addition, to boost vaccine coverage among all adults over 15 years old, we assumed 2-year catch-up campaigns, implemented every 5 years, with a peak coverage of 72–76%, according to WHO region^12^. The peak coverage of these campaigns was derived from earlier modelling work^12^, where the data were extracted from previous rubella vaccination campaigns and adapted to a wider target population of adults and adolescents. We projected the averted cumulative incidence of RR-TB; the averted cumulative deaths; and the averted patient-months of second-line TB treatment over 2020–2035. We assessed these outcomes with respect to a ‘status quo’ comparator, assuming current TB services continue indefinitely without change. We also considered an ‘improved RR-TB management’ comparator, reflecting ongoing improvements in the management of RR-TB, in particular assuming that 85% of TB diagnoses also receive drug susceptibility testing for rifampicin resistance (for example, through the increased use of molecular diagnostic tools^19^), and that second-line treatment success is increased to 75% (resulting from adoption of new, second-line regimens^5^), from 2020 onwards. Supplementary Fig. 10 shows these indicators of RR-TB management in relation to the levels currently reported by different countries.

### Potential contribution to control of antibiotic consumption

Unnecessary antibiotic consumption is a key driver of antimicrobial resistance, more broadly than TB. We additionally examined the potential for a future TB vaccine to reduce this consumption through the bystander effect^43^: that is, as a result of reducing symptomatic presentations for primary care, and thus reducing instances of empiric antibiotic treatment. We used data from Demographic Health Surveys (DHS)^44^, for the proportion of acute respiratory illness that is medically attended and treated with antibiotics in different settings, together with estimates from the Global Burden of Disease 2017 project^45^ for all-cause incidence of respiratory disease in the same countries as those covered in the DHS data (Supplementary Methods 3).

### Reporting summary

Further information on research design is available in the Nature Research Reporting Summary linked to this article.

## Supplementary information

Supplementary Information

Reporting Summary

## Data Availability

Country-specific data used in this study are publicly available and their sources are summarised in Supplementary Table 7, including population statistics from the United Nations World Prospect Project 2019 (http://population.un.org/wpp/); TB incidence, mortality, and other programmatic indicators from the WHO Global Tuberculosis Database (http://www.who.int/tb/data/en/); HIV prevalence and ART coverage from the data tool of UNAIDS (http://aidsinfo.unaids.org/); and secondary school attendance from the United Nations Children’s Fund (https://data.unicef.org/topic/education/secondary-education/). For analysis of antibiotic consumption for cough-related diseases, data were obtained from the DHS Program (http://dhsprogram.com/data/, through the rdhs 0.6.3 package in R 4.0.2) and the Global Burden of Disease Study 2017 (http://ghdx.healthdata.org/gbd-2017). Extracted data are also presented in Supplementary Table 7 or available at https://github.com/hfu915/drtb_vacc. Source data are provided with this paper.

## Source data

Source Data

## Code availability

MATLAB codes for simulating TB dynamics and vaccine impacts are available at https://github.com/hfu915/drtb_vacc.
