# Supplementary material for: Modelling the global burden of drug-resistant tuberculosis avertable by a post-exposure vaccine
Source: Nat Commun. 2021 Jan 18;12:424. doi: 10.1038/s41467-020-20731-x (PMC7814030; doi:10.1038/s41467-020-20731-x)
Supplement: Supplementary file 1 — Supplementary Information [file 41467_2020_20731_MOESM1_ESM.pdf]

SUPPLEMENTARY INFORMATION

Modelling the global burden of drug-resistant tuberculosis avertable by a post-exposure vaccine

Han Fu[1], Joseph Lewnard[2], Isabel Frost[3,4], Ramanan Laxminarayan[3,5], Nimalan Arinaminpathy[1]

[1] MRC Centre for Global Infectious Disease Analysis; and the Abdul Latif Jameel Institute for Disease and Emergency Analytics (J-IDEA), School of Public Health, Imperial College London, London W2 1PG, UK. [2]Division of Epidemiology, School of Public Health, University of California, Berkeley, Berkeley, California 94720, USA. [3] Center for Disease Dynamics, Economics & Policy, New Delhi, India. [4] Department of Infectious Disease, Imperial College London, London W2 1NY, UK.[5] Princeton Environmental Institute, Princeton University, Princeton, New Jersey 08544, USA.

This file contains:

- Supplementary Figs. 1-18 (page 2-16)
- Supplementary Tables 1-8 (page 17-30)
- Supplementary Methods 1-3 (page 31-42)
- Supplementary References (page 43-44)

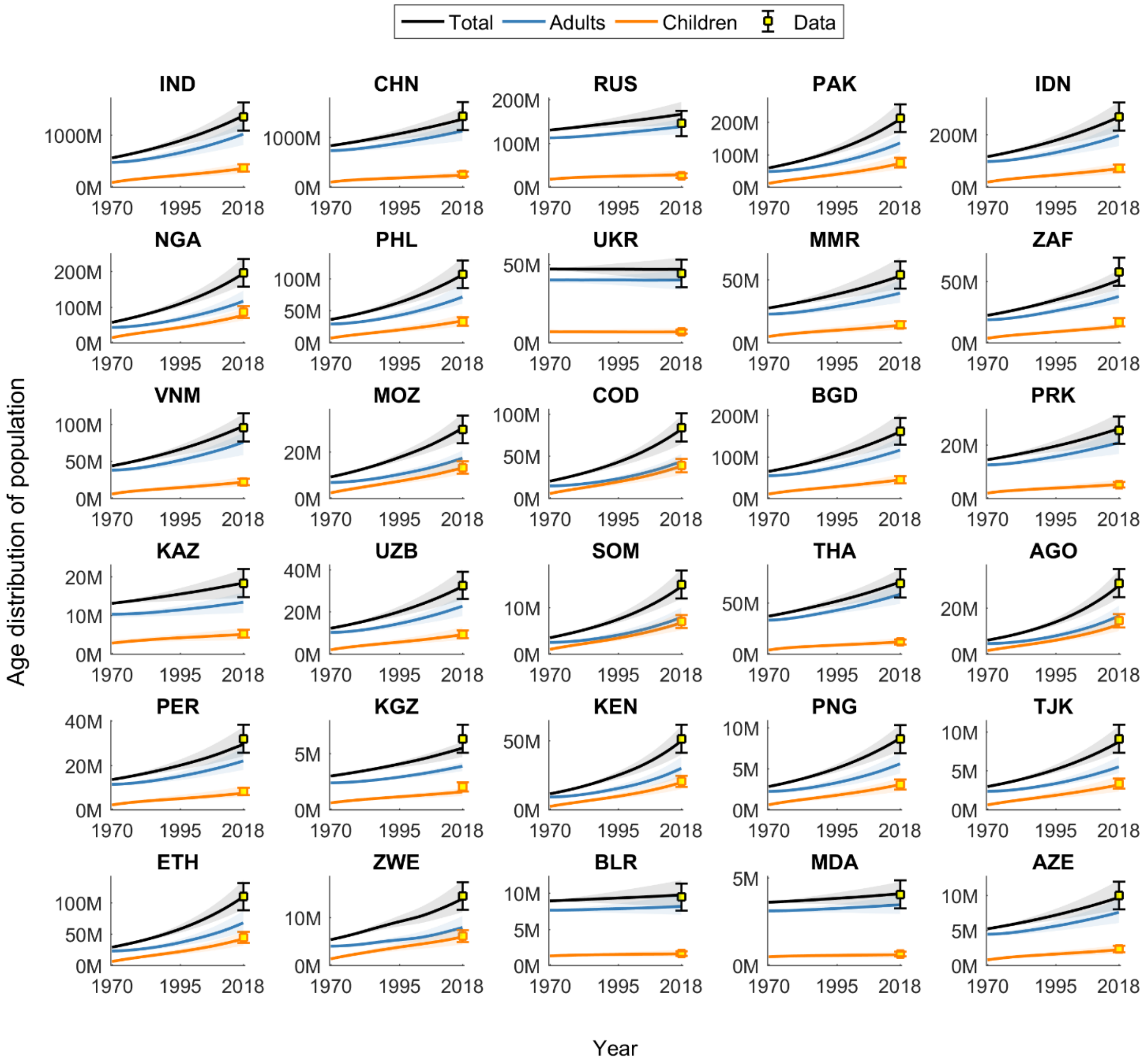

Supplementary Fig. 1. Population size and age distribution, 1970-2018.

n = 200 posterior samples. Median (solid lines) and 95% credible intervals (coloured bands) of model results are presented, including total (black), child (orange), and adult (blue) populations over 1970-2018. Point estimates (squares) and 95% uncertainty ranges (error bars) of country-specific calibration targets are shown. The calibration targets and ISO alpha-3 country codes are listed in Supplementary Table 7.

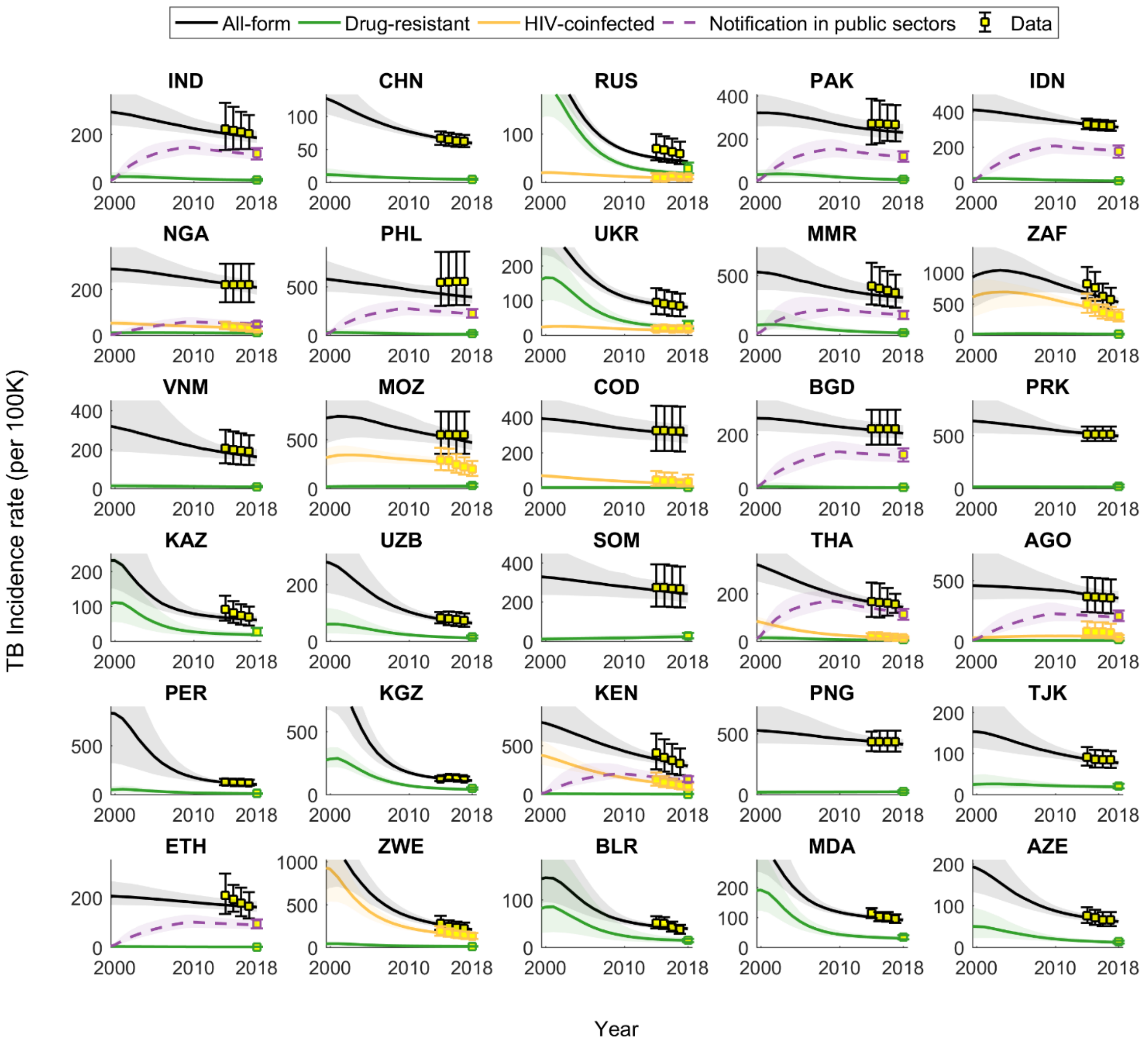

Supplementary Fig. 2. Calibration results of TB incidence rates, 2000-2018.

n = 200 posterior samples. Median (solid lines) and 95% credible intervals (coloured bands) of model results are presented, including incidence rates of all-form TB (black), RR-TB (green), and HIV-TB (yellow) per 100,000 population over 2000-2018. Purple dashed lines represent the notification rates of all-form TB per 100,000 population, presented only for those countries listed as having a strong private healthcare sector in Table 1. Point estimates (squares) and 95% uncertainty ranges (error bars) of country-specific calibration targets are shown. The calibration targets and ISO alpha-3 country codes are listed in Supplementary Table 7. Abbreviations: HIV-human immunodeficiency virus, RR-TB-rifampicin-resistant tuberculosis.

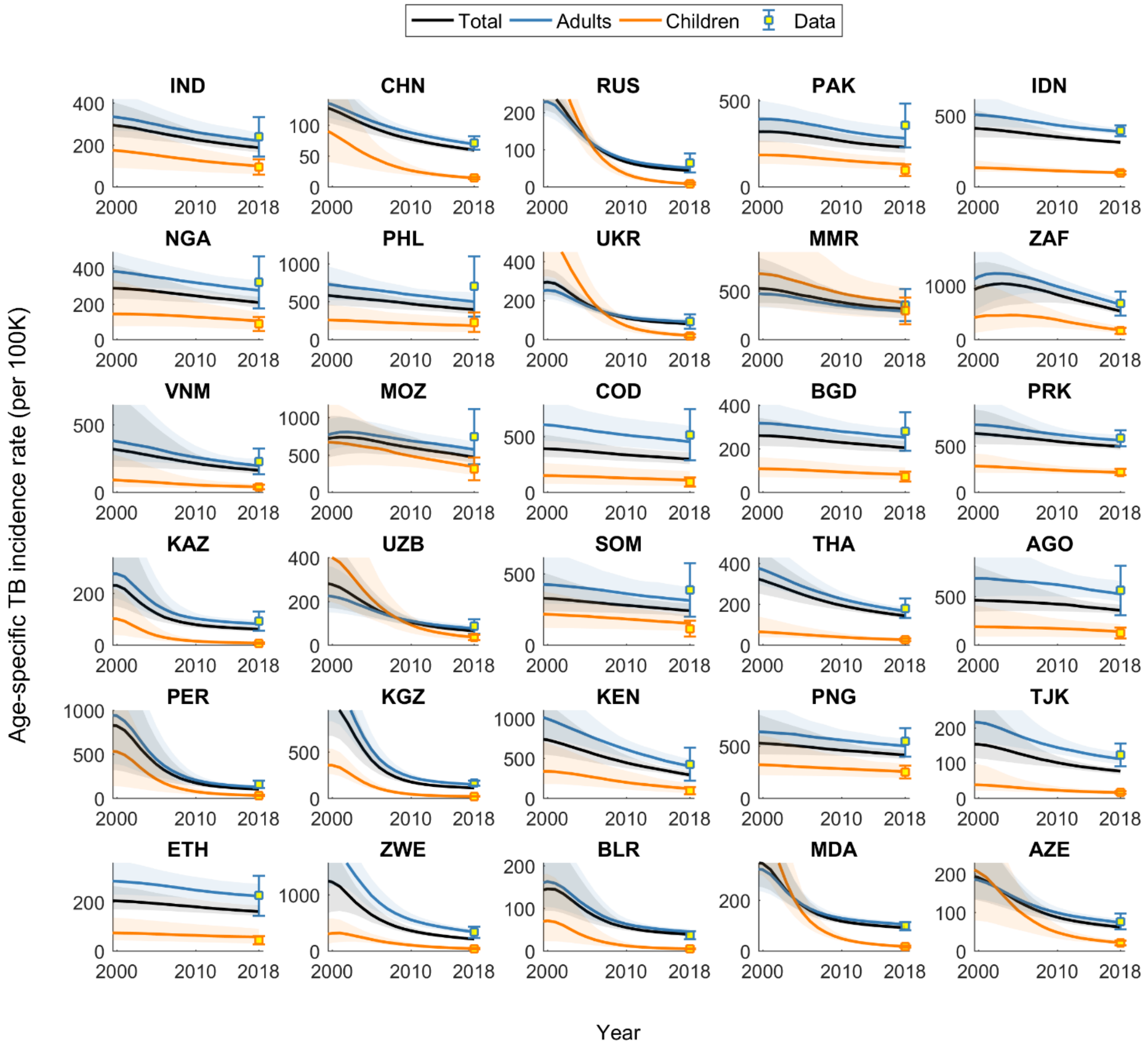

Supplementary Fig 3. Calibration results of age-specific TB incidence rates, 2000-2018.

n = 200 posterior samples. Median (solid lines) and 95% credible intervals (coloured bands) of model results are presented, including total (black), child-specific (orange), and adult-specific (blue) TB incidence rates per 100,000 population over 2000-2018. Point estimates (squares) and 95% uncertainty ranges (error bars) of country-specific calibration targets are shown. The calibration targets and ISO alpha-3 country codes are listed in Supplementary Table 7. Abbreviation: TB-tuberculosis.

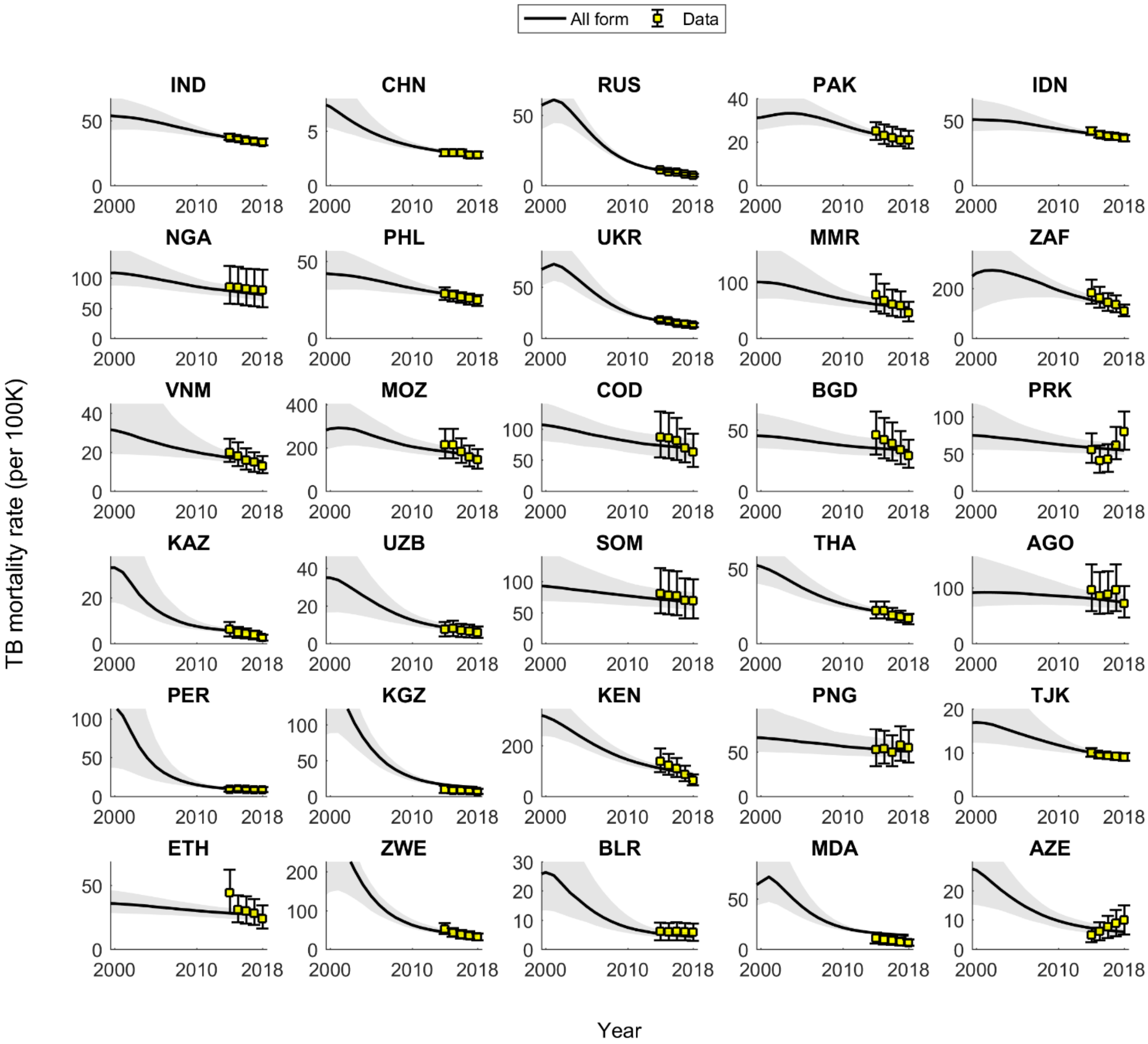

Supplementary Fig. 4. Calibration results of TB mortality rates, 2000-2018.

n = 200 posterior samples. Median (solid lines) and 95% credible intervals (coloured bands) of model results for all-form TB mortality rates per 100,000 population are presented. Point estimates (squares) and 95% uncertainty ranges (error bars) of country-specific calibration targets are shown. The calibration targets and ISO alpha-3 country codes are listed in Supplementary Table 7. Abbreviation: TB-tuberculosis.

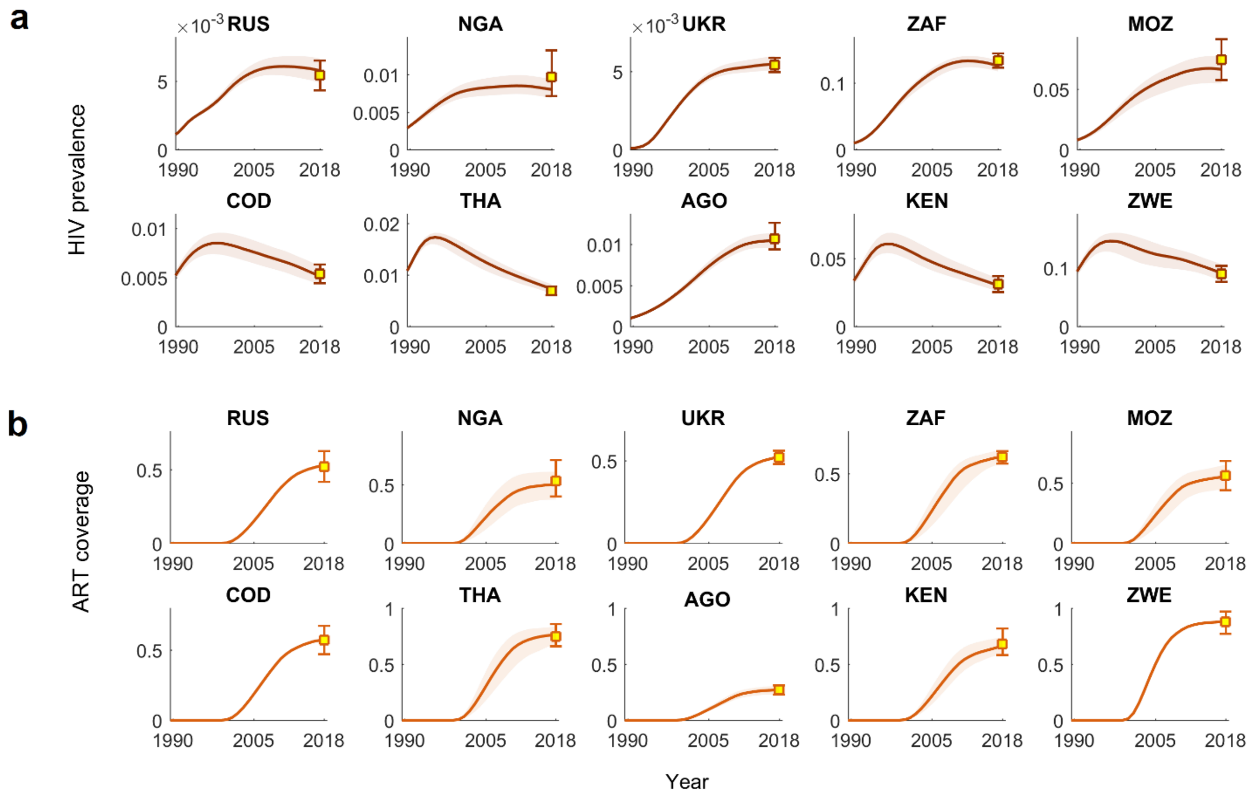

Supplementary Fig. 5. Calibration results of HIV prevalence and ART coverage, 1990-2018.

n = 200 posterior samples. Median (solid lines) and 95% credible intervals (coloured bands) of model results are presented. Point estimates (squares) and 95% uncertainty ranges (error bars) of country-specific calibration targets are shown. The calibration targets and ISO alpha-3 country codes are listed in Supplementary Table 7. **a** Brown lines represent HIV prevalence. **b** Orange lines show the increasing trend of ART coverage among people living with HIV. Only countries with more than 10% of HIV-infected TB cases are incorporated with the HIV model structure (Supplementary Fig. 17). Abbreviations: ART-antiretroviral treatment, RR-TB-rifampicin-resistant tuberculosis, HIV-human immunodeficiency virus.

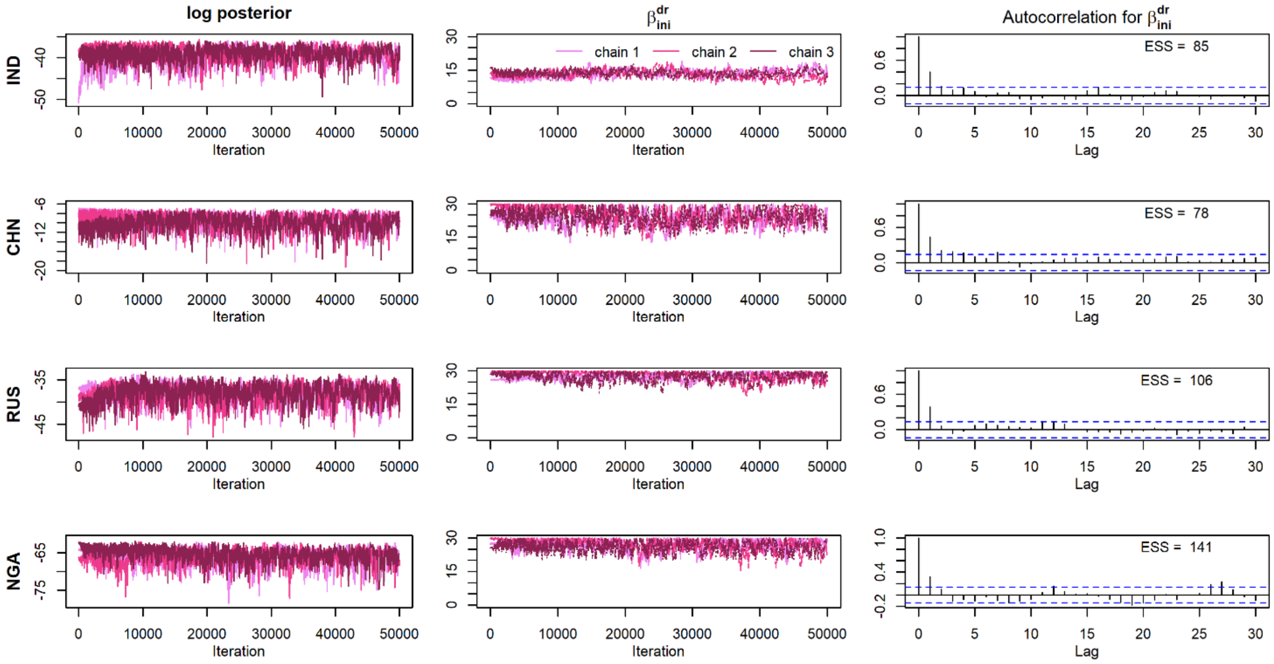

Supplementary Fig. 6. MCMC diagnostics for India (IND), China (CHN), Russian Federation (RUS), and Nigeria (NGA).

In each row, we show diagnostics for model calibration in a country. The countries with the highest absolute burden of RR-TB from each of the model categories listed in Table 1 are included. In the first column, log-posterior density obtained from three independent chains are presented, followed by traces of drug-resistant infection rate ($\beta_{\mathrm{ini}}^{\mathrm{dr}}$). The last column further shows autocorrelation ESS for this parameter using the 200 posterior samples. Abbreviations: ESS-effective sample size, MCMC-Markov chain Monte Carlo, RR-TB-rifampicin-resistant tuberculosis.

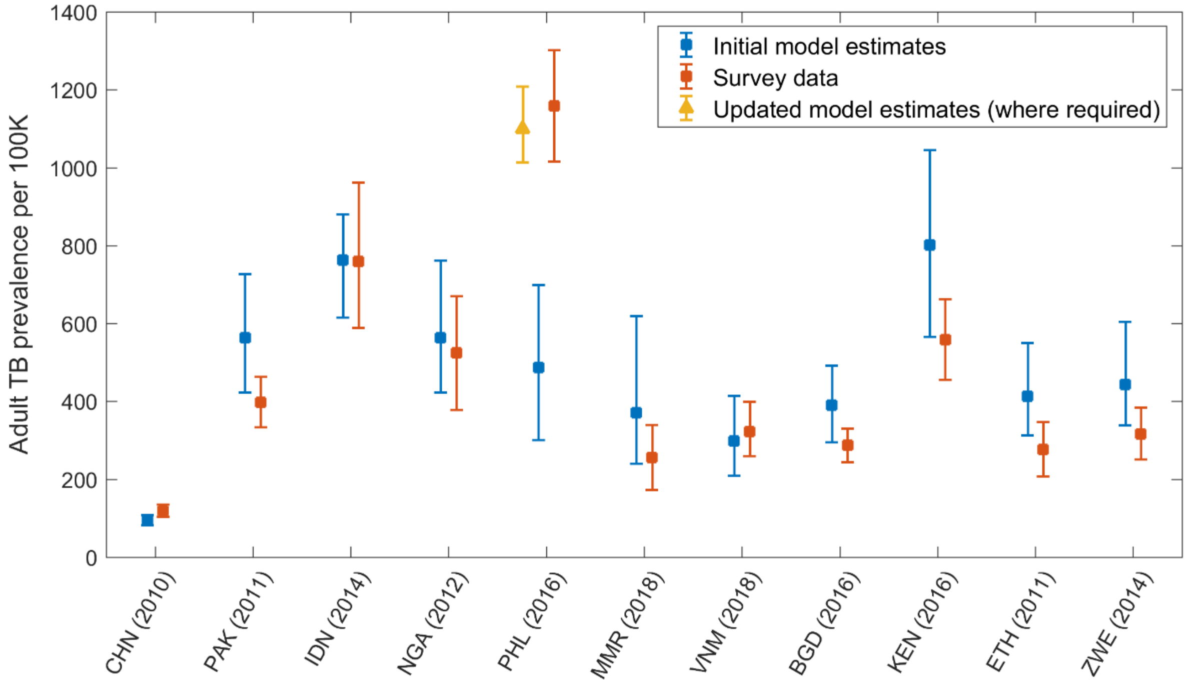

Supplementary Fig. 7. Comparison of TB prevalence rate among adult population.

Median (squares) and 95% uncertainty intervals (error bars) of adult TB prevalence per 100,000 population are presented. Initial model projections (*n* = 200 posterior samples, independent of survey data) are shown in blue, for comparison against survey data (*n* varies by survey, from nationally representative populations) in red [3-13]. For the latter, the year of the survey is labelled in the x-axis labels. Although there is broad agreement across countries between model and survey data, in certain instance (Philippines) initial model-based estimates did not contain the point prevalence estimates within their uncertainty intervals. For this country we recalibrated the models using the prevalence data: resulting estimates are shown by the yellow bar. The ISO alpha-3 country codes are listed in Supplementary Table 7.

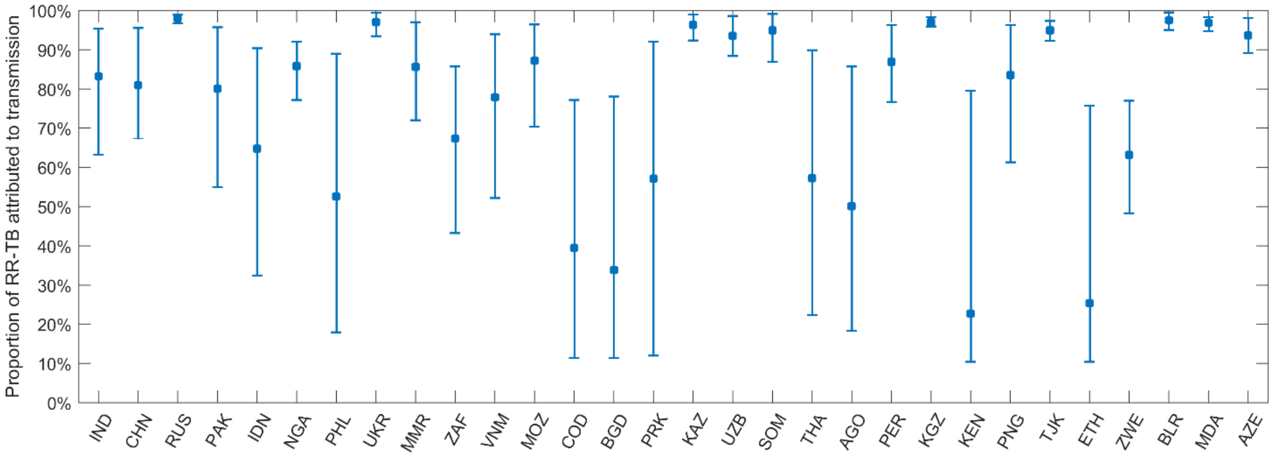

Supplementary Fig. 8. Estimated proportion of RR-TB incidence arising through transmission in 2018.

n = 200 posterior samples. Median (squares) and 95% credible intervals (error bars) of model results are presented. The ISO alpha-3 country codes are listed in Supplementary Table 7. Abbreviation: TB-tuberculosis.

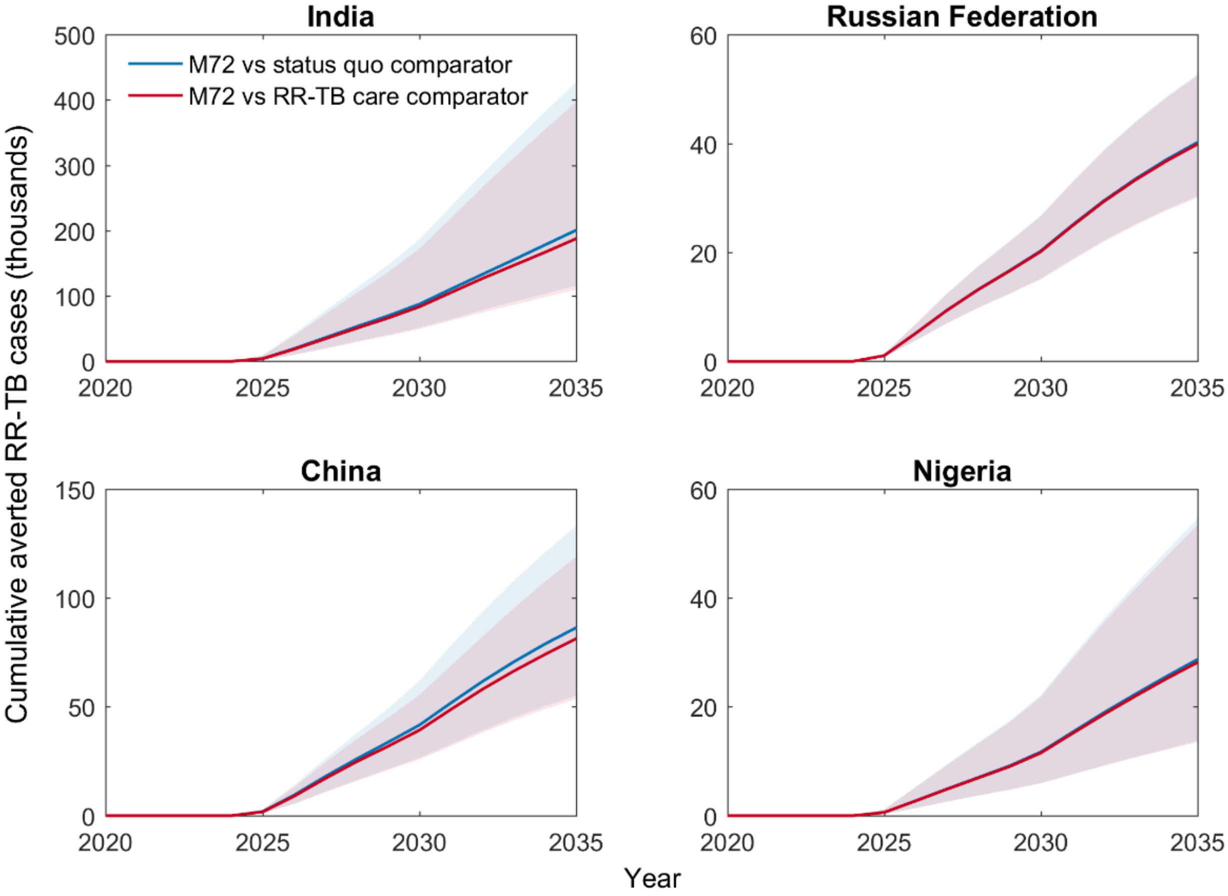

Supplementary Fig. 9. Cumulative averted RR-TB cases attributed to vaccination over 2020-2035, relative to corresponding comparators.

n = 200 posterior samples. Median (solid lines) and 95% credible intervals (coloured bands) of model projections for cumulative averted RR-TB cases over 2020-2035 are presented. The countries with the highest absolute burden of RR-TB from each of the country categories listed in Table 1 are included. As Fig. 1 in the manuscript shows RR-TB incidence trends in each of the four scenarios (status quo, improved RR-TB management, vaccination, vaccination in combination with improved RR-TB management), we particularly present the cumulative number of averted cases by a TB vaccine in pairwise comparison, using a status quo (blue) and an improved RR-TB improvement (red) comparators, respectively. Abbreviation: RR-TB-rifampicin-resistant tuberculosis.

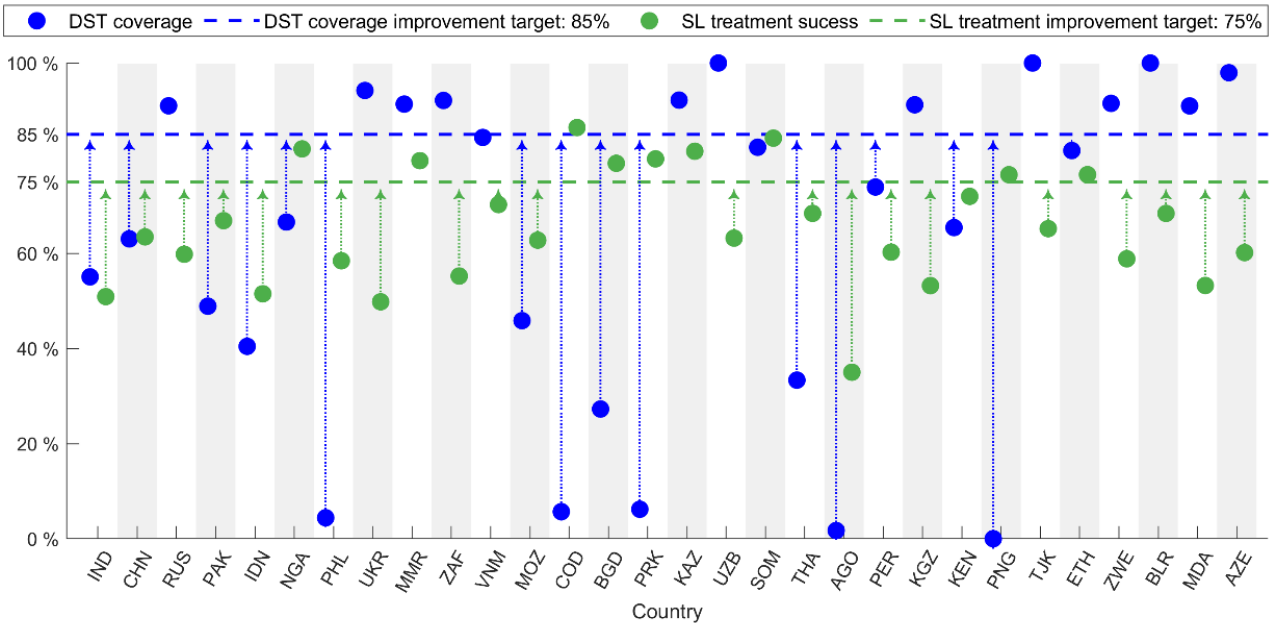

Supplementary Fig. 10. Country-specific DST coverage and SL treatment success.

Blue and green circles represent the current DST coverage and SL treatment success in each country reported in the Global TB database of the World Health Organization [1]. We modelled this ongoing improvement by linearly increasing the proportion of TB patients receiving DST to 85% and the SL treatment success to 75% over 2020-2022, as shown in blue and green horizontal dashed lines, respectively. Length of a vertical dotted line from a circle to the corresponding target horizontal line indicates the gap of improvement. For countries showing above-the-target performance of RR-TB management, we assumed that current levels of DST coverage or/and SL treatment success would be maintained. We ranked countries by the incident cases of rifampicin-resistant TB in 2018, using the ISO alpha-3 codes listed in Supplementary Table 7. Abbreviations: DST-drug susceptibility test, SL-second-line.

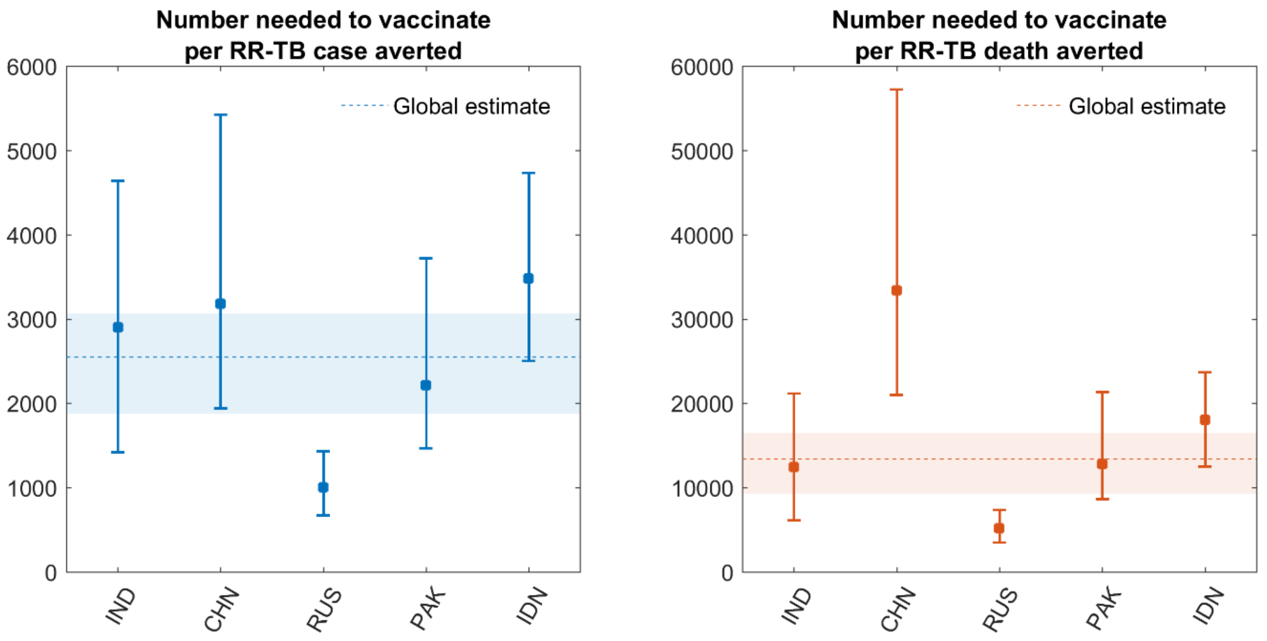

Supplementary Fig. 11. Number of LTBI adolescents and adults needed to vaccinate per RR-TB case or death averted.

n = 200 posterior samples. Median (squares) and 95% credible intervals (error bars) of model results are presented. Five countries contributing to largest number of RR-TB cases in 2018 are included. Horizontal dashed lines and coloured bands show the estimates at the global level. The ISO alpha-3 country codes are listed in Supplementary Table 7. Abbreviation: RR-TB-rifampicin-resistant tuberculosis.

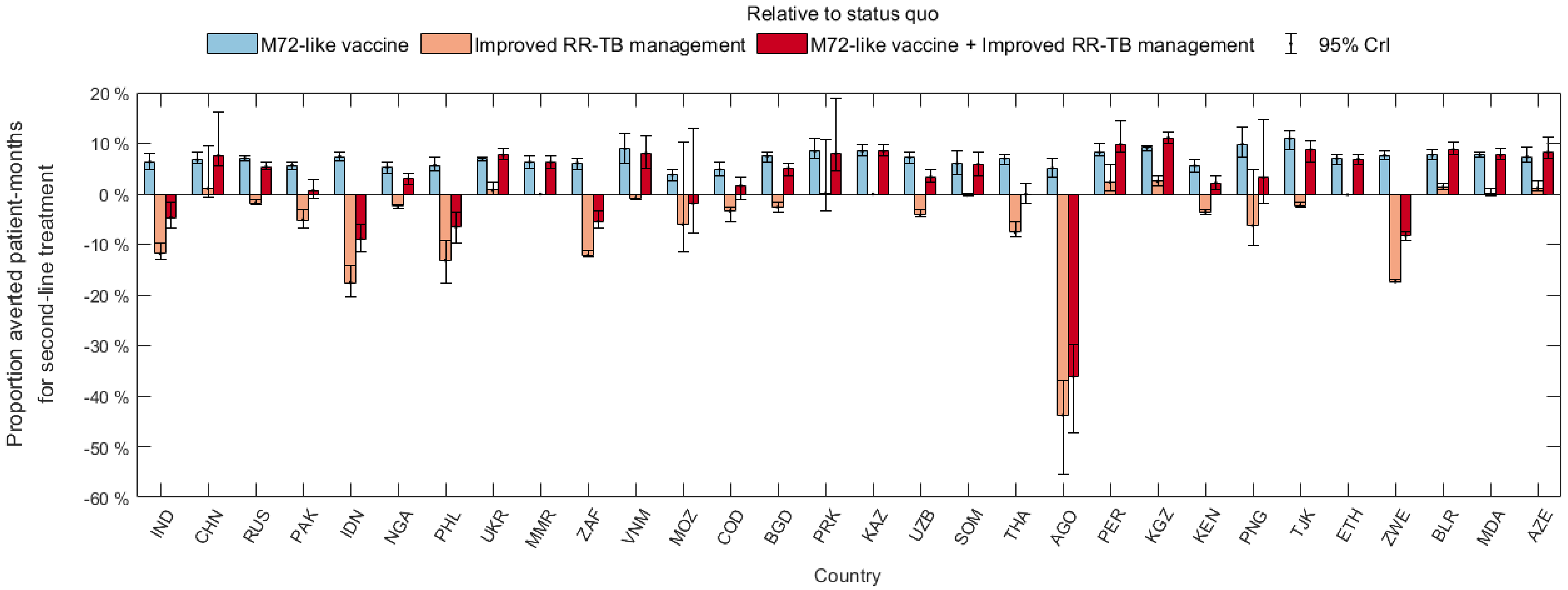

Supplementary Fig. 12. Averted proportions of patient-months for second-line treatment by country.

n = 200 posterior samples. Median (rectangular bars) and 95% CrIs (error bars) of model results are presented. The averted proportions of patient-months for second-line treatment by different scenarios are compared to a status quo baseline. Negative values indicate increase in patient-months for RR-TB treatment, as a result of enhanced coverage and completion of second-line treatment. As described in the manuscript, we assumed a M72-like vaccine that provides 50% of post-exposure protection in the analysis. The ISO alpha-3 country codes are listed in Supplementary Table 7. Abbreviations: CrI- credible interval, RR-TB- rifampicin-resistant tuberculosis.

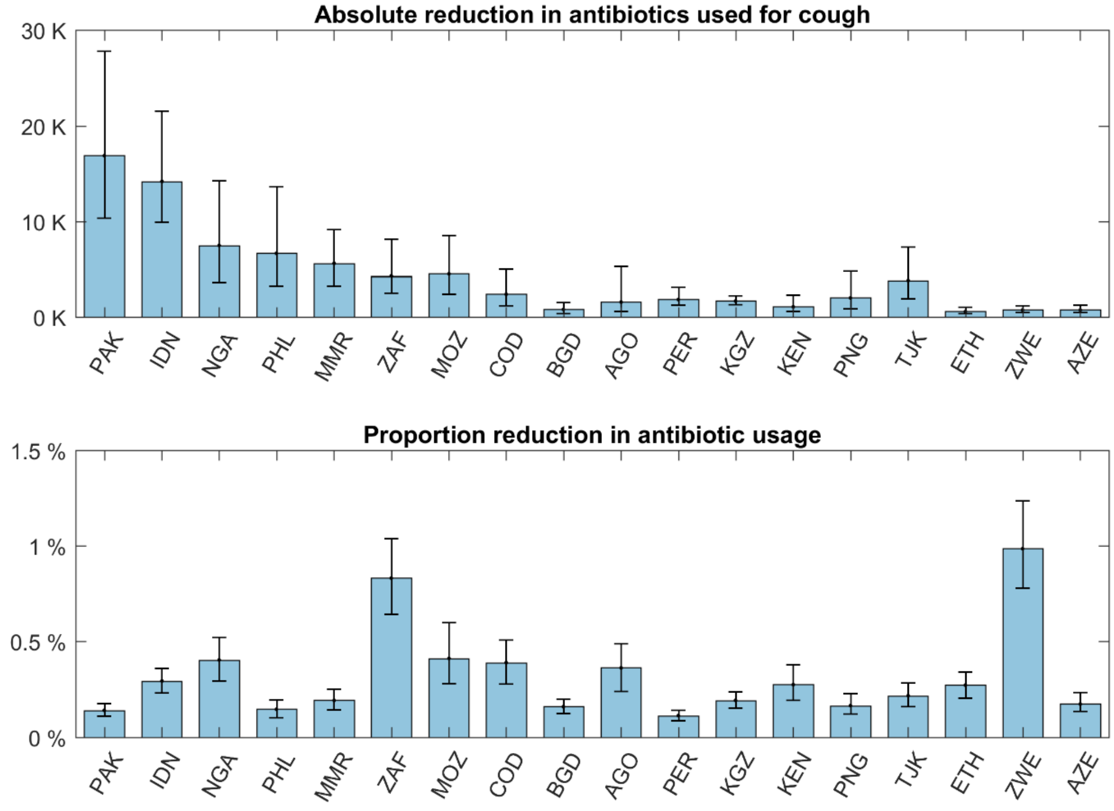

Supplementary Fig. 13. Absolute and proportion reduction in antibiotics used for cough by a post-exposure TB vaccine.

n = 200 posterior samples. Median (rectangular bars) and 95% credible intervals (error bars) of model results are presented. Only 18 countries with Demographic Health Surveys data were included in this analysis. The ISO alpha-3 country codes are listed in Supplementary Table 7.

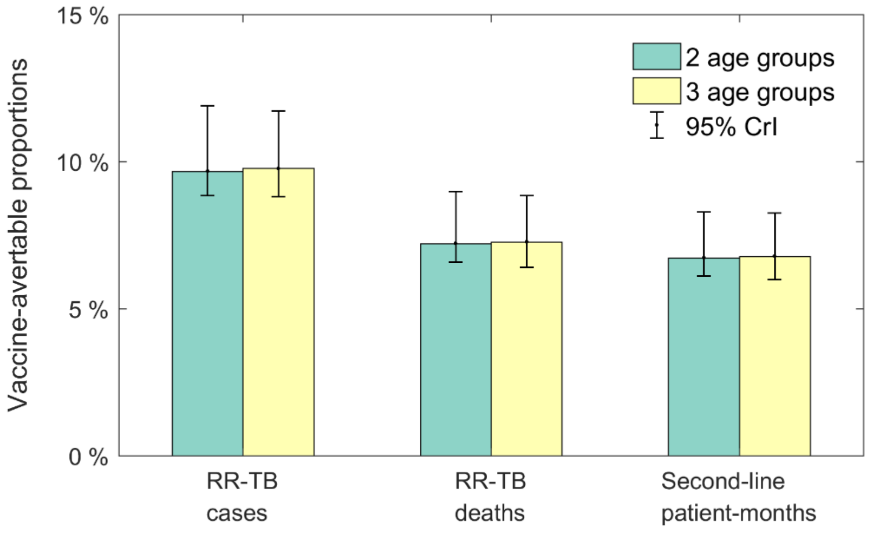

Supplementary Fig. 14. Averted proportion of RR-TB burden by age structure in China.

n = 200 posterior samples. Median (rectangular bars) and 95% CrIs (error bars) of model results are presented. Considering the elderly-concentrated TB epidemic in China, we developed a three-group age structure that further divides those who are older than 15 years old into adults (15-64) and elders (65+) populations. This three-group model (yellow bars), compared to the two-group model (green bars) in the main analysis, would result in similar vaccine-avertable impacts on RR-TB burden. Abbreviations: CrI- credible interval, RR-TB- rifampicin-resistant tuberculosis.

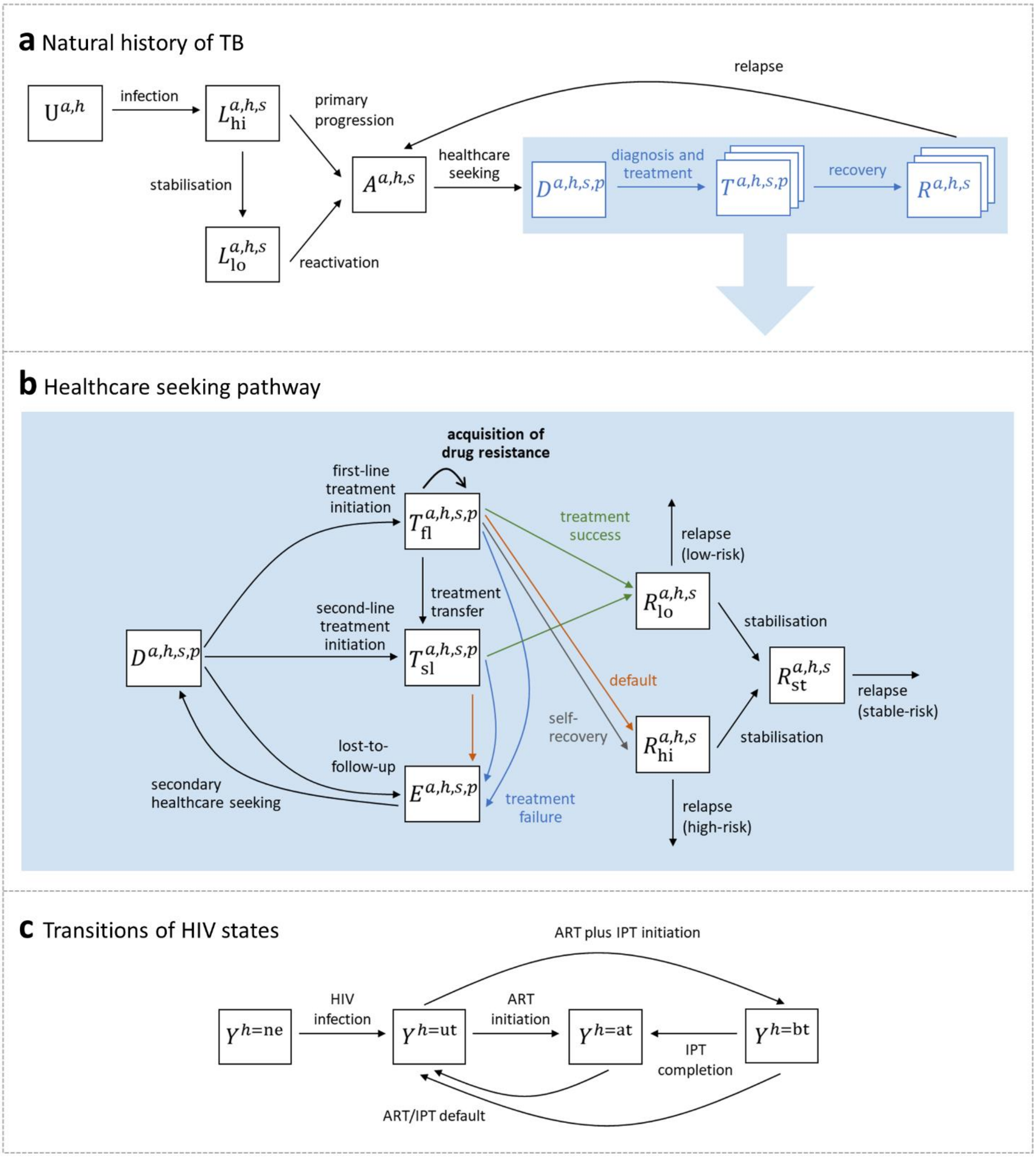

Supplementary Fig. 15. Schematic diagrams of the TB model.

a The natural history of TB is presented, with boxes showing the different states and arrows for the transitions between states. Boxes in the blue shading demonstrate the healthcare seeking pathway. **b** Details of healthcare seeking pathway is depicted. Coloured arrows represent different outcomes of treatment. **c** Simplified transitions of HIV states are shown. State Y indicates any state over the development of TB. Demographic changes such as birth, death, and ageing for clarity of presentation. Notations for disease states and risk structures are shown in Supplementary Table 1. Abbreviations: ART-antiretroviral treatment, HIV-human immunodeficiency virus, IPT-isoniazid preventive therapy, TB-tuberculosis.

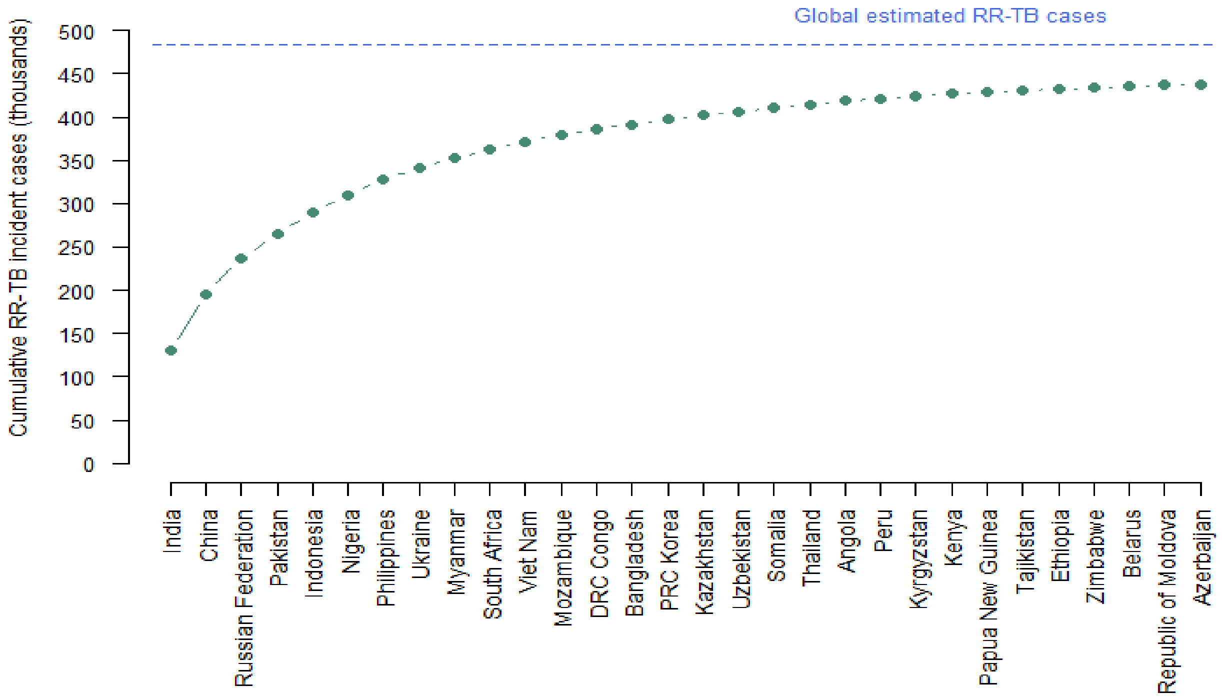

Supplementary Fig. 16. Cumulative RR-TB cases in 30 high-burden countries.

Countries are ranked in a descending order by the national number of incident RR-TB cases in 2018. The sum of RR-TB cases in these 30 countries contribute to 90% of the global burden. Abbreviation: RR-TB-rifampicin-resistant tuberculosis.

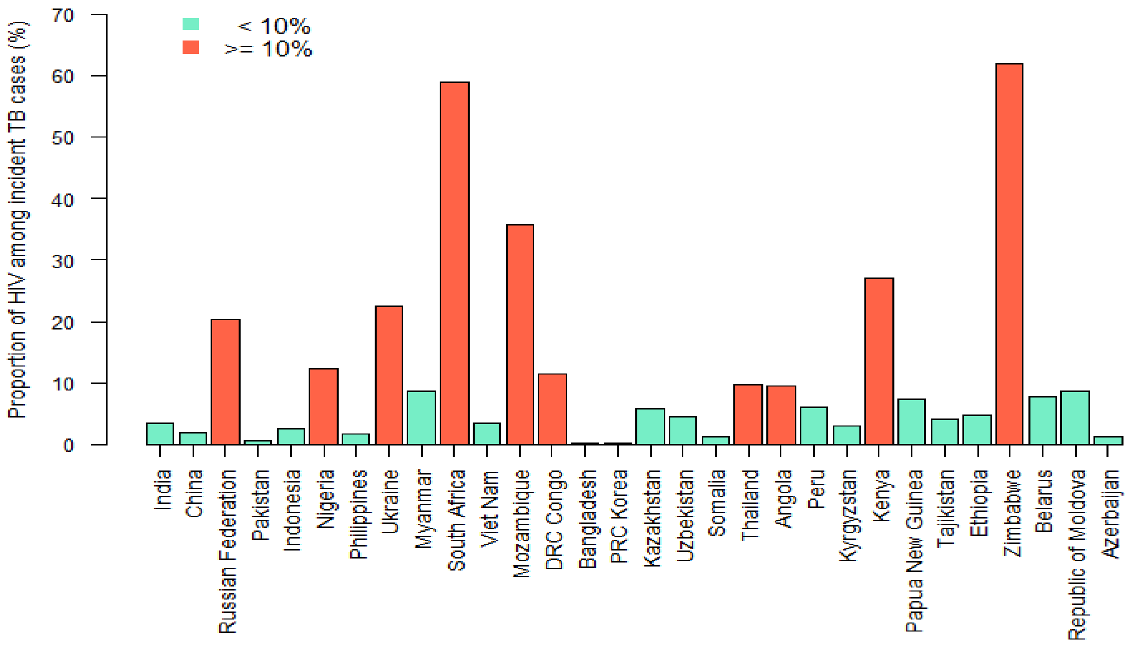

Supplementary Fig. 17. TB-HIV coinfection in 30 high-burden countries.

Bars represent the proportions of HIV infection among incident TB cases in 30 high-burden countries. Ten countries (orange) have ≥ 10% of TB cases HIV co-infection in 2018. Countries are ranked by the number of incident rifampicin-resistant TB cases in 2018. Abbreviations: HIV-human immunodeficiency virus, TB-tuberculosis.

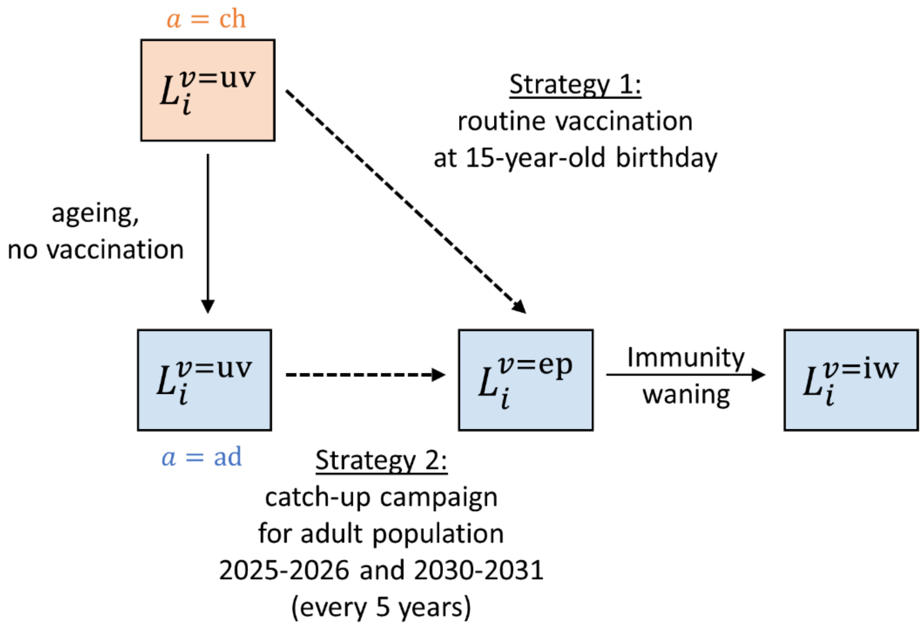

Supplementary Fig. 18. Model structure of vaccination strategies for a post-exposure TB vaccine.

In this modelling analysis, two vaccination strategies are involved: (i) A routine immunisation programme for children turning to 15 years old was adopted, combined with catch-up campaign targeting the adult population. (ii) Two rounds of catch-up campaigns are carried out over 2025-2026 and 2030-2031; in each of the two-year periods, the recruitment rate of vaccination increases linearly during the first year, and then declines during the second year, to reflect the scale-up of the programmes. Orange and blue boxes represent <=15 (ch) and > 15 years-old (ad) individuals, respectively. Three vaccination states, denoted as v, are included in the model: uv – unvaccinated, ep – vaccinated with effective protection, iw – vaccinated without effective protection due to immunity waning.

Supplementary Table 1. Posterior distributions of key model parameters and output.

Country	DS-TB infection rate, $\beta_{\mathrm{ini}}^{\mathrm{ds}}$	DR-TB infection rate, $\beta_{\mathrm{ini}}^{\mathrm{dr}}$	Initial care-seeking rate, $c_{1\mathrm{st}}$	Adult fast progression rate, $\rho_{\mathrm{hi}}^{\mathrm{ad},ne}$	Adult reactivation rate, $\rho_{\mathrm{lo}}^{\mathrm{ad},ne}$	Drug resistance acquisition rate, σ	Lifetime risk of TB disease among latent adults*
India	26 (17, 30)	15 (11, 21)	0.44 (0.26, 0.74)	0.053 (0.043, 0.063)	0.0013 (0.0007, 0.0015)	0.029 (0.0071, 0.048)	15% (12%, 17%)
China	26 (18, 30)	23 (15, 30)	2.3 (1.8, 3.0)	0.055 (0.043, 0.063)	0.0013 (0.0010, 0.0015)	0.033 (0.0082, 0.049)	16% (14%, 18%)
Russian Federation	13 (11, 17)	28 (25, 30)	4.7 (2.9, 6.6)	0.058 (0.048, 0.063)	0.0006 (0.0005, 0.0008)	0.038 (0.026, 0.050)	13% (11%, 14%)
Pakistan	29 (25, 30)	10 (7.3, 13)	0.88 (0.83, 1.0)	0.066 (0.057, 0.068)	0.0010 (0.0006, 0.0012)	0.031 (0.0065, 0.049)	16% (14%, 17%)
Indonesia	27 (19, 30)	9.3 (5.0, 14)	0.57 (0.45, 0.77)	0.055 (0.043, 0.063)	0.0012 (0.0006, 0.0015)	0.029 (0.0081, 0.049)	15% (11%, 17%)
Nigeria	25 (18, 30)	26 (19, 30)	0.26 (0.20, 0.39)	0.053 (0.043, 0.061)	0.0012 (0.0006, 0.0015)	0.045 (0.030, 0.050)	14% (11%, 15%)
Philippines	32 (23, 35)	7.0 (2.3, 12)	0.58 (0.41, 0.78)	0.056 (0.043, 0.063)	0.0012 (0.0006, 0.0015)	0.031 (0.0078, 0.049)	15% (12%, 17%)
Ukraine	12 (8.9, 16)	27 (22, 30)	6.3 (4.0, 7.7)	0.056 (0.043, 0.063)	0.0009 (0.0007, 0.0012)	0.028 (0.0074, 0.048)	14% (12%, 16%)
Myanmar	27 (20, 30)	12 (9.0, 17)	0.90 (0.71, 1.6)	0.057 (0.043, 0.063)	0.0012 (0.0006, 0.0015)	0.030 (0.0062, 0.049)	15% (12%, 17%)
South Africa	20 (11, 29)	23 (13, 30)	0.88 (0.38, 1.9)	0.054 (0.044, 0.063)	0.0010 (0.0006, 0.0015)	0.038 (0.018, 0.049)	14% (11%, 16%)
Viet Nam	26 (19, 30)	24 (14, 29)	0.89 (0.57, 1.6)	0.057 (0.044, 0.063)	0.0011 (0.0006, 0.0015)	0.031 (0.0078, 0.049)	15% (13%, 17%)
Mozambique	23 (15, 29)	27 (20, 30)	0.63 (0.20, 1.5)	0.057 (0.045, 0.063)	0.0011 (0.0006, 0.0015)	0.035 (0.012, 0.048)	15% (12%, 17%)
DR Congo	24 (14, 29)	24 (2.3, 30)	0.21 (0.11, 0.34)	0.055 (0.043, 0.063)	0.0012 (0.0005, 0.0015)	0.042 (0.026, 0.050)	14% (12%, 17%)
Bangladesh	28 (24, 30)	6.1 (1.2, 13)	0.66 (0.60, 0.83)	0.067 (0.055. 0.071)	0.0010 (0.0005, 0.0012)	0.030 (0.0089, 0.047)	16% (14%, 17%)
DPR Korea	28 (23, 30)	16 (2.9, 25)	0.61 (0.51, 0.81)	0.058 (0.049, 0.063)	0.0012 (0.0005, 0.0015)	0.032 (0.0069, 0.049)	15% (13%, 17%)
Kazakhstan	15 (9.2, 25)	32 (24, 35)	5.0 (2.8, 6.0)	0.057 (0.044, 0.063)	0.0012 (0.0007, 0.0015)	0.036 (0.011, 0.049)	15% (13%, 17%)
Uzbekistan	24 (18, 30)	27 (21, 30)	3.9 (1.7, 5.9)	0.055 (0.043, 0.063)	0.0013 (0.0008, 0.0015)	0.034 (0.0077, 0.049)	15% (13%, 18%)
Somalia	20 (13, 25)	27 (19, 30)	0.15 (0.05, 0.35)	0.056 (0.051, 0.063)	0.0011 (0.0005, 0.0015)	0.035 (0.0081, 0.049)	14% (11%, 16%)
Thailand	28 (22, 30)	14 (4.5, 22)	0.95 (0.74, 1.2)	0.045 (0.042, 0.056)	0.0010 (0.0006, 0.0015)	0.035 (0.011, 0.049)	13% (11%, 16%)
Angola	24 (16, 30)	21 (2.8, 29)	0.30 (0.19, 0.50)	0.067 (0.050, 0.078)	0.0009 (0.0005, 0.0015)	0.042 (0.022, 0.050)	15% (12%, 17%)
Peru	27 (21, 30)	20 (17, 28)	2.9 (1.2, 5.4)	0.058 (0.043, 0.065)	0.0011 (0.0009, 0.0012)	0.033 (0.0093, 0.049)	15% (13%, 17%)
Kyrgyzstan	30 (24, 34)	33 (30, 35)	6.3 (5.5, 9.4)	0.057 (0.045, 0.062)	0.0014 (0.0013, 0.0014)	0.041 (0.023, 0.050)	16% (15%, 17%)
Kenya	27 (21, 30)	8.3 (1.8, 25)	0.40 (0.33, 0.56)	0.068 (0.051, 0.079)	0.0007 (0.0005, 0.0010)	0.035 (0.013, 0.049)	15% (12%, 17%)
Papua New Guinea	27 (17, 30)	21 (14, 27)	0.65 (0.51, 1.0)	0.057 (0.046, 0.063)	0.0012 (0.0006, 0.0015)	0.034 (0.0071, 0.050)	15% (12%, 17%)
Tajikistan	24 (19, 28)	33 (28, 35)	1.0 (0.64, 1.4)	0.056 (0.047, 0.063)	0.0010 (0.0006, 0.0012)	0.036 (0.023, 0.049)	14% (13%, 16%)
Ethiopia	29 (26, 30)	5.4 (1.2, 14)	0.53 (0.50, 0.64)	0.059 (0.053, 0.064)	0.0011 (0.0007, 0.0012)	0.021 (0.0069, 0.034)	15% (13%, 16%)
Zimbabwe	27 (23, 30)	27 (21, 30)	1.5 (1.0, 2.4)	0.055 (0.043, 0.063)	0.0013 (0.0006, 0.0015)	0.045 (0.032, 0.050)	15% (11%, 17%)
Belarus	12 (8.5, 17)	27 (19, 30)	3.9 (2.6, 5.9)	0.059 (0.046, 0.063)	0.0009 (0.0005, 0.0014)	0.035 (0.013, 0.049)	14% (12%, 17%)
Republic of Moldova	20 (15, 28)	31 (24, 35)	8.1 (6.3, 9.9)	0.054 (0.039, 0.067)	0.0013 (0.0011, 0.0015)	0.039 (0.021, 0.050)	16% (13%, 17%)
Azerbaijan	20 (14, 27)	27 (23, 30)	3.0 (1.0, 5.7)	0.054 (0.044, 0.062)	0.0013 (0.0007, 0.0015)	0.036 (0.011, 0.049)	15% (13%, 17%)

n = 200 posterior samples. Median and 95% credible intervals (brackets) of model estimates are presented. Notations for parameters are listed in Supplementary Table 2. Abbreviations: DS-drug-susceptible, DR-drug-resistant, TB-tuberculosis.

*Calculated by the sum of fractions for fast progression: $\rho_{\mathrm{hi}}^{\mathrm{ad,ne}}/(\rho_{\mathrm{hi}}^{\mathrm{ad,ne}} + d^{\mathrm{ad,ne}} + \upsilon_{\mathrm{rec}})$, and for slow progression: $\left[\upsilon_{\mathrm{rec}}/(\rho_{\mathrm{hi}}^{\mathrm{ad,ne}} + d^{ad,ne} + \upsilon_{\mathrm{rec}})\right]\left[\rho_{\mathrm{lo}}^{\mathrm{ad,ne}}/(\rho_{\mathrm{lo}}^{\mathrm{ad,ne}} + d^{\mathrm{ad,ne}})\right]$. Lifetime risks of HIV-negative adults are presented, to ensure that the model is capturing plausible natural history dynamics.

Supplementary Table 2. Vaccine-averted proportion of RR-TB burden under alternative assumptions.

Scenario	M72-like vaccine				M72-like vaccine and improved RR-TB management			
	Averted proportion of RR-TB cases, %		Averted proportion of RR-TB deaths, %		Averted proportion of RR-TB cases, %		Averted proportion of RR-TB deaths, %	
	Main analysis	Alternative assumption	Main analysis	Alternative assumption	Main analysis	Alternative assumption	Main analysis	Alternative assumption
No catch-up campaigns*	10 (9.7, 11)	1.6 (1.3, 2.1)	7.3 (6.6, 8.1)	1.1 (0.90, 1.5)	14 (12, 16)	5.4 (4.0, 8.0)	31 (29, 33)	27 (24, 29)
Halved protection in HIV +ve*	10 (9.7, 11)	10 (9.5, 11)	7.3 (6.6, 8.1)	7.1 (6.3, 7.9)	14 (12, 16)	13 (12, 16)	31 (29, 33)	31 (29, 33)
Russian Federation	9.4 (8.9, 9.8)	8.6 (8.0, 9.1)	7.3 (6.9, 7.7)	6.6 (6.2, 7.1)	9.5 (8.8, 11)	8.7 (7.9, 9.9)	37 (35, 38)	36 (35, 37)
Nigeria	8.5 (6.7, 10)	8.0 (6.2, 9.5)	7.1 (5.5, 8.4)	6.4 (4.9, 7.7)	10 (7.9, 11)	9.5 (7.5, 11)	8.8 (7.0, 10)	8.2 (6.3, 9.4)
Ukraine	9.5 (9.0, 9.9)	8.8 (8.1, 9.2)	7.3 (6.7, 7.6)	6.6 (6.0, 6.9)	10 (9.2, 12)	9.3 (8.3, 11)	48 (45, 49)	47 (45, 49)

n = 200 posterior samples. Median and 95% credible intervals (brackets) of model estimates are presented. The alternative scenarios include for the removal of catch-up campaigns (top row) and the reduced vaccine protection amongst HIV positive population (second row and beyond)**.** Abbreviations: HIV-human immunodeficiency virus, RR-TB-rifampicin-resistant tuberculosis.

*Global impacts are presented. For the analysis of the reduced vaccine protection among HIV positive population, we show the country-specific effect in the three countries that are incorporated with HIV structure and contributing to largest RR-TB incidence.

Supplementary Table 3. Averted patient-months for second-line treatment in different scenarios.

Country/ Region	Improved RR-TB management		M72-like vaccine		M72-like vaccine and improved RR-TB management	
	Patient-month, thousands	Proportion, %	Patient-month, thousands	Proportion, %	Patient-month, thousands	Proportion, %
India	-3125 (-5396, -1736)	-12 (-13, -9.7)	1676 (958, 3603)	6.4 (4.9, 7.9)	-1158 (-2404, -421)	-4.7 (-6.8, -1.7)
Indonesia	-846 (-1233, -540)	-18 (-20, -14)	357 (240, 538)	7.4 (6.5, 8.3)	-429 (-642, -238)	-9.0 (-11, -6.1)
Myanmar	0	0	128 (74, 207)	6.3 (5.0, 7.6)	128 (74, 207)	6.3 (5.0, 7.6)
Bangladesh	-37 (-69, -15)	-2.6 (-3.6, -1.5)	110 (52, 196)	7.4 (6.3, 8.3)	72 (35, 142)	5.1 (3.7, 6.1)
DPR Korea	0.74 (-32, 240)	0.07 (-3.3, 11)	100 (46, 300)	8.4 (7.0, 11)	97 (35, 455)	8.0 (4.5, 19)
Thailand	-60 (-98, -29)	-7.6 (-8.6, -5.7)	56 (21, 100)	7.0 (5.8, 7.9)	-0.17 (-12, 22)	-0.02 (-1.7, 2.0)
**South-East Asia Region**	-4053 (-6288, -2623)	-11 (-12, -9.2)	2453 (1713, 4430)	6.7 (5.6, 7.7)	-1281 (-2440, -530)	-3.5 (-5.4, -1.4)
China	229 (-105, 2531)	1.1 (-0.59, 9.5)	1434 (925, 2217)	6.7 (6.1, 8.3)	1662 (876, 4189)	7.6 (5.7, 16)
Philippines	-392 (-660, -180)	-13 (-18, -9.4)	172 (78, 305)	5.7 (4.5, 7.2)	-181 (-330, -81)	-6.5 (-9.8, -3.5)
Viet Nam	-21 (-34, -6.3)	-0.87 (-1.1, -0.24)	221 (92, 497)	8.9 (6.0, 12)	203 (76, 477)	8.0 (4.9, 11)
Papua New Guinea	-25 (-54, 31)	-6.3 (-10, 4.8)	45 (23, 102)	9.8 (7.3, 13)	14 (-5.5, 100)	3.3 (-1.9, 15)
**West Pacific Region**	-178 (-667, 2200)	-0.63 (-2.5, 7.2)	1901 (1248, 2721)	7.0 (6.3, 8.3)	1686 (881, 4314)	6.2 (4.2, 14)
Russian Federation	-174 (-241, -102)	-1.8 (-2.1, -1.1)	684 (519, 908)	7.0 (6.6, 7.5)	525 (404, 723)	5.4 (4.9, 6.3)
Ukraine	27 (-0.65, 92)	0.76 (-0.01, 2.3)	252 (159, 360)	7.0 (6.5, 7.4)	278 (173, 402)	7.7 (6.9, 9)
Kazakhstan	0	0	101 (61, 151)	8.4 (7.6, 9.7)	101 (61, 151)	8.4 (7.6, 9.7)
Uzbekistan	-44 (-68, -28)	-4.2 (-4.7, -3.1)	77 (48, 117)	7.2 (6.1, 8.4)	36 (18, 63)	3.4 (2.2, 4.8)
Kyrgyzstan	20 (10, 30)	2.5 (1.5, 3.5)	73 (55, 96)	9.2 (8.6, 9.6)	89 (63, 118)	11 (10, 12)
Tajikistan	-13 (-22, -9.0)	-2.4 (-2.7, -1.7)	62 (33, 116)	11 (8.7, 12)	49 (25, 96)	8.8 (6.4, 10)
Belarus	7.0 (3.6, 13)	1.3 (0.89, 2.0)	39 (24, 63)	7.7 (6.9, 8.8)	45 (28, 72)	8.8 (7.7, 10)
Republic of Moldova	0.08 (-1.9, 4.9)	0.02 (-0.47, 1.0)	33 (24, 44)	7.7 (7.2, 8.2)	33 (23, 46)	7.7 (6.8, 8.9)
Azerbaijan	4.3 (1.6, 13)	1.1 (0.52, 2.6)	29 (18, 46)	7.3 (6.3, 9.2)	33 (20, 57)	8.3 (6.9, 11)
**European Region**	-169 (-250, -84)	-0.92 (-1.2, -0.51)	1378 (1169, 1576)	7.4 (7.1, 7.7)	1208 (1039, 1397)	6.5 (6.1, 7.1)
Pakistan	-315 (-515, -177)	-5.2 (-6.8, -3.0)	344 (212, 567)	5.7 (4.7, 6.2)	42 (-55, 192)	0.70 (-0.85, 2.8)
Somalia	-1.2 (-3.4, 0.96)	-0.21 (-0.36, 0.08)	40 (13, 109)	6.0 (3.8, 8.4)	39 (13, 106)	5.9 (3.6, 8.3)
**Eastern Mediterranean Region**	-317 (-516, -178)	-4.7 (-6.0, -2.7)	388 (244, 631)	5.7 (4.8, 6.3)	85 (-23, 260)	1.3 (-0.33, 3.3)
Nigeria	-52 (-87, -31)	-2.4 (-2.9, -2.0)	112 (65, 193)	5.3 (4.0, 6.3)	65 (33, 118)	3.1 (1.8, 4.1)
South Africa	-209 (-329, -134)	-12 (-13, -11)	103 (58, 187)	6.1 (4.9, 7.1)	-91 (-152, -58)	-5.5 (-6.8, -3.4)

Mozambique	-73<br>(-196, 237)	-6.1<br>(-11, 10)	52<br>(28, 110)	3.8<br>(2.7, 4.9)	-20<br>(-120, 325)	-1.9<br>(-7.7, 13)
DR Congo	-41<br>(-85, -23)	-3.4<br>(-5.6, -2.5)	56<br>(28, 98)	4.8<br>(3.7, 6.3)	19<br>(-13, 44)	1.6<br>(-1.0, 3.4)
Angola	-124<br>(-382, -68)	-44<br>(-55, -37)	16<br>(6.4, 41)	5.1<br>(3.2, 7.1)	-105<br>(-320, -58)	-36<br>(-47, -30)
Kenya	-13<br>(-23, -7.0)	-3.6<br>(-4.0, -3.2)	19<br>(11, 36)	5.6<br>(4.2, 6.7)	7.9<br>(2.6, 16)	2.2<br>(0.85, 3.5)
Ethiopia	-0.62<br>(-1.2, -0.28)	-0.13<br>(-0.17, -0.08)	35<br>(20, 57)	7.0<br>(5.9, 7.8)	34<br>(20, 56)	6.9<br>(5.8, 7.7)
Zimbabwe	-46<br>(-68, -31)	-17<br>(-18, -17)	20<br>(13, 31)	7.6<br>(6.8, 8.5)	-22<br>(-33, -15)	-8.3<br>(-9.3, -7.5)
**African Region**	-568<br>(-857, -262)	-7.1<br>(-9.8, -2.9)	430<br>(338, 567)	5.3<br>(4.6, 6.0)	-106<br>(-303, 224)	-1.3<br>(-3.6, 2.4)
**Peru/ Region of the America**	22<br>(4.2, 68)	2.3<br>(0.59, 5.8)	75<br>(52, 121)	8.2<br>(7.5, 10)	94<br>(55, 171)	9.8<br>(8.2, 14)
**Total**	-5118<br>(-7612, -2237)	-5.3<br>(-6.7, -2.4)	6690<br>(5510, 8637)	6.8<br>(6.2, 7.3)	1756<br>(328, 4477)	1.8<br>(0.32, 4.5)

n = 200 posterior samples. Median and 95% credible intervals (brackets) of model estimates are presented. Estimates at regional level and global level are highlighted. Abbreviation: RR-TB-rifampicin-resistant tuberculosis.

Supplementary Table 4. Averted health burden attributed to improved RR-TB management alone.

Country/Region	Averted RR-TB cases		Averted RR-TB deaths		Averted SL treatment	
	Number, thousands	Proportion, %	Number, thousands	Proportion, %	Patient-month, thousands	Proportion, %
India	71<br>(29, 207)	3.9<br>(2.0, 7.5)	200<br>(112, 390)	31<br>(28, 34)	-3125<br>(-5396, -1736)	-12<br>(-13, -9.7)
Indonesia	18<br>(6.7, 42)	5.3<br>(2.1, 11)	37<br>(27, 54)	37<br>(35, 39)	-846<br>(-1233, -540)	-18<br>(-20, -14)
Myanmar	0	0	0	0	0	0
Bangladesh	1.7<br>(0.25, 7.6)	2.0<br>(0.30, 6.4)	0.79<br>(0.26, 2.5)	5.1<br>(2.4, 8.7)	-37<br>(-69, -15)	-2.6<br>(-3.6, -1.5)
DPR Korea	8.1<br>(0.38, 49)	11<br>(0.85, 30)	1.8<br>(0.31, 8.4)	17<br>(5.9, 32)	0.74<br>(-32, 240)	0.07<br>(-3.3, 11)
Thailand	1.7<br>(0.16, 5.9)	3.5<br>(0.48, 8.4)	2.9<br>(1.1, 5.7)	20<br>(17, 25)	-60<br>(-98, -29)	-7.6<br>(-8.6, -5.7)
**South-East Asia Region**	111<br>(51, 239)	4.2<br>(2.5, 6.8)	243<br>(160, 441)	30<br>(27, 32)	-4053<br>(-6288, -2623)	-11<br>(-12, -9.2)
China	30<br>(8.8, 176)	3.4<br>(1.2, 15)	34<br>(22, 51)	29<br>(28, 36)	229<br>(-105, 2531)	1.1<br>(-0.59, 9.5)
Philippines	8.8<br>(1.7, 37)	5.6<br>(1.4, 14)	10<br>(6.0, 20)	35<br>(33, 37)	-392<br>(-660, -180)	-13<br>(-18, -9.4)
Viet Nam	0.83<br>(0.16, 3.3)	0.62<br>(0.19, 1.6)	2.6<br>(1.3, 4.9)	11<br>(9.4, 13)	-21<br>(-34, -6.3)	-0.87<br>(-1.1, -0.24)
Papua New Guinea	10<br>(3.2, 32)	23<br>(14, 38)	1.6<br>(0.51, 4.6)	19<br>(12, 32)	-25<br>(-54, 31)	-6.3<br>(-10, 4.8)
**West Pacific Region**	57<br>(23, 203)	4.7<br>(2.2, 13)	50<br>(34, 69)	28<br>(25, 33)	-178<br>(-667, 2200)	-0.63<br>(-2.5, 7.2)
Russian Federation	0.6<br>(-0.84, 4.2)	0.14<br>(-0.16, 1.0)	35<br>(27, 45)	32<br>(31, 33)	-174<br>(-241, -102)	-1.8<br>(-2.1, -1.1)
Ukraine	1.0<br>(-0.23, 4.2)	0.71<br>(-0.15, 2.6)	18<br>(12, 25)	44<br>(41, 46)	27<br>(-0.65, 92)	0.76<br>(-0.01, 2.3)
Kazakhstan	0	0	0	0	0	0
Uzbekistan	0.28<br>(-0.08, 1.1)	0.56<br>(-0.14, 1.9)	3.5<br>(2.4, 5.3)	28<br>(25, 29)	-44<br>(-68, -28)	-4.2<br>(-4.7, -3.1)

Kyrgyzstan	1.4 (0.69, 2.0)	2.5 (2.0, 4.1)	3.0 (2.3, 3.8)	46 (44, 46)	20 (10, 30)	2.5 (1.5, 3.5)
Tajikistan	0.12 (-0.02, 0.51)	0.39 (-0.11, 1.4)	1.6 (0.97, 2.6)	23 (21, 24)	-13 (-22, -9.0)	-2.4 (-2.7, -1.7)
Belarus	0.12 (0.01, 0.37)	0.63 (0.06, 1.8)	0.47 (0.29, 0.74)	16 (13, 19)	7.0 (3.6, 13)	1.3 (0.89, 2.0)
Republic of Moldova	0.04 (-0.04, 0.30)	0.26 (-0.27, 1.6)	1.7 (1.3, 2.2)	43 (42, 44)	0.08 (-1.9, 4.9)	0.02 (-0.47, 1.0)
Azerbaijan	0.12 (-0.03, 0.58)	0.74 (-0.21, 2.9)	1.2 (0.77, 1.7)	30 (26, 34)	4.3 (1.6, 13)	1.1 (0.52, 2.6)
**European Region**	4.0 (1.7, 8.1)	0.51 (0.20, 1.0)	65 (54, 76)	33 (32, 35)	-169 (-250, -84)	-0.92 (-1.2, -0.51)
Pakistan	19 (7.8, 40)	4.8 (2.6, 8.3)	19 (12, 31)	19 (18, 20)	-315 (-515, -177)	-5.2 (-6.8, -3.0)
Somalia	0.35 (0.07, 1.1)	0.31 (0.11, 0.82)	0.10 (0.03, 0.28)	0.37 (0.14, 0.84)	-1.2 (-3.4, 0.96)	-0.21 (-0.36, 0.08)
**Eastern Mediterranean Region**	19 (8.2, 40)	3.9 (1.8, 6.7)	19 (12, 31)	15 (11, 18)	-317 (-516, -178)	-4.7 (-6.0, -2.7)
Nigeria	5.5 (2.2, 13)	1.6 (1.1, 2.2)	2.1 (0.96, 4.7)	1.8 (1.4, 2.3)	-52 (-87, -31)	-2.4 (-2.9, -2.0)
South Africa	0.24 (-0.21, 3.3)	0.21 (-0.21, 1.8)	11 (7.1, 19)	26 (22, 29)	-209 (-329, -134)	-12 (-13, -11)
Mozambique	19 (5.4, 90)	12 (5.5, 30)	12 (4.6, 38)	19 (13, 35)	-73 (-196, 237)	-6.1 (-11, 10)
DR Congo	2.6 (0.14, 13)	3.1 (0.29, 8.7)	1.0 (0.3, 3.8)	7.1 (3.9, 12)	-41 (-85, -23)	-3.4 (-5.6, -2.5)
Angola	4.2 (0.74, 26)	12 (3.8, 23)	1.5 (0.64, 6.9)	31 (25, 36)	-124 (-382, -68)	-44 (-55, -37)
Kenya	0.06 (0.00, 0.92)	0.24 (0.01, 2.0)	0.48 (0.26, 0.95)	6.5 (5.2, 7.1)	-13 (-23, -7.0)	-3.6 (-4.0, -3.2)
Ethiopia	0.02 (0.01, 0.11)	0.08 (0.02, 0.33)	0.01 (0.00, 0.03)	0.12 (0.05, 0.32)	-0.62 (-1.2, -0.28)	-0.13 (-0.17, -0.08)
Zimbabwe	-0.08 (-0.13, -0.04)	-0.42 (-0.58, -0.25)	2.1 (1.4, 3.1)	28 (25, 30)	-46 (-68, -31)	-17 (-18, -17)
**African Region**	36 (17, 100)	4.1 (2.3, 11)	32 (21, 59)	12 (8.0, 19)	-568 (-857, -262)	-7.1 (-9.8, -2.9)
Peru/ **Region of the America**	3.3 (1.6, 7.0)	7.5 (4.9, 13)	1.7 (1.0, 2.6)	34 (30, 38)	22 (4.2, 68)	2.3 (0.59, 5.8)
**Total**	245 (153, 424)	3.9 (2.7, 6.6)	416 (320, 610)	26 (23, 28)	-5118 (-7612, -2237)	-5.3 (-6.7, -2.4)

n = 200 posterior samples. Median and 95% credible intervals (brackets) of model estimates are presented. Estimates at regional level and global level are highlighted. Abbreviation: RR-TB-rifampicin-resistant tuberculosis, SL-second-line.

Supplementary Table 5. List of model parameters.

Symbol	Description	Unit	Value (prior range)		Source/note
**Demographics**					
μ	Birth rate	yr-1	(0.01-0.1)		Fitted
$d^{a,h}$	All-cause death rate	yr-1	(fixed)		Weighted average from United Nations World Population Prospect [14]
α	Age group transition rate	yr-1	(1/25-1/5)		Fitted
**HIV epidemiology**					
$\delta^a(t)$	HIV infection rate	yr-1	$a = \mathrm{ch}$	Country-specific (fixed)	Joint United Nations Programme on HIV/AIDS [2]
			$a = \mathrm{ad}$		
$f_{\mathrm{dea,hiv}}$	Increased risk of all-cause death rate due to untreated HIV (relative to HIV -ve)		5.7 (1-10)		Wide ranges to fit to different levels of health care [15]
$q_{\mathrm{dea,art}}$	Reduced risk of all-cause death rate due to ART (relative to untreated HIV)		0.35 (0.29-1)		Lower bound represent best effect in a high-income setting [15]
$\gamma_{\mathrm{art,max}}$	Rate of initiating ART from 2010 and onwards	yr-1	(0.1-12)		Fitted<br>Reflect duration of 1 month - 10 years [16]
g_{ipt}	Proportion of initiating IPT when enrolled for ART		Country-specific (fixed)		Joint United Nations Programme on HIV/AIDS [2]
v_{ipt}	Rate of completing IPT	yr-1	0.41 (fixed)		Reflect duration of 2.43 months [16]
w_{art}	Rate of lost-to-follow-up from ART	yr-1	(Country-specific range)		No larger of the overall dropping out rate for the first 12 months [2]
$q^h_{\mathrm{pgr,art}}$	Reduced risk of TB progression due to ART (relative to untreated HIV)		$h = \mathrm{at, bt}$	0.35 (0.28-0.44)	[17]
			$h = \mathrm{ne, ut}$	1	
$q^{h,s}_{\mathrm{pgr,ipt}}$	Reduced risk of TB progression due to IPT among those receiving ART		$h = \mathrm{at, bt}$<br>$s = \mathrm{ds}$	0.63 (0.41-0.94)	[16], assuming that IPT has no effect on drug-resistant infection
			$h = \mathrm{at, bt}$<br>$s = \mathrm{dr}$	1	
			$h = \mathrm{ne, ut}$	1	
**Natural history of TB**					
β^s_{ini}	Infection rate in 1970 by strain	yr-1	(0-35)		Fitted
k_{beta}	Parameter for geometric decline in β^s_{ini}	yr-1	(0.9-1)		Fitted
$\mathbf{M}_{a,\tilde{a}}$	Age-related standardised contact matrix, for a susceptible person of age a acquiring infection from an infectious case of age $\tilde{a}$		Country-specific (fixed)		[18]
$f^a_{\mathrm{beta,age}}$	Relative infectiousness of active TB by age		$a = \mathrm{ch}$	0.83 (0.66-0.99)	[19]
			$a = \mathrm{ad}$	1	

$f^h_{\text{beta,hiv}}$	Relative infectiousness of active TB by HIV status		$\tilde{h} = \text{ne}$ 1<br>$\tilde{h} \neq \text{ne}$ (0.1-1)	Fitted
c_{1st}	Initial care-seeking rate	yr^{-1}	(0-10)	Fitted<br>Reflect a minimum delay of 1.5 month
c_{2nd}	Care-seeking rate after loss-to-follow-up or treatment failure	yr^{-1}	(6-26)	Fitted<br>Reflect a delay of 2 weeks – 2 months
γ_{tx}	Rate of receiving confirmed diagnosis and initiating anti-TB treatment	yr^{-1}	52 (fixed)	Reflect a delay of 1 week
$g_{\text{tx,max}}$	Proportion of initiating anti-TB treatment from 2010 and onwards		(Country-specific range)	[1]
q_{tx}	Relative coverage of anti-TB treatment prior to 2000 compared to $g_{\text{tx,max}}$		(0.05-1)	Fitted
$q^a_{\text{pgr,age}}$	Relative risk of TB progression by age		$a = \text{ch}$ (0.05-1)<br>$a = \text{ad}$ 1	Assumed to increase with age
$\rho_{i,\text{base}}$	TB progression rates among HIV -ve adults	yr^{-1}	$i = \text{hi}$ .053 (.026 – .079)	[20]
			$i = \text{lo}$ .0010 (.0005-.0015)	[21, 22]
q_{ltbi}	Relative risk of TB infection among latent population		(0.25-0.75)	Fitted
υ_{ltbi}	Stabilisation rate of latent state	yr^{-1}	0.5 (fixed)	Duration of 2 years
υ_{rec}	Stabilisation rate of recovered state	yr^{-1}	0.5 (fixed)	Duration of 2 years
$\upsilon_{\text{tx,fl}}$	Stabilisation rate of first-line treatment	yr^{-1}	2 (fixed)	Duration of 6 months
$\upsilon_{\text{tx,sl}}$	Stabilisation rate of second-line treatment	yr^{-1}	0.5 (fixed)	Duration of 2 years
f^h_{mort}	Increased risk of TB mortality due to HIV		$h \neq \text{ne}$ 1<br>$h = \text{ne}$ 2.56 (1-10)	[23]
$m^{h\neq\text{ut}}$	TB-specific mortality rate among HIV -ve and HIV +ve on ART	yr^{-1}	0.13 (0.08-0.25)	[24]
η	Self-recovery rate for first-line treatment	yr^{-1}	$m^{h\neq\text{ut}}$	50% of untreated TB recovered naturally [24]
g_{dst}	Proportion of active TB cases receiving drug susceptibility test before treatment		(fixed)	Country-specific [1]
$\tau^p_{\text{tx,fl},i}$	Outcome distribution of first-line treatment: success ($i = \text{su}$), failure (fa), default (df), and death (de)		(fixed)	Country-specific [1]
$\tau^p_{\text{tx,sl},i}$	Outcome distribution of second-line treatment: success ($i = \text{su}$), failure (fa), default (df), and death (de)		(fixed)	Country-specific [1]

$g_{\mathrm{tx,sw}}$	Proportion of RR-TB cases transferring to second-line treatment following failure of first-line treatment			0.85 (fixed)	Assumed
σ	Rate of acquiring drug resistance during first-line treatment			(0.005-0.05)	Fitted
φ_i	Relapse rate	yr^{-1}	$i = \mathrm{lo}$	0.032 (0.026-0.038)	[25]
			$i = \mathrm{hi}$	0.14 (0.11-0.17)	
			$i = \mathrm{st}$	.0015 (.0012-.0018)	
**Public and private sectors**					
$\pi_{\mathrm{max}}^{\mathrm{pu}}$	Proportion of active TB cases who seek healthcare in public sector from 2010 and onwards			(0-1)	Fitted to country-specific data
q_{qoc}^{p}	Quality of TB care in private sector, relative to that in public sector		$p = \mathrm{pu}$	1	Assumed
			$p = \mathrm{pr}$	(0.4-0.8)	Affect both diagnosis and treatment through $g_{\mathrm{tx,fl}}^{p}$ and $\tau_{\mathrm{tx,fl},i}^{p}$ in Supplementary Equations (24)-(26)

We retrieved sampling ranges from the 95% confidence intervals if reported in the literature. Wide ranges were assigned to some parameters to capture heterogeneity in TB transmission by country. Notations for the risk structures: ch – children, ad – adults, ds – drug-susceptible, dr – drug-resistant, ne – HIV-negative, ut – HIV-positive and untreated, at – HIV-positive and on ART, bt – HIV-positive and on both ART and IPT. Abbreviations: ART-antiretroviral treatment, HIV-human immunodeficiency virus, IPT-isoniazid preventive therapy, TB-tuberculosis.

Supplementary Table 6. Disease state and risk structure in the model

Type	Notation	Definition
Disease state*	$U^{a,h}$	Uninfected and susceptible to TB
	$L_{\text{hi}}^{a,h,s}$	Latent infection with a risk of fast progression
	$L_{\text{lo}}^{a,h,s}$	Latent infection with a risk of slow progression
	$A^{a,h,s}$	Active TB disease prior to healthcare seeking
	$D^{a,h,s,p}$	Active TB disease waiting for diagnosis in healthcare sectors
	$T_{\text{fl}}^{a,h,s,p}$	On first-line treatment
	$T_{\text{sl}}^{a,h,s,p}$	On second-line treatment
	$E^{a,h,s}$	Active TB disease, temporarily dropped out of care cascade
	$R_{\text{lo}}^{a,h}$	Recovered with a low risk of relapse
	$R_{\text{hi}}^{a,h}$	Recovered from treatment success with a low high of relapse
	$R_{\text{st}}^{a,h}$	Recovered with a stabilised risk of relapse
Risk structure	$a \in \{\text{ch}, \text{ad}\}$	Age groups: children, adults
	$h \in \{\text{ne}, \text{ut}, \text{at}, \text{bt}\}$	HIV states: negative, positive and untreated, on ART, on both ART and IPT
	$s \in \{\text{ds}, \text{dr}\}$	Strains: drug-susceptible, drug-resistant
	$p \in \{\text{pu}, \text{pr}\}$	Healthcare sectors: public, private

Abbreviations: Abbreviations: ART-antiretroviral treatment, HIV-human immunodeficiency virus, IPT-isoniazid preventive therapy, TB-tuberculosis.

*Superscripts of the disease states represent the risk structures incorporated.

Supplementary Table 7. Calibration targets of each country

Country (ISO alpha-3 code)	Demographics		TB burden (per 100,000 population)						HIV burden
	Population sizes, 1970 and 2018*, thousands, (Pop)	Proportion of children population, 2018, %, (PrCh)	All-form TB incidence rates, 2014-2017, (Inc)	Age-specific TB incidence rates, children and adult, 2018, (IncCh, IncAd)	RR-TB incidence rate, 2018, (IncDR)	HIV-TB incidence rates, 2014-2018, (IncHIV)	All-form TB mortality rates, 2014-2018**, (Mort)	All-form TB notification rate in public sectors, 2018, (NtfPu)	HIV prevalence, 2018, per 1,000 population, ($\mathrm{PrevHIV}$)<br>ART coverage, latest available, %, (PrcART)
India (IND)	555190<br>1352642	27	223 (136, 332)<br>217 (137, 315)<br>211 (140, 295)<br>204 (140, 281)	93 (57, 130)<br>238 (144, 331)	9.6 (5.7, 15)	-	37 (35, 40)<br>36 (34, 38)<br>35 (32, 37)<br>34 (32, 36)<br>33 (31, 36)	119	-
China (CHN)	827601<br>1427648	18	67 (57, 77)<br>65 (55, 75)<br>63 (54, 73)<br>62 (53, 72)	14 (12, 16)<br>71 (60, 82)	4.6 (3.5, 6)	-	3.0 (2.7, 3.3)<br>3.0 (2.7, 3.3)<br>3.0 (2.7, 3.3)<br>2.8 (2.5, 3.1)<br>2.8 (2.5, 3.1)	-	-
Russian Federation (RUS)	130149<br>145734	18	70 (45, 100)<br>67 (43, 95)<br>63 (41, 90)<br>59 (38, 84)	8.4 (5.0, 12)<br>64 (38, 89)	28 (18, 40)	9.1 (2.9, 19)<br>8.8 (2.8, 18)<br>13 (8.4, 19)<br>11 (7.3, 16)<br>11 (7.0 ,15)	11 (11, 12)<br>10 (9.6, 11)<br>9.6 (8.7, 11)<br>8.1 (7.2, 9.1)<br>7.2 (6.4, 8.1)	-	Ukraine data<br>Ukraine data
Pakistan (PAK)	58142<br>212228	35	270 (175, 386)<br>270 (180, 378)<br>268 (191, 359)<br>267 (189, 357)	96 (61, 131)<br>357 (229, 485)	13 (8.4, 19)	-	25 (21, 29)<br>23 (19, 28)<br>22 (18, 27)<br>21 (18, 26)<br>21 (17, 25)	119	-
Indonesia (IDN)	114793<br>267671	27	329 (300, 359)<br>325 (297, 355)<br>322 (294, 352)<br>319 (291, 348)	98 (89, 108)<br>394 (356, 433)	8.8 (6.2, 12)	-	42 (39, 45)<br>39 (37, 42)<br>38 (36, 41)<br>38 (35, 40)<br>37 (34, 39)	175	-
Nigeria (NGA)	55982<br>195875	44	219 (143, 311)<br>219 (143, 311)<br>219 (143, 311)<br>219 (143, 311)	87 (48, 127)<br>322 (176, 468)	11 (6.4, 16)	42 (27, 59)<br>37 (24, 53)<br>34 (22, 49)<br>30 (20, 43)<br>27 (17, 38)	86 (58, 120)<br>85 (57, 119)<br>83 (55, 116)<br>81 (53, 115)<br>80 (52, 114)	48	16 (12, 22)<br>53 (40, 71)
Philippines (PHL)	35804<br>106651	31	546 (304, 859)<br>550 (307, 862)<br>554 (311, 866)<br>554 (311, 866)	227 (97, 354)<br>701 (303, 1099)	17 (7.3, 30)	-	29 (25, 33)<br>28 (24, 31)<br>27 (23, 30)<br>26 (22, 29)<br>25 (21, 28)	225	-

Ukraine<br>(UKR)	47089<br>44246	16	94 (61, 135)<br>91 (59, 130)<br>87 (56, 124)<br>84 (54, 119)	19 (11, 26)<br>91 (54, 129)	29 (18, 41)	18 (12, 26)<br>20 (13, 29)<br>18 (12, 26)<br>19 (12, 27)<br>18 (12, 26)	18 (16, 20)<br>17 (15, 19)<br>15 (13, 17)<br>14 (12, 17)<br>13 (11, 15)	-	6.4 (5.9, 6.7)<br>52 (48, 56)
Myanmar<br>(MMR)	27269<br>53708	26	411 (256, 601)<br>391 (248, 567)<br>372 (239, 535)<br>355 (230, 505)	297 (155, 431)<br>354 (187, 518)	21 (14, 30)	-	78 (48, 115)<br>68 (44, 98)<br>61 (40, 87)<br>59 (38, 84)<br>46 (30, 65)	169	-
South Africa<br>(ZAF)	22070<br>57793	29	820 (588, 1090)<br>759 (544, 1010)<br>618 (443, 822)<br>567 (406, 754)	161 (107, 214)<br>669 (444, 891)	19 (12, 28)	499 (358, 664)<br>430 (308, 572)<br>367 (263, 488)<br>338 (242, 449)<br>306 (219, 406)	183 (139, 234)<br>162 (123, 206)<br>144 (110, 181)<br>136 (105, 171)<br>110 (88, 136)	-	183 (168, 195)<br>62 (57, 66)
Viet Nam<br>(VNM)	43405<br>95546	23	205 (129, 300)<br>199 (125, 290)<br>193 (122, 281)<br>188 (119, 272)	37 (22, 54)<br>226 (131, 322)	9.1 (5.7, 13)	-	20 (15, 27)<br>18 (13, 25)<br>16 (11, 22)<br>15 (10, 20)<br>13 (9.1, 18)	-	-
Mozambique<br>(MOZ)	9023<br>29496	45	551 (356, 787)<br>551 (356, 787)<br>551 (356, 787)<br>551 (356, 787)	311 (159, 463)<br>741 (374, 1115)	28 (15, 46)	289 (187, 413)<br>283 (183, 405)<br>243 (158, 348)<br>221 (143, 316)<br>197 (127, 281)	214 (151, 287)<br>215 (152, 288)<br>183 (131, 243)<br>157 (113, 209)<br>145 (104, 193)	-	123 (98, 153)<br>56 (44, 68)
DR Congo<br>(COD)	20011<br>84068	46	325 (210, 464)<br>324 (210, 463)<br>323 (209, 461)<br>322 (208, 460)	93 (52, 134)<br>517 (287, 747)	7.2 (3.6, 12)	48 (15, 98)<br>43 (14, 90)<br>42 (13, 87)<br>32 (21, 46)<br>37 (11, 77)	87 (54, 128)<br>86 (53, 126)<br>81 (50, 118)<br>70 (46, 100)<br>63 (39, 92)	-	8.6 (6.8, 10)<br>57 (47, 67)
Bangladesh<br>(BGD)	64232<br>161377	28	221 (161, 291)<br>221 (161, 291)<br>221 (161, 291)<br>221 (161, 291)	72 (49, 94)<br>279 (189, 368)	3.7 (2, 5.9)	-	46 (30, 65)<br>42 (27, 60)<br>39 (25, 56)<br>34 (22, 49)<br>29 (19, 42)	124	-
DPR Korea<br>(PKR)	14410<br>25550	20	513 (446, 584)<br>513 (446, 584)<br>513 (446, 584)<br>513 (446, 584)	213 (184, 252)<br>589 (500, 672)	20 (10, 34)	-	56 (38, 78)<br>41 (25, 61)<br>43 (26, 63)<br>62 (43, 86)<br>80 (56, 107)	-	-
Kazakhstan<br>(KAZ)	13036<br>18320	28	91 (59 ,130)<br>81 (52 ,115)<br>73 (47 ,105)<br>69 (45 ,99)	6.7 (4.0, 9.4)<br>92 (56, 130)	26 (16, 38)	-	6.3 (5.7, 7.0)**<br>4.8 (4.4, 5.3)**<br>4.4 (4.0, 4.9)**<br>3.8 (3.4, 4.3)**<br>2.6 (2.1, 3.1)**	-	-

Uzbekistan<br>(UZB)	12080<br>32476	29	82 (64 ,103)<br>79 (57 ,105)<br>76 (53 ,103)<br>73 (51 ,99)	34 (21, 46)<br>86 (52, 117)	15 (9.9, 20)	-	7.6 (7.1, 8.1)**<br>8.1 (7.5, 8.8)**<br>7.0 (6.5, 7.5)**<br>6.7 (6.2, 7.2)**<br>6.0 (5.6, 6.5)**	-	-
Somalia<br>(SOM)	3445<br>15008	47	274 (177, 391)<br>274 (177, 391)<br>270 (175, 385)<br>266 (172, 380)	114 (60, 172)<br>387 (200, 574)	27 (15, 42)	-	81 (49, 122)<br>78 (47, 118)<br>77 (46, 117)<br>70 (41, 105)<br>69 (41, 104)	-	-
Thailand<br>(THA)	36885<br>69428	17	167 (102, 247)<br>163 (100, 242)<br>160 (107, 223)<br>156 (119, 199)	24 (19, 31)<br>179 (132, 228)	5.7 (3.3, 8.8)	22 (14, 33)<br>21 (13, 31)<br>18 (12, 25)<br>17 (13, 22)<br>15 (12, 20)	22 (17, 28)<br>22 (17, 28)<br>19 (16, 24)<br>18 (14, 22)<br>17 (13, 20)	113	8.3 (7.3, 10)<br>75 (66, 86)
Angola<br>(AGO)	5890<br>30810	47	370 (240, 529)<br>366 (237, 523)<br>362 (235, 518)<br>359 (232, 512)	125 (67, 180)<br>561 (305, 812)	13 (5.4, 23)	82 (29, 163)<br>78 (27, 156)<br>78 (27, 155)<br>71 (24, 143)<br>34 (22, 49)	96 (58, 142)<br>86 (53, 128)<br>88 (54, 130)<br>96 (58, 142)<br>72 (47, 103)	209	18 (15, 21)<br>27 (23, 31)
Peru<br>(PER)	13460<br>31989	26	125 (95, 158)<br>122 (94, 155)<br>120 (92, 152)<br>119 (91, 150)	30 (23, 39)<br>156 (114, 198)	10 (7.6, 13)	-	8.8 (6.4, 12)<br>9.6 (6.8, 13)<br>8.9 (6.2, 12)<br>8.3 (5.5, 12)<br>8.3 (5.6, 12)	-	-
Kyrgyzstan<br>(KGZ)	2970<br>6304	32	126 (108, 145)<br>136 (116, 157)<br>133 (114, 154)<br>124 (106, 144)	18 (15, 20)<br>162 (136, 188)	47 (39, 57)	-	9.9 (9.6, 10)**<br>8.8 (8.4, 9.1)**<br>8.5 (8.0, 9.0)**<br>7.8 (7.3, 8.3)**<br>6.8 (6.3, 7.3)**	-	-
Kenya<br>(KEN)	11301<br>51393	40	423 (259, 626)<br>380 (233, 564)<br>348 (213, 516)<br>319 (195, 472)	93 (46, 137)<br>423 (216, 633)	4.5 (2.1, 7.9)	150 (92, 223)<br>126 (77, 187)<br>109 (67, 162)<br>91 (56, 135)<br>79 (48, 117)	138 (95, 188)<br>122 (84, 167)<br>110 (76, 151)<br>87 (60, 120)<br>64 (44, 87)	157	45 (39, 55)<br>68 (58, 82)
Papua New<br>Guinea<br>(PNG)	2783<br>8606	36	432 (356, 516)<br>432 (352, 521)<br>432 (352, 521)<br>432 (352, 521)	250 (188, 308)<br>543 (398, 670)	23 (14, 33)	-	53 (34, 76)<br>54 (37, 74)<br>50 (34, 69)<br>58 (40, 79)<br>55 (38, 75)	-	-
Tajikistan<br>(TJK)	2930<br>9101	37	91 (70, 115)<br>86 (66, 109)<br>85 (66, 108)<br>85 (65, 106)	16 (12, 20)<br>123 (90, 155)	20 (15, 26)	-	10 (9.1, 11)<br>9.6 (8.7, 10)<br>9.4 (8.5, 10)<br>9.2 (8.3, 10)<br>9.0 (8.1, 9.9)	-	-

Ethiopia<br>(ETH)	28415<br>109224	41	207 (134, 295)<br>192 (142, 250)<br>177 (125, 239)<br>164 (115, 221)	43 (27, 61)<br>226 (144, 306)	1.4 (0.96, 2.0)	-	44 (28, 62)<br>31 (21, 42)<br>30 (20, 41)<br>28 (19, 39)<br>24 (16, 34)	93	-
Zimbabwe<br>(ZWE)	5289<br>14439	42	278 (202, 366)<br>242 (179, 314)<br>233 (173, 302)<br>221 (164, 287)	39 (28, 52)<br>337 (228, 433)	10 (7.4, 14)	188 (137, 248)<br>168 (125, 218)<br>151 (112, 196)<br>140 (103, 181)<br>130 (96, 169)	52 (39, 67)<br>43 (32, 55)<br>38 (28, 50)<br>35 (26, 46)<br>32 (23, 41)	-	144 (132, 168)<br>88 (77, 97)
Belarus<br>(BLR)	8914<br>9453	17	51 (39, 65)<br>50 (38, 63)<br>42 (33, 54)<br>37 (28, 47)	4.8 (3.6, 6.1)<br>37 (27, 46)	14 (11, 18)	-	6.1 (5.7, 6.6)**<br>6.0 (5.6, 6.4)**<br>6.2 (5.7, 6.7)**<br>6.0 (5.6, 6.4)**<br>5.9 (5.5, 6.3)**	-	-
Republic of<br>Moldova<br>(MDA)	3596<br>4052	16	115 (98, 132)<br>102 (87, 118)<br>101 (86, 117)<br>95 (81, 110)	17 (14, 20)<br>100 (82, 114)	34 (28, 40)	-	11 (9.9, 12)**<br>9.6 (8.7, 11)**<br>8.8 (7.9, 9.7)**<br>7.5 (6.8, 8.3)**<br>6.3 (5.6, 7.1)**	-	-
Azerbaijan<br>(AZE)	5180<br>9950	23	76 (58, 96)<br>71 (54, 90)<br>66 (51, 84)<br>66 (51, 84)	21 (15, 26)<br>76 (56, 97)	13 (9.5, 16)	-	4.9 (4.5, 5.2)**<br>6.1 (5.7, 6.7)**<br>7.6 (7.1, 8.1)**<br>8.9 (8.4, 9.4)**<br>10 (9.8, 11)**	-	-

Underlined text indicates the notations for calibration targets used in the joint log-likelihood function, as defined in Supplementary Equation (37). Uncertainty ranges of calibration targets shown in brackets are provided by their original data sources [1, 2, 14], or a +-20% range of point estimates is assumed. Abbreviations: ART-antiretroviral treatment, HIV-human immunodeficiency virus, IPT-isoniazid preventive therapy, HIV-human immunodeficiency virus, RR-TB-rifampicin-resistant tuberculosis.

* Relative growth of population from 1970 to 2018 is used as a calibration target in the joint log-likelihood function.

** A +-50% range of point estimates was used, to address wider uncertainty from the fluctuated notifications reported in the World Health Organization database.

Supplementary Table 8. Source of country-specific data used in this study.

Dataset	Extracted information	Source
World Prospect Population	Country data on birth, death, life expectancy, age distribution of population	United Nations [14]<br>http://population.un.org/wpp/
Global TB database	Country data on TB incidence (age- and strain-specific), mortality, notification, and anti-TB treatment outcomes (first- and second-line)	World Health Organization [1]<br>http://www.who.int/tb/data/en/
AIDSinfo	Country estimates of HIV incidence, prevalence, and antiretroviral treatment uptake	Joint United Nations Programme on HIV/AIDS [26]<br>http://aidsinfo.unaids.org/
Public-private-mix priority list	Country list with priority of engagement in private care for TB patients	World Health Organization<br>Roadmap for public-private mix [27]
Demographic and health survey	Country data on antibiotic use related to acute respiratory infection among under-five-year-olds	United States Agency for International Development [28]<br>http://dhsprogram.com/data/<br>Extracted with “rdhs” package (v.0.6.3) through R [29]
Global burden of disease 2017	Country data on burden of cough-related respiratory diseases	Institute of Health Metric Evaluation [30]<br>http://ghdx.healthdata.org/gbd-2017

Abbreviations: HIV-human immunodeficiency virus, TB-tuberculosis.

Supplementary Methods 1. Model structure and equations.

In each of the 30 countries, we developed a compartmental model to capture the dynamics of tuberculosis (TB) transmission, using ordinary differential equations representing distinct disease states (Supplementary Table 6) and their transitions along the natural history of TB (Supplementary Fig. 3). Parameters involved in the model are listed in Supplementary Table 5. Two age groups – children (<15 years old) and adults (>=15 years old) – were incorporated into all the country models. According to the categorisation mentioned in the main text, we further included the risk structures of HIV epidemiology or/and public and private sectors to characterise the key heterogeneities in TB burden.

In the following sections, we first describe the key structures for demographics and infection of human immunodeficiency virus (HIV), and then demonstrate the transmission, natural history and healthcare seeking pathway of TB (Supplementary Fig. 15).

Demographics

In each of the country models, the total population given time t are distributed into mutually exclusive disease states:

$$N(t) = \sum_{a,h,s,p} \begin{pmatrix} U^{a,h}(t) + L_{\text{hi}}^{a,h,s}(t) + L_{\text{lo}}^{a,h,s}(t) + A^{a,h,s}(t) + D^{a,h,s,p}(t) + \\ T_{\text{fl}}^{a,h,s,p}(t) + T_{\text{sl}}^{a,h,s,p}(t) + E^{a,h,s}(t) + R_{\text{lo}}^{a,h}(t) + R_{\text{hi}}^{a,h}(t) + R_{\text{st}}^{a,h}(t) \end{pmatrix} \quad (1)$$

We modelled the demographic trend over 1970-2018 by birth, death, and aging in the population. We summarised the changes in demographics with the Supplementary Equations (2) and (3), where $\mathrm{Y} \in \{U, L_{\text{hi}}, L_{\text{lo}}, A, D, E, R_{\text{lo}}, R_{\text{hi}}, R_{\text{st}}\}$.

$$\Delta Y_{\text{dmg}}^{\text{ch},h,s,p}(t) = \underbrace{I_{Y=U^{\text{ch,ne}}}\, \mu N(t)}_{\text{birth}} - \underbrace{I_{Y\notin\{T_{\text{fl}},T_{\text{sl}}\}} d^{\text{ch},h} Y^{\text{ch},h,s,p}(t)}_{\text{death}} - \underbrace{\alpha Y^{\text{ch},h,s,p}(t)}_{\text{ageing}} \quad (2)$$

$$\Delta Y_{\text{dmg}}^{\text{ad},h,s,p}(t) = \underbrace{\alpha Y^{\text{ch},h,s,p}(t)}_{\text{ageing}} - \underbrace{I_{Y\notin\{T_{\text{fl}},T_{\text{sl}}\}} d^{\text{ad},h} Y^{\text{ad},h,s,p}(t)}_{\text{death}} \quad (3)$$

For a disease state $Y^{a,h,s,p}$, μ denotes birth rate; $d^{a,h}$ denotes death rates specific to an age group a with the HIV state h; and α denotes the transition rate from the child to adult groups. As specified by the indicator parameter $I_{Y=U^{\text{ch,ne}}}$, all neonates are not infected with either TB or HIV. The average death rates in children and adults were obtained from the country-specific estimates of World Population Prospects over 2015-2020 [14], and then back calculated to the HIV-specific death rates by prevalence and ART coverage in 2018 [2]. Using the indicator parameter $I_{Y\notin\{T_{\text{fl}},T_{\text{sl}}\}}$, we excluded these background death rates for patients undergoing first-line and second-line treatment, because mortality during treatment are captured by the cohort data for treatment outcome distribution. In each country, the birth rate and ageing rate were calibrated to the total population sizes in 1970 and 2018, and the proportion of child population among the total population in 2018. The demographic changes, represented by, are added to the differential equation of each state.

Transition of HIV states

In countries where the proportion of HIV infection among incident TB cases exceeds 10%, a risk structure was applied to further divide the populations by HIV state h – negative (ne), untreated (ut), on antiretroviral treatment (ART) (at) on both ART and isoniazid preventive therapy (IPT) (bt). The transitions between these HIV states are structured as Supplementary Equations (4)-(7), where $Y^{a,h,s,p}$ represents a specific state over the development of TB (Supplementary Fig. 15c).

$$\Delta Y_{\mathrm{hiv}}^{a,\mathrm{ne},s,p}(t) = -\underbrace{\delta^{a}(t)Y^{a,\mathrm{ne},s,p}(t)}_{\text{HIV infection}} \tag{4}$$

$$\Delta Y_{\mathrm{hiv}}^{a,\mathrm{ut},s,p}(t) = \underbrace{\delta^{a}(t)Y^{a,\mathrm{ne},s,p}(t)}_{\text{HIV infection}} - \underbrace{\gamma_{\mathrm{art}}(t)Y^{a,\mathrm{ut},s,p}(t)}_{\text{initiating ART}} + \underbrace{w_{\mathrm{art}}\left(Y^{a,\mathrm{at},s,p}(t) + Y^{a,\mathrm{bt},s,p}(t)\right)}_{\text{ART default}} \tag{5}$$

$$\Delta Y_{\mathrm{hiv}}^{a,\mathrm{at},s,p}(t) = \underbrace{\left(1 - g_{\mathrm{ipt}}\right)\gamma_{\mathrm{art}}(t)Y^{a,\mathrm{ut},s,p}(t)}_{\text{initiating ART only}} + \underbrace{v_{ipt}Y^{a,\mathrm{bt},s,p}(t)}_{\text{completing IPT}} - \underbrace{w_{\mathrm{art}}Y^{a,\mathrm{at},s,p}(t)}_{\text{ART default}} \tag{6}$$

$$\Delta Y_{\mathrm{hiv}}^{a,\mathrm{bt},s,p}(t) = \underbrace{g_{\mathrm{ipt}}\gamma_{\mathrm{art}}(t)Y^{a,\mathrm{ut},s,p}(t)}_{\text{initiating ART and IPT}} - \underbrace{v_{\mathrm{ipt}}Y^{a,\mathrm{bt},s,p}(t)}_{\text{completing IPT}} - \underbrace{w_{\mathrm{art}}Y^{a,\mathrm{bt},s,p}(t)}_{\text{ART default}} \tag{7}$$

For simplicity, we did not model the dynamic transmission of HIV but assumed age-specific infection rates $\delta^{a}(t)$ over 1990-2018, with data input from Joint United Nations Programme on HIV/AIDS [2]. At such rates the uninfected population progress to the state of untreated HIV. In addition, as shown in equation (8), the population of untreated HIV initiates ART with a rate $\gamma_{\mathrm{art}}(t)$, increasing linearly from 2000 to 2010, and from 2010 onwards staying at the plateau value. On initiation of ART, a proportion g_{ipt} of patients simultaneously begin IPT and then complete the treatment course at a rate v_{ipt}. All the HIV-infected patients on ART may not continue the treatment at a dropping rate w_{art}.

$$\gamma_{\mathrm{art}}(t) = \begin{cases} 0 & , \quad t < 2000 \\ \dfrac{(t-2000)}{(2010-2000)}\gamma_{\mathrm{art,max}}, & 2000 \le t < 2010 \\ \gamma_{\mathrm{art,max}} & , \quad t \ge 2010 \end{cases} \tag{8}$$

HIV infection weakens the immune system of patients and affects the general death rates. The impact of HIV is likely to vary by the healthcare system and coexisting health burden in each country. In this study, we specified the effect of HIV state on all-cause death rates $d^{a,h}$ as:

$$d^{a,h} = \begin{cases} d^{a,\mathrm{ne}} & , \quad h = \mathrm{ne} \\ d^{a,\mathrm{ne}}f_{\mathrm{dea,hiv}} & , \quad h = \mathrm{ut} \\ d^{a,\mathrm{ne}}f_{\mathrm{dea,hiv}}q_{\mathrm{dea,art}}, & h \in \{\mathrm{at},\mathrm{bt}\} \end{cases}, \tag{9}$$

where $f_{\mathrm{dea,hiv}}$ denotes the increased risks of untreated HIV compared to the uninfected population, and $q_{\mathrm{dea,art}}$ denotes the effect of ART in reducing mortality rate. As a long-term follow-up study suggested the all-cause mortality remained higher among HIV-infected population compared to the general population, even with widely available HIV care [15], the mortality rates in different HIV

states are constrained as: $d^{a,\mathrm{ne}} \leq d^{a,h\in\{\mathrm{at,bt}\}} \leq d^{a,\mathrm{ut}}$. Death rates for HIV on ART are not different by whether IPT is initiated at the same time.

Dynamic transmission of TB

We defined infectious cases $X^{a,h,s,p}(t)$ as those with active TB but without effective treatment, including TB patients who do not initiate healthcare seeking, who are in healthcare system but do not initiate treatment, who fail after completing full-course treatment, who default on second-line treatment, and rifampicin-resistant (RR)-TB cases who undergo ineffective first-line treatment:

$$X^{a,h,s,p}(t) = A^{a,h,s,p}(t) + D^{a,h,s,p}(t) + E^{a,h,s,p}(t) + I_{s=\mathrm{dr}} T_{\mathrm{fl}}^{a,h,s,p}(t), \quad (10)$$

where $I_{s=dr}$ is an indicator factor to select drug-resistant TB cases on first-line treatment.

The age-specific and strain-specific force of infection is constructed, as:

$$\lambda^{a,\tilde{s}}(t) = \beta^{\tilde{s}}(t)\mathbf{M}_{a,\tilde{a}} \sum_{\tilde{a},\tilde{h},p} f_{\mathrm{beta,age}}^{\tilde{a}} f_{\mathrm{beta,hiv}}^{\tilde{h}} \frac{X^{\tilde{a},\tilde{h},\tilde{s},p}(t)}{N^{\tilde{a}}(t)} \quad (11)$$

$$\beta^{\mathrm{ds}}(t) = \begin{cases} \beta_{\mathrm{ini}}^{\mathrm{ds}} & , \quad t < 1970 \\ \beta_{\mathrm{ini}}^{\mathrm{ds}} {k_{\mathrm{beta}}}^{(t-1970)}, & t \geq 1970 \end{cases} \quad (12)$$

$$\beta^{\mathrm{dr}}(t) = \begin{cases} 0 & , \quad t < 1970 \\ \beta_{\mathrm{ini}}^{\mathrm{dr}} {k_{\mathrm{beta}}}^{(t-1970)}, & t \geq 1970' \end{cases} \quad (13)$$

where $\beta^{\tilde{s}}(t)$ denotes the infections per year, by an infectious case having drug susceptibility status $\tilde{s}$; $f_{\mathrm{beta,age}}^{\tilde{a}}$ denotes the reduced infectiousness due to higher proportion of extrapulmonary TB among the total cases; $f_{\mathrm{beta,hiv}}^{\tilde{h}}$ denotes the reduced infectiousness potentially caused by deficient immune system and decreased mobility; $\mathbf{M}_{a,\tilde{a}}$ is a 2-by-2 matrix that represents the standardised mixing pattern between a susceptible person of age group a and an infectious case of age group $\tilde{a}$ [18]. The force of infection is proportional to the prevalence of strain- and age-specific prevalence of infectious TB cases, denoted as $\frac{\sum_{\tilde{h},p} X^{\tilde{a},\tilde{h},\tilde{s},p}(t)}{N^{\tilde{a}}(t)}$. We assumed drug resistance of TB disease to emerge recently in 1970, as a result of inappropriate use of antimicrobial drugs and its consequent transmission [31]. To address the improvement of general living standards and nutritional condition, we applied a geometric decline from 1970 to the TB infection rate, with a fitted rate parameter k_{beta}.

Development and transition of TB states

The following equations show transitions over time for each disease state listed in Supplementary Table 5. For each state, the transitions of demographics and HIV states are added to the end of each equation, through the summary terms, $\Delta Y_{\mathrm{dmg}}^{a,h,s,p}(t)$ and $\Delta Y_{\mathrm{hiv}}^{a,h,s,p}(t)$, respectively.

- Uninfected ($U^{a,h}$)

The susceptible population, $U^{a,h}$, can be infected by drug-susceptible- (DS-) or RR-TB depending on the force of infection, $\lambda^{a,s}(t)$.

$$\frac{dU^{a,h}(t)}{dt} = -\underbrace{\lambda^{a,\tilde{s}}(t)U^{a,h}(t)}_{\text{TB infection}} + \underbrace{\Delta U^{a,h}_{\mathrm{dmg}}(t)}_{\substack{\text{demographic}\\ \text{change}}} + \underbrace{\Delta U^{a,h}_{\mathrm{hiv}}(t)}_{\substack{\text{HIV state}\\ \text{transition}}} \tag{14}$$

- Latent infection with a risk of fast and slow TB progression ($L^{a,h,s}_{\mathrm{hi}}$ and $L^{a,h,s}_{\mathrm{lo}}$)

Our model structure for the risk of active disease soon after infection, as well as the dynamics of latent infection thereafter, is consistent with a recent study assessing appropriate model design for these factors [32]. In particular, we assumed that individuals with latent TB infection are separated into two groups, $L^{a,h,s}_{\mathrm{hi}}$ and $L^{a,h,s}_{\mathrm{lo}}$. The former group represents the latent population who acquires infection recently, less than two years, and experiences a higher rate of disease progression; following two and more years of infection, the latent population transitions to the latter group and they have a comparatively small rate of disease progression. Reinfection is possible for all the latent population, including those who have recovered from the disease, but the force of infection rate is reduced by q_{β} due to protection from previous infection.

$$\begin{aligned}\frac{dL^{a,h,s}_{\mathrm{hi}}(t)}{dt} = {} & \underbrace{\lambda^{a,\tilde{s}}(t)U^{a,h}(t)}_{\text{TB infection}} + \underbrace{\lambda^{a,\tilde{s}}(t)q_{\mathrm{ltbi}}\left(L^{a,h,s}_{\mathrm{hi}}(t) + L^{a,h,s}_{\mathrm{lo}}(t) + R^{a,h}_{\mathrm{hi}}(t) + R^{a,h}_{\mathrm{lo}}(t) + R^{a,h}_{\mathrm{st}}(t)\right)}_{\text{TB reinfection}} \\ & - \underbrace{v^{a.h}_{\mathrm{ltbi}}L^{a,h,s}_{\mathrm{hi}}(t)}_{\text{stabalisation}} - \underbrace{\lambda^{a,\tilde{s}}(t)q_{\mathrm{ltbi}}L^{a,h,s}_{\mathrm{hi}}(t)}_{\text{TB reinfection}} - \underbrace{\rho^{a.h,s}_{\mathrm{hi}}L^{a,h,s}_{\mathrm{hi}}(t)}_{\substack{\text{TB progression}\\ \text{(fast)}}} + \underbrace{\Delta L_{\mathrm{hi}}{}^{a,h,s}_{\mathrm{dmg}}(t)}_{\substack{\text{demographic}\\ \text{change}}} \\ & + \underbrace{\Delta L_{\mathrm{hi}}{}^{a,h,s}_{\mathrm{hiv}}(t)}_{\substack{\text{HIV state}\\ \text{transition}}}\end{aligned} \tag{15}$$

$$\frac{dL^{a,h,s}_{\mathrm{lo}}(t)}{dt} = \underbrace{v^{a.h}_{\mathrm{ltbi}}L^{a,h,s}_{\mathrm{hi}}(t)}_{\text{stabalisation}} - \underbrace{\rho^{a.h,s}_{\mathrm{lo}}L^{a,h,s}_{\mathrm{lo}}(t)}_{\substack{\text{TB progression}\\ \text{(slow)}}} - \underbrace{\lambda^{a,\tilde{s}}(t)q_{\mathrm{ltbi}}L^{a,h,s}_{\mathrm{lo}}(t)}_{\text{TB reinfection}} + \underbrace{\Delta L_{\mathrm{lo}}{}^{a,h,s}_{\mathrm{dmg}}(t)}_{\substack{\text{demographic}\\ \text{change}}} + \underbrace{\Delta L_{\mathrm{lo}}{}^{a,h,s}_{\mathrm{hiv}}(t)}_{\substack{\text{HIV state}\\ \text{transition}}} \tag{16}$$

As defined in the Supplementary Equations (17) and (18), the TB primary progression and reactivation rates, $\rho^{a.h}_{\mathrm{hi}}$ and $\rho^{a.h}_{\mathrm{lo}}$, respectively, are specific to age and HIV status. Children show a reduced risk of TB progression, $q^{\mathrm{ch}}_{\mathrm{pgr,age}}$, compared to adults, while individuals with HIV infection have an increased risk, $f^{h\neq\mathrm{ne}}_{\mathrm{pgr,hiv}}$. In addition, among the HIV-infected population, the TB progression rate declines as they receive proper care and treatment; we modelled the protection of ART by $q^{h}_{\mathrm{pgr,art}}$, and the multiplicative protection of IPT to ART by $q^{h,s}_{\mathrm{pgr,ipt}}$. IPT is assumed to prevent DS-TB disease only.

$$\rho_{\text{hi}}^{a.h,s} = \rho_{\text{hi,base}} q_{\text{pgr,age}}^{a} f_{\text{pgr,hiv}}^{h} q_{\rho\text{gr,art}}^{h} q_{\rho\text{gr,ipt}}^{h,s} \tag{17}$$

$$\rho_{lo}^{a.h,s} = \rho_{\text{lo,base}} q_{\text{pgr,age}}^{a} f_{\text{pgr,hiv}}^{h} q_{\rho\text{gr,art}}^{h} q_{\rho\text{gr,ipt}}^{h,s} \tag{18}$$

- Active TB disease prior to healthcare seeking ($A^{a,h,s}$)

After disease progression from latent infection or relapse, individuals enter the state of active disease, $A^{a,h,s}$ and become infectious before self-recovery or effective treatment. In this model, we did not consider extrapulmonary TB cases, which are not infectious and more common in children. Nevertheless, we addressed this age-related heterogeneity in infectiousness when constructing the force of TB infection, as described in Supplementary Equations (11)-(13).

$$\frac{dA^{a,h,s}(t)}{dt} = \underbrace{\rho_{\text{hi}}^{a.h,s} L_{\text{hi}}^{a,h,s}(t)}_{\substack{\text{TB progression} \\ \text{(fast)}}} + \underbrace{\rho_{\text{lo}}^{a.h,s} L_{\text{lo}}^{a,h,s}(t)}_{\substack{\text{TB progression} \\ \text{(slow)}}} + \underbrace{\varphi_{\text{lo}} R_{\text{lo}}^{a,h}(t)}_{\substack{\text{relapse} \\ \text{(low risk)}}} + \underbrace{\varphi_{\text{hi}} R_{\text{hi}}^{a,h}(t)}_{\substack{\text{relapse} \\ \text{(high risk)}}} + \underbrace{\varphi_{\text{st}} R_{\text{st}}^{a,h}(t)}_{\substack{\text{relapse} \\ \text{(stable risk)}}} - \underbrace{c_{1\text{st}} A^{a,h,s}(t)}_{\substack{\text{inital} \\ \text{care−seeking}}} - \underbrace{m^h A^{a,h,s}(t)}_{\substack{\text{TB−specific} \\ \text{mortality}}} - \underbrace{\eta A^{a,h,s}(t)}_{\substack{\text{self−} \\ \text{recovery}}} + \underbrace{\Delta A_{\text{dmg}}^{a,h,s}(t)}_{\substack{\text{demographic} \\ \text{change}}} + \underbrace{\Delta A_{\text{hiv}}^{a,h,s}(t)}_{\substack{\text{HIV state} \\ \text{transition}}} \tag{19}$$

Patients with active TB bear an excess disease-specific mortality rate, m^h, which was assumed to be higher in untreated HIV by $f_{\text{mort}}^{h=\text{ut}}$, compared to those without HIV infection or on ART.

$$m^h = m^{h \neq \text{ut}} \times f_{\text{mort}}^{h} \tag{20}$$

- Active TB disease in healthcare sectors waiting for diagnosis ($D^{a,h,s,p}$)

As Supplementary Fig. 3b shown, we denote active TB cases who seek healthcare in public and private sectors as $D^{a,h,s,p}$. In this state, active TB cases may receive anti-TB treatment after diagnosis.

$$\frac{dD^{a,h,s,p}(t)}{dt} = \underbrace{c_{1\text{st}} \pi^p(t) A^{a,h,s}(t)}_{\substack{\text{inital} \\ \text{care−seeking}}} + \underbrace{c_{2\text{nd}} \pi^p(t) E^{a,h,s}(t)}_{\substack{\text{secondary} \\ \text{care−seeking}}} - \underbrace{\gamma_{tx} D^{a,h,s,p}(t)}_{\substack{\text{diagnosis and} \\ \text{treatment} \\ \text{initiation}}} - \underbrace{\eta D^{a,h,s,p}(t)}_{\substack{\text{self−} \\ \text{recovery}}} + \underbrace{\Delta D_{\text{dmg}}^{a,h,s,p}(t)}_{\substack{\text{demographic} \\ \text{change}}} + \underbrace{\Delta D_{\text{hiv}}^{a,h,s,p}(t)}_{\substack{\text{HIV state} \\ \text{transition}}} \tag{21}$$

We modelled the time-varying proportions of healthcare seeking in public and private sectors by $\pi^p(t)$. Prior to 2000, we assumed there are no TB patients seeking healthcare in public sectors, which specifically refer to those provide standardised DOTS programme. During 2000-2010, the proportion of TB patients seek healthcare in public sectors, $\pi^{\text{pu}}(t)$, linearly increases and maintains at the plateau level from 2010 onwards. This increase reflects the adoption of DOTS and its resulting improvement in quality of TB care.

$$\pi^{\mathrm{pu}}(t)=\begin{cases}0 & , \ t<2000 \\ \dfrac{(t-2000)}{(2010-2000)}\pi_{\max}^{\mathrm{pu}}, & 2000\le t<2010 \\ \pi_{\max}^{\mathrm{pu}} & , \ t\ge 2010\end{cases} \tag{22}$$

$$\pi^{\mathrm{pr}}(t)=1-\pi^{\mathrm{pu}}(t) \tag{23}$$

Note that for countries without the risk structure of public and private sectors, all patients visit and initiate treatment in public sectors for the whole simulation period ($\pi^{\mathrm{pu}}(t)=1$). Instead, we modelled the improvement of TB services by increasing the treatment initiation over 2000-2010, with details described in the next section.

- TB patients on first- and second-line treatment ($T_{\mathrm{fl}}^{a,h,s,p}$ and $T_{\mathrm{sl}}^{a,h,s,p}$), and lost-to-follow-up during healthcare seeking ($E^{a,h,s}$)

To capture the transmission dynamics of DS- and RR-TB in each country, we included the model structures to characterise healthcare seeking process and treatment outcomes. we obtained country-specific proportions of patients initiating anti-TB treatment and distributions of treatment outcomes from the World Health Organization (WHO) Global TB database [1]. These data were assumed to reflect the situation in public sectors, while access and quality of care provided by private sectors was further discounted. In particular, the proportions of active TB patients who initiate healthcare seeking are later put on first- and second-line treatment are respectively denoted in Supplementary Equations (24) and (25).

$$g_{\mathrm{tx,fl}}^{p}=q_{\mathrm{qoc}}^{p}g_{\mathrm{tx,max}} \tag{24}$$

$$g_{\mathrm{tx,,sl}}^{p}=\begin{cases}g_{\mathrm{tx,max}} & , \quad p=\mathrm{pu} \\ 0 & , \quad p=\mathrm{pr}\end{cases} \tag{25}$$

As can be seen in the above equations, treatment initiation depends on the capability of providing correct diagnosis and treatment in the country-specific health system [1]. Compared to public sectors, private sectors have an inferior quality of care and thus show a further reduction in initiation of first-line treatment by $q_{\mathrm{qoc}}^{\mathrm{pr}}$. On the other hand, as second-line treatment requires more advanced medical care and cost, we assumed that no RR-TB patients are able to initiate second-line treatment, either through initial healthcare seeking ($g_{\mathrm{tx,sl}}^{\mathrm{pr}}=0$) or switching from first-line treatment (σ).

In addition, four treatment outcomes – success, failure, default, and death – were extracted from the WHO database, for first- and second-line treatment in public sectors. Considering the standard DOTS is less available to patients attend private sectors for treatment, we increased the proportion of default during treatment by $\left(1-q_{\mathrm{qoc}}^{\mathrm{pr}}\right)$ and reduced the proportion of treatment success correspondingly. The distribution of outcomes for first-line treatment in private sectors is thus defined as Supplementary Equation (26).

$$\tau_{\mathrm{tx,fl},i}^{\mathrm{pr}} = \begin{cases} 1 - \tau_{\mathrm{tx},fl,\mathrm{fa}}^{\mathrm{pu}} - \tau_{\mathrm{tx},fl,de}^{\mathrm{pu}} - \tau_{\mathrm{tx},fl,\mathrm{df}}^{\mathrm{pu}} & , \quad i = \mathrm{su} \\ \tau_{\mathrm{tx},fl,\mathrm{fa}}^{\mathrm{pu}} & , \quad i = \mathrm{fa} \\ \left(2 - q_{\mathrm{qoc}}^{\mathrm{pr}}\right)\tau_{\mathrm{tx},fl,\mathrm{df}}^{\mathrm{pu}} & , \quad i = \mathrm{df} \\ \tau_{\mathrm{tx},fl,\mathrm{de}}^{\mathrm{pu}} & , \quad i = \mathrm{de} \end{cases} \tag{26}$$

Next, we calculated the rates of treatment outcomes ($\varepsilon_{\mathrm{tx,fl},i}^{p}$, $\varepsilon_{\mathrm{tx,sl},i}^{p}$) from the proportions ($\tau_{\mathrm{tx,fl},i}^{p}$, $\tau_{\mathrm{tx,sl},i}^{p}$) reported in the WHO database, separately for first- and second-line treatment, and for public and private sectors. During the rate transition, we specified the standard courses for the first- and second-line treatment to be 6 months and 2 years, respectively; reciprocals of these durations, $\upsilon_{\mathrm{tx,fl}}$ and $\upsilon_{\mathrm{tx,sl}}$, were then taken as the rates of completing anti-TB treatment. Outcome of success or failure is observed at the completion of a treatment course.

$$\varepsilon_{\mathrm{tx,fl},i}^{p} = \begin{cases} \upsilon_{\mathrm{tx,fl}}\left(\dfrac{\tau_{\mathrm{tx,fl,su}}^{p}}{\tau_{\mathrm{tx,fl,su}}^{p} + \tau_{\mathrm{tx,fl,fa}}^{p}}\right) & , \quad i = \mathrm{su} \\ \upsilon_{\mathrm{tx,fl}}\left(\dfrac{\tau_{\mathrm{tx,fl,fa}}^{p}}{\tau_{\mathrm{tx,fl,su}}^{p} + \tau_{\mathrm{tx,fl,fa}}^{p}}\right) & , \quad i = \mathrm{fa} \\ \left(\dfrac{\upsilon_{\mathrm{tx,fl}}\left(\tau_{\mathrm{tx,fl,df}}^{p} + \tau_{\mathrm{tx,fl,de}}^{p}\right)}{\tau_{\mathrm{tx,fl,su}}^{p} + \tau_{\mathrm{tx,fl,fa}}^{p}}\right)\left(\dfrac{\tau_{\mathrm{tx,fl,df}}^{p}}{\tau_{\mathrm{tx,fl,df}}^{p} + \tau_{\mathrm{tx,fl,de}}^{p}}\right), & i = \mathrm{df} \\ \left(\dfrac{\upsilon_{\mathrm{tx,fl}}\left(\tau_{\mathrm{tx,fl,df}}^{p} + \tau_{\mathrm{tx,fl,de}}^{p}\right)}{\tau_{\mathrm{tx,fl,su}}^{p} + \tau_{\mathrm{tx,fl,fa}}^{p}}\right)\left(\dfrac{\tau_{\mathrm{tx,fl,de}}^{p}}{\tau_{\mathrm{tx,fl,df}}^{p} + \tau_{\mathrm{tx,fl,de}}^{p}}\right), & i = \mathrm{de} \end{cases} \tag{27}$$

$$\varepsilon_{\mathrm{tx,sl},i}^{p} = \begin{cases} \upsilon_{\mathrm{tx,sl}}\left(\dfrac{\tau_{\mathrm{tx,sl,su}}^{p}}{\tau_{\mathrm{tx,sl,su}}^{p} + \tau_{\mathrm{tx,sl,fa}}^{p}}\right) & , \quad i = \mathrm{su} \\ \upsilon_{\mathrm{tx,sl}}\left(\dfrac{\tau_{\mathrm{tx,fl,fa}}^{p}}{\tau_{\mathrm{tx,sl,su}}^{p} + \tau_{\mathrm{tx,sl,fa}}^{p}}\right) & , \quad i = \mathrm{fa} \\ \left(\dfrac{\upsilon_{\mathrm{tx,sl}}\left(\tau_{\mathrm{tx,sl,df}}^{p} + \tau_{\mathrm{tx,sl,de}}^{p}\right)}{\tau_{\mathrm{tx,sl,su}}^{p} + \tau_{\mathrm{tx,sl,fa}}^{p}}\right)\left(\dfrac{\tau_{\mathrm{tx,sl,df}}^{p}}{\tau_{\mathrm{tx,sl,df}}^{p} + \tau_{\mathrm{tx,sl,de}}^{p}}\right), & i = \mathrm{df} \\ \left(\dfrac{\upsilon_{\mathrm{tx,fl}}\left(\tau_{\mathrm{tx,sl,df}}^{p} + \tau_{\mathrm{tx,sl,de}}^{p}\right)}{\tau_{\mathrm{tx,sl,su}}^{p} + \tau_{\mathrm{tx,sl,fa}}^{p}}\right)\left(\dfrac{\tau_{\mathrm{tx,sl,de}}^{p}}{\tau_{\mathrm{tx,sl,df}}^{p} + \tau_{\mathrm{tx,sl,de}}^{p}}\right), & i = \mathrm{de} \end{cases} \tag{28}$$

$$\begin{aligned} \frac{dT_{\mathrm{fl}}^{a,h,s,p}(t)}{dt} &= \underbrace{\gamma_{\mathrm{tx}} g_{\mathrm{tx,fl}}^{p}\left(I_{s=\mathrm{ds}} + I_{s=\mathrm{dr}}(1 - g_{\mathrm{dst}})\right)D^{a,h,s,p}(t)}_{\substack{\text{first-line} \\ \text{treatment initation}}} - \underbrace{\left(\varepsilon_{\mathrm{tx,fl,su}}^{p} + \varepsilon_{\mathrm{tx,fl,fa}}^{p}\right)T_{\mathrm{fl}}^{a,h,s,p}(t)}_{\substack{\text{first-line} \\ \text{treatment completion}}} \\ &- \underbrace{\varepsilon_{\mathrm{tx,fl,df}}^{p} T_{\mathrm{fl}}^{a,h,s,p}(t)}_{\substack{\text{first-line} \\ \text{treatment default}}} - \underbrace{\varepsilon_{\mathrm{tx,fl,de}}^{p} T_{\mathrm{fl}}^{a,h,s,p}(t)}_{\substack{\text{first-line} \\ \text{treatment death}}} - \underbrace{I_{s=\mathrm{dr}}\eta T_{\mathrm{fl}}^{a,h,s,p}(t)}_{\substack{\text{self-} \\ \text{recovery}}} + \underbrace{\Delta Z_{\mathrm{adr}}^{a,h,s,p}(t)}_{\substack{\text{acquisition of} \\ \text{drug resistance}}} \\ &+ \underbrace{\Delta T_{\mathrm{fl}_{\mathrm{dmg}}}^{a,h,s,p}(t)}_{\substack{\text{demographic} \\ \text{change}}} + \underbrace{\Delta T_{\mathrm{fl}_{\mathrm{hiv}}}^{a,h,s,p}(t)}_{\substack{\text{HIV state} \\ \text{transition}}} \end{aligned} \tag{29}$$

The term $\Delta Z^{a,h,s,p}_{\text{adr}}(t)$ represents the acquisition of resistance to rifampicin during the course of first-line TB treatment. Once resistance is acquired, we assume that first-line treatment is no longer effective for infectious RR-TB cases.

$$\Delta Z^{a,h,s,p}_{\text{adr}}(t) = I_{s=\text{dr}}\sigma T^{a,h,\text{ds},p}_{\text{fl}}(t) - I_{s=\text{ds}}\sigma T^{a,h,\text{ds},p}_{\text{fl}}(t) \quad (30)$$

$$\begin{aligned}\frac{dT^{a,h,s,p}_{\text{sl}}(t)}{dt} = &\underbrace{I_{s=\text{dr}}\gamma_{\text{tx}} g^{p}_{\text{tx,sl}} g_{\text{dst}} D^{a,h,s,p}(t)}_{\substack{\text{second-line treatment}\\ \text{initiation}}} + \underbrace{I_{s=\text{dr}} g_{\text{tx,sw}} \varepsilon^{p}_{\text{tx,fl,fa}} T^{a,h,s,p}_{\text{fl}}(t)}_{\substack{\text{swtich from first- to}\\ \text{second-line treatment}}} \\ &- \underbrace{\left(\varepsilon^{p}_{\text{tx,sl,su}} + \varepsilon^{p}_{\text{tx,sl,fa}}\right) T^{a,h,s,p}_{\text{sl}}(t)}_{\substack{\text{second-line}\\ \text{treatment completion}}} - \underbrace{\varepsilon^{p}_{\text{tx,sl,df}} T^{a,h,s,p}_{\text{sl}}(t)}_{\substack{\text{second-line}\\ \text{treatment default}}} - \underbrace{\varepsilon^{p}_{\text{tx,sl,de}} T^{a,h,s,p}_{\text{sl}}(t)}_{\substack{\text{second-line}\\ \text{treatment death}}} \\ &+ \underbrace{\Delta T_{\text{sl}}{}^{a,h,s,p}_{\text{dmg}}(t)}_{\substack{\text{demographic}\\ \text{change}}} + \underbrace{\Delta T_{\text{sl}}{}^{a,h,s,p}_{\text{hiv}}(t)}_{\substack{\text{HIV state}\\ \text{transition}}}\end{aligned} \quad (31)$$

$$\begin{aligned}\frac{dE^{a,h,s}(t)}{dt} = &\underbrace{\gamma_{\text{tx}}\left(\left(1 - g^{p}_{\text{tx,fl}}\right)\left(I_{s=\text{ds}} + I_{s=\text{dr}}(1 - g_{\text{dst}})\right) + \left(1 - g^{p}_{\text{tx,sl}}\right) I_{s=\text{dr}} g_{\text{dst}}\right) D^{a,h,s,p}(t)}_{\substack{\text{lost-to-follow-up}\\ \text{prior to treatment initation}}} \\ &+ \underbrace{\left(I_{s=\text{ds}} + I_{s=\text{dr}}\left(1 - g_{\text{tx,sw}}\right)\right) \varepsilon^{p}_{\text{tx,fl,fa}} T^{a,h,s,p}_{\text{fl}}(t)}_{\substack{\text{first-line}\\ \text{treatment failure}}} + \underbrace{\varepsilon^{p}_{\text{tx,sl,fa}} T^{a,h,s,p}_{\text{sl}}(t)}_{\substack{\text{second-line}\\ \text{treatment failure}}} \\ &+ \underbrace{\varepsilon^{p}_{\text{tx,sl,df}} T^{a,h,s,p}_{\text{sl}}(t)}_{\substack{\text{second-line}\\ \text{treatment default}}} - \underbrace{c_{\text{2nd}} E^{a,h,s}(t)}_{\substack{\text{secondary}\\ \text{care-seeking}}} - \underbrace{\eta E^{a,h,s}(t)}_{\substack{\text{self-}\\ \text{recovery}}} + \underbrace{\Delta E^{a,h,s}_{\text{dmg}}(t)}_{\substack{\text{demographic}\\ \text{change}}} + \underbrace{\Delta E^{a,h,s}_{\text{hiv}}(t)}_{\substack{\text{HIV state}\\ \text{transition}}}\end{aligned} \quad (32)$$

For countries without the structure of public and private sectors, improvement of TB services is captured by a time-varying function that allows the proportion of treatment initiation to linearly increased over 2000-2010 in Supplementary Equation (33). The sector-specific proportions of treatment initiation, $g^{p}_{\text{tx,fl}}$ and $g^{p}_{\text{tx,sl}}$, are replaced with $g'_{\text{tx}}(t)$.

$$g'_{\text{tx}}(t) = \begin{cases} q_{\text{tx}} g_{\text{tx,max}} & , \ t < 2000 \\ \left(q_{\text{tx}} + \dfrac{(t - 2000)}{(2010 - 2000)}(1 - q_{\text{tx}})\right) g_{\text{tx,max}}, & 2000 \le t < 2010 \\ g_{\text{tx,max}} & , \ t \ge 2010 \end{cases} \quad (33)$$

where q_{tx} denotes the reduced level of treatment initiation prior to 2000, compared to the current capacity of a specific country.

- Recovered with a low, high, and stabilised risks of relapse ($R^{a,h}_{\text{lo}}$, $R^{a,h}_{\text{hi}}$, and $R^{a,h}_{\text{st}}$)

Recovered individuals may return to the active TB state again through relapse or reinfection. The relapse rate is higher among those who recovered naturally or default from the first-line treatment (φ_{hi}), compared to those with a successful outcome of anti-TB treatment (φ_{lo}). The difference of

relapse rate becomes very similar after two years from recovery (φ_{st}). On the other hand, reinfection of the recovered population is determined by the force of infection and the strength of protection from previous infection.

$$\frac{dR_{\mathrm{lo}}^{a,h}(t)}{dt} = \underbrace{\varepsilon_{\mathrm{tx,fl,su}}^{p} T_{\mathrm{fl}}^{a,h,s,p}(t) + \varepsilon_{\mathrm{tx,sl,su}}^{p} T_{\mathrm{sl}}^{a,h,s,p}(t)}_{\text{treatment success}} - \underbrace{\upsilon_{\mathrm{rec}} R_{\mathrm{lo}}^{a,h}(t)}_{\text{stabalisation}} - \underbrace{\varphi_{\mathrm{lo}} R_{\mathrm{lo}}^{a,h}(t)}_{\substack{\text{relapse} \\ \text{(low risk)}}} - \underbrace{\sum_{\tilde{s}} \lambda^{a,\tilde{s}}(t) q_{\mathrm{ltbi}} R_{\mathrm{lo}}^{a,h}(t)}_{\text{TB reinfection}} + \underbrace{\Delta R_{\mathrm{lo}}{}_{\mathrm{dmg}}^{a,h}(t)}_{\substack{\text{demographic} \\ \text{change}}} + \underbrace{\Delta R_{\mathrm{lo}}{}_{\mathrm{hiv}}^{a,h}(t)}_{\substack{\text{HIV state} \\ \text{transition}}} \tag{34}$$

$$\frac{dR_{\mathrm{hi}}^{a,h}(t)}{dt} = \underbrace{\eta \left(\sum_{s} \left(A^{a,h,s}(t) + E^{a,h,s}(t) \right) + \sum_{s,p} \left(D^{a,h,s,p}(t) + I_{s=\mathrm{dr}} T_{\mathrm{fl}}^{a,h,s,p} \right) \right)}_{\text{self-recovery}} + \underbrace{\varepsilon_{\mathrm{tx,fl,de}}^{p} T_{\mathrm{fl}}^{a,h,s,p}(t)}_{\substack{\text{first-line} \\ \text{treatment default}}} - \underbrace{\upsilon_{\mathrm{rec}} R_{\mathrm{hi}}^{a,h}(t)}_{\text{stabalisation}} - \underbrace{\varphi_{\mathrm{hi}} R_{\mathrm{hi}}^{a,h}(t)}_{\substack{\text{relapse} \\ \text{(high risk)}}} - \underbrace{\sum_{\tilde{s}} \lambda^{a,\tilde{s}}(t) q_{\mathrm{ltbi}} R_{\mathrm{hi}}^{a,h}(t)}_{\text{TB reinfection}} + \underbrace{\Delta R_{\mathrm{hi}}{}_{\mathrm{dmg}}^{a,h}(t)}_{\substack{\text{demographic} \\ \text{change}}} + \underbrace{\Delta R_{\mathrm{hi}}{}_{\mathrm{hiv}}^{a,h}(t)}_{\substack{\text{HIV state} \\ \text{transition}}} \tag{35}$$

$$\frac{dR_{\mathrm{st}}^{a,h}(t)}{dt} = \underbrace{\upsilon_{\mathrm{rec}} \left(R_{\mathrm{lo}}^{a,h}(t) + R_{\mathrm{hi}}^{a,h}(t) \right)}_{\text{stabalisation}} - \underbrace{\varphi_{\mathrm{st}} R_{\mathrm{st}}^{a,h}(t)}_{\substack{\text{relapse} \\ \text{(stable risk)}}} - \underbrace{\sum_{\tilde{s}} \lambda^{a,\tilde{s}}(t) q_{\mathrm{ltbi}} R_{\mathrm{st}}^{a,h}(t)}_{\text{TB reinfection}} + \underbrace{\Delta R_{\mathrm{st}}{}_{\mathrm{dmg}}^{a,h}(t)}_{\substack{\text{demographic} \\ \text{change}}} + \underbrace{\Delta R_{\mathrm{st}}{}_{\mathrm{hiv}}^{a,h}(t)}_{\substack{\text{HIV state} \\ \text{transition}}} \tag{36}$$

Supplementary Methods 2. Model calibration.

For each country model, we included calibration targets of demographics, TB and HIV burden, as listed in Supplementary Table 7. Treating these targets of multiple time points as independent data, we created a joint likelihood function, as:

$$
\begin{aligned}
L(\boldsymbol{\theta}) = {} & F_{\text{Pop}}^{(2018)}\left(M_{\text{Pop}}^{(2018)}(\boldsymbol{\theta})\right) + F_{\text{PrcCh}}^{(2018)}\left(M_{\text{PrcCh}}^{(2018)}(\boldsymbol{\theta})\right) + \sum_{t=2014}^{2017} F_{\text{Inc}}^{(t)}\left(M_{\text{Inc}}^{(t)}(\boldsymbol{\theta})\right) \\
& + F_{\text{IncCh}}^{(2018)}\left(M_{\text{IncCh}}^{(2018)}(\boldsymbol{\theta})\right) + F_{\text{IncAd}}^{(2018)}\left(M_{\text{IncAd}}^{(2018)}(\boldsymbol{\theta})\right) + \mathrm{F}_{\text{IncDR}}^{(2018)}\left(M_{\text{IncDR}}^{(2018)}(\boldsymbol{\theta})\right) \\
& + \sum_{t=2014}^{2018} F_{\text{Mort}}^{(t)}\left(M_{\text{Mort}}^{(t)}(\boldsymbol{\theta})\right) + I_{c=1} F_{\text{NtfPu}}^{(2018)}\left(M_{\text{NtuPu}}^{(2018)}(\boldsymbol{\theta})\right) \\
& + I_{c=2}\left[F_{\text{PrevHIV}}^{(2018)}\left(M_{\text{PrevHIV}}^{(2018)}(\boldsymbol{\theta})\right) + F_{\text{PrcART}}^{(2018)}\left(M_{\text{PrcART}}^{(2018)}(\boldsymbol{\theta})\right) \right. \\
& \left. + \sum_{t=2014}^{2018} F_{\text{IncHIV}}^{(t)}\left(M_{\text{IncHIV}}^{(t)}(\boldsymbol{\theta})\right)\right] + \sum UP(\boldsymbol{\theta}),
\end{aligned}
\tag{37}
$$

where $F_{target}^{(t)}$ represents the log-likelihood function of a calibration target at year t and $M_{target}^{(t)}(\theta)$ represents a model estimate of the target given parameters $\boldsymbol{\theta}$. According to the data types of calibration targets, we assumed log-normal distributions for rates and beta distributions for proportions. Confidence intervals provided by the original data sources, or the ± 20% range of point estimates when data were not available, were used to inform parameters that determine lognormal or beta distributions. In the function, we use I_c to indicate calibration targets that are included depending on model structures ($c = 1$ for public/private sectors and $c = 2$ for HIV epidemiology). The last term of the joint log-likelihood function, $\sum UP(\theta)$, denotes the overall log-probability density for uniform priors assumed in model calibration.

In a Bayesian framework, we calibrated each country model using the adaptive Monte-Carlo Markov chain approach (Supplementary Fig. 6), and then obtained 200 posterior samples. Supplementary Figs. 1-4. demonstrate model calibrations of demographics, and TB incidence and mortality rates. We also show model results of TB notification rate in public sectors (Supplementary Fig. 2), and HIV prevalence and ART coverage for countries with additional characteristics captured (Supplementary Fig. 5). Posterior distributions of key model parameters by country are summarised in Supplementary Table 1. To ensure these model structures are able to capture the country characteristics of TB transmission, we compared our model estimates to TB prevalence survey results in Supplementary Fig. 7, for countries with accessible data from 2010.

Supplementary Methods 3. Potential effects on antimicrobial resistance of future TB vaccines.

A TB vaccine could contribute to antimicrobial resistance control efforts. In particular, typical TB symptoms – particularly those that occur early in the clinical course of disease, including cough and fever – can be subtle and indistinguishable from non-TB aetiologies, such as other lower respiratory infections and chronic obstructive pulmonary disease (COPD). It is thus common for many TB cases to receive empirical, broad-spectrum antibiotics. Recent studies in India, Kenya and elsewhere, for example, highlighted the unnecessary prescriptions of quinolones and other antibiotics for empiric treatment [33]. In this secondary analysis we hypothesised that – by reducing TB burden – a future TB vaccine could reduce this antibiotic consumption by reducing the number of patients with respiratory symptoms presenting for primary care.

Because of a lack of systematic data from different countries on the antibiotics that are used for empiric therapy, we did not focus on specific antibiotics, and instead aimed to estimate the overall reduction in antibiotic consumption, that a future TB vaccine could achieve. For a given country setting, we aimed to estimate: (i) the cumulative number of episodes of respiratory illness, between 2020 and 2035, that are treated with antibiotics, and (ii) the reduction in this number as a result of the reductions in overall TB burden, reported in the main text.

We calculated this reduction using Supplementary Equations (38) and (39):

Proportion vaccine-induced reduction in overall antibiotic usage

= (TB cases averted by vaccination) / (All-cause incident cases of cough) (38)

Absolute vaccine-induced reduction in overall antibiotic usage

= (TB cases averted by vaccination) (39)

x (Proportion of cough that is medically attended and treated with antibiotics)

with all counts being cumulative between 2020 and 2035. An implicit assumption both of these formulae is that TB cases are equally likely as symptomatics without TB, to receive broad-spectrum antibiotic treatment.

In both equations, we drew estimates for the number of TB cases averted by vaccination from the modelling results described in the main text, using estimates for total (not just drug resistant) reductions in TB burden.

In Supplementary Equation (38) we drew the all-cause incident cases of cough using estimates from the Global Burden of Disease Study 2017, which quantifies global morbidity and mortality for a range of diseases and chronic conditions [30]. We aggregated the estimated numbers of incident cases of the following aetiologies: lower respiratory infections, asthma, COPD, all forms of TB, and other chronic respiratory diseases. Assuming this annual incidence to be stable over the 15 years of evaluation, we estimated the total incidence between 2020 and 2035. As described below, we also tested the sensitivity of our estimates, to different inclusion criteria for the denominator aetiologies.

In Supplementary Equation (39) we drew estimates for the proportion of cough that is treated with antibiotics, using data from the demographic health surveys (DHS), nationally representative

household surveys in over 90 countries, covering population, health and nutrition [28]. The data field most relevant to Supplementary Equation (39) is the proportion of acute respiratory illness that received antibiotic treatment, amongst children under 5 years of age. For simplicity, and in the absence of systematic data to do otherwise, we assumed that this proportion could be extrapolated to both chronic respiratory illness, and to older age groups. Available evidence from outpatient settings suggests that in the UK, antibiotic prescriptions for acute respiratory illness are higher amongst adults than children [21], and similarly for upper respiratory tract infections in Israel [34, 35]. If also true in the high-RR-TB-burden countries modelled in the present study, this would lead to an underestimation of antibiotic use and the resulting vaccine impact. DHS data is only available for 18 of the 30 countries in our modelling analysis: for consistency, we limited the estimates for both Supplementary Equations (38) and (39) to these countries.

Supplementary Fig. 13 shows the resulting estimates for projected, vaccine-induced reductions in antibiotic usage. These results suggest that, for the 18 countries presented here, a TB vaccine could avert 83,300 (95% CrI 67,700-98,000) courses of empiric antibiotic treatment between 2020 and 2035 (under the ‘status quo’ scenario presented in the main text), amounting to a population-weighted reduction of 0.29% (95% CrI 0.26%-0.31%) over this period.

Although substantial in absolute terms, these estimates suggest that TB vaccines are unlikely, by themselves, to have strong impact in relative terms, on antibiotic consumption beyond anti-TB drugs. The reason is that TB accounts for only a small proportion of respiratory symptomatics presenting for care. Even in a high burden setting like India, smear-positive TB cases accounted only for 12% of those who reported chronic symptoms of cough for more than two weeks [36]. This proportion is likely to be smaller when patients with shorter duration of cough symptoms are also included. As a sensitivity analysis we repeated our estimates for Supplementary Equation (38) when counting only asthma, COPD, and other respiratory symptoms for the denominator aetiologies (the categories with the lowest cumulative incidence and therefore yielding the highest estimates for the proportion). Under this approach the estimated effect of vaccination would be to reduce antibiotic usage by 2.11% (95% CrI 1.93%-2.30%), again demonstrating only a limited relative reduction. We also included upper respiratory infections in the denominator for the aetiologies for cough. Doing so reduces the vaccine impact on antibiotic usage substantially, to 0.011% (95% CrI 0.010%-0.012%).

Supplementary References

1. World Health Organization, *WHO's global tuberculosis database*.
2. UNAIDS, *AIDSinfo*, in *1990-2018*. 2020.
3. Wang, L., et al., *Tuberculosis prevalence in China, 1990-2010; a longitudinal analysis of national survey data.* Lancet, 2014. **383**(9934): p. 2057-64.
4. Qadeer, E., et al., *Population Based National Tuberculosis Prevalence Survey among Adults (>15 Years) in Pakistan, 2010-2011.* PLoS One, 2016. **11**(2): p. e0148293.
5. Indonesia Ministry of Health, *Indonesia tuberculosis prevalence survey 2013-2014*. 2015.
6. Nigeria Ministry of Health, *Report: First national TB prevalence survey 2012*.
7. Philippines Department of Health, *National Tuberculosis Prevalence Survey 2016 Philippines*. 2018: Manila, Philippines.
8. World Health Organization, *Global tuberculosis report 2019*. 2019: Geneva, Switzerland.
9. Nguyen, H.V., et al., *The second national tuberculosis prevalence survey in Vietnam.* PLoS One, 2020. **15**(4): p. e0232142.
10. Bangladesh Ministry of Health & Family Welfare, *National tuberculosis prevalence survey, Bangladesh 2015-2016*.
11. Enos, M., et al., *Kenya tuberculosis prevalence survey 2016: Challenges and opportunities of ending TB in Kenya.* PLoS One, 2018. **13**(12): p. e0209098.
12. Kebede, A.H., et al., *The first population-based national tuberculosis prevalence survey in Ethiopia, 2010-2011.* Int J Tuberc Lung Dis, 2014. **18**(6): p. 635-9.
13. Zimbabwe Ministry of Health & Child Care, *Report: The Zimbabwe national population based tuberculosis prevalence survey 2014*.
14. United Nations Deparatment of Economic and Social Affairs, *World Population Prospects*. 2019.
15. Croxford, S., et al., *Mortality and causes of death in people diagnosed with HIV in the era of highly active antiretroviral therapy compared with the general population: an analysis of a national observational cohort.* Lancet Public Health, 2017. **2**(1): p. e35-e46.
16. Rangaka, M.X., et al., *Isoniazid plus antiretroviral therapy to prevent tuberculosis: a randomised double-blind, placebo-controlled trial.* Lancet, 2014. **384**(9944): p. 682–90.
17. Suthar, A.B., et al., *Antiretroviral therapy for prevention of tuberculosis in adults with HIV: a systematic review and meta-analysis.* PLoS Med, 2012. **9**(7): p. e1001270.
18. Prem, K., A.R. Cook, and M. Jit, *Projecting social contact matrices in 152 countries using contact surveys and demographic data.* Plos Computational Biology, 2017. **13**(9).
19. Dodd, P.J., et al., *Burden of childhood tuberculosis in 22 high-burden countries: a mathematical modelling study.* Lancet Glob Health, 2014. **2**(8): p. e453-9.
20. Vynnycky, E. and P.E. Fine, *The natural history of tuberculosis: the implications of age-dependent risks of disease and the role of reinfection.* Epidemiol Infect, 1997. **119**(2): p. 183–201.
21. Dowdy, D.W. and R.E. Chaisson, *The persistence of tuberculosis in the age of DOTS: reassessing the effect of case detection.* Bull World Health Organ, 2009. **87**(4): p. 296-304.
22. Horsburgh, C.R., Jr., et al., *Revisiting rates of reactivation tuberculosis: a population-based approach.* Am J Respir Crit Care Med, 2010. **182**(3): p. 420–5.
23. Kaplan, R., et al., *HIV and TB co-infection in the ART era: CD4 count distributions and TB case fatality in Cape Town.* BMC Infect Dis, 2018. **18**(1): p. 356.

24. Tiemersma, E.W., et al., *Natural history of tuberculosis: duration and fatality of untreated pulmonary tuberculosis in HIV negative patients: a systematic review.* PLoS One, 2011. **6**(4): p. e17601.

25. Vesga, J.F., et al., *Assessing tuberculosis control priorities in high-burden settings: a modelling approach.* Lancet Glob Health, 2019. **7**(5): p. e585-e595.

26. Golub, J.E., et al., *Active case finding of tuberculosis: historical perspective and future prospects.* Int J Tuberc Lung Dis, 2005. **9**(11): p. 1183-203.

27. World Health Organization, *Public–private mix for TB prevention and care: a roadmap*. 2018: Switzerland.

28. ICF, *The DHS Program Data*. Rockville, US.

29. Watson, O.J., et al., *R: package 'rdhs'*. 2019. p. API Client and Dataset Management for the Demographic and Health Survey (DHS) Data.

30. Global Burden of Disease Collaborative Network, *Global Burden of Disease study 2017 (GBD 2017) results.* 2018, Institute for Health Metrics and Evaluation (IHME): Seattle, United States.

31. World Health Organization. *What is multidrug-resistant tuberculosis (MDR-TB) and how do we control it?* Online Q&A 2018 29 March 2020]; Available from: https://www.who.int/features/qa/79/en/.

32. Menzies, N.A., et al., *Progression from latent infection to active disease in dynamic tuberculosis transmission models: a systematic review of the validity of modelling assumptions.* Lancet Infect Dis, 2018. **18**(8): p. e228-e238.

33. Daniels, B., et al., *Lessons on the quality of tuberculosis diagnosis from standardized patients in China, India, Kenya, and South Africa.* J Clin Tuberc Other Mycobact Dis, 2019. **16**: p. 100109.

34. Low, M., et al., *Infectious disease burden and antibiotic prescribing in primary care in Israel.* Ann Clin Microbiol Antimicrob, 2018. **17**(1): p. 26.

35. Meropol, S.B., Z. Chen, and J.P. Metlay, *Reduced antibiotic prescribing for acute respiratory infections in adults and children.* Br J Gen Pract, 2009. **59**(567): p. e321-8.

36. Santha, T., et al., *Comparison of cough of 2 and 3 weeks to improve detection of smear-positive tuberculosis cases among out-patients in India.* Int J Tuberc Lung Dis, 2005. **9**(1): p. 61-8.
