# Supplementary material for: Modelling the global burden of drug-resistant tuberculosis avertable by a post-exposure vaccine
Source: Nat Commun. 2021 Jan 18;12:424. doi: 10.1038/s41467-020-20731-x (PMC7814030; doi:10.1038/s41467-020-20731-x)
Supplement: Supplementary file 2 — Reporting Summary [file 41467_2020_20731_MOESM2_ESM.pdf]

nature research

Corresponding author(s): Han Fu
Last updated by author(s): Dec 3, 2020

# Reporting Summary

Nature Research wishes to improve the reproducibility of the work that we publish. This form provides structure for consistency and transparency in reporting. For further information on Nature Research policies, see our Editorial Policies and the Editorial Policy Checklist.

## Statistics

For all statistical analyses, confirm that the following items are present in the figure legend, table legend, main text, or Methods section.

| n/a | Confirmed | |
|---|---|---|
| ☐ | ☒ | The exact sample size (*n*) for each experimental group/condition, given as a discrete number and unit of measurement |
| ☒ | ☐ | A statement on whether measurements were taken from distinct samples or whether the same sample was measured repeatedly |
| ☒ | ☐ | The statistical test(s) used AND whether they are one- or two-sided<br>*Only common tests should be described solely by name; describe more complex techniques in the Methods section.* |
| ☒ | ☐ | A description of all covariates tested |
| ☒ | ☐ | A description of any assumptions or corrections, such as tests of normality and adjustment for multiple comparisons |
| ☐ | ☒ | A full description of the statistical parameters including central tendency (e.g. means) or other basic estimates (e.g. regression coefficient) AND variation (e.g. standard deviation) or associated estimates of uncertainty (e.g. confidence intervals) |
| ☒ | ☐ | For null hypothesis testing, the test statistic (e.g. *F*, *t*, *r*) with confidence intervals, effect sizes, degrees of freedom and *P* value noted<br>*Give P values as exact values whenever suitable.* |
| ☐ | ☒ | For Bayesian analysis, information on the choice of priors and Markov chain Monte Carlo settings |
| ☒ | ☐ | For hierarchical and complex designs, identification of the appropriate level for tests and full reporting of outcomes |
| ☒ | ☐ | Estimates of effect sizes (e.g. Cohen's *d*, Pearson's *r*), indicating how they were calculated |

*Our web collection on statistics for biologists contains articles on many of the points above.*

## Software and code

Policy information about availability of computer code

| | |
|---|---|
| Data collection | Data were downloaded from publicly available sources, as described in the data availability statement. The DHS Program data were accessed using the package rdhs 0.6.3 in R 4.0.2. |
| Data analysis | The modelling analysis was conducted using MATLAB R2016b. Codes for estimating the vaccine impact on drug-resistant TB burden are available at https://github.com/hfu915/drtb_vacc. |

For manuscripts utilizing custom algorithms or software that are central to the research but not yet described in published literature, software must be made available to editors and reviewers. We strongly encourage code deposition in a community repository (e.g. GitHub). See the Nature Research guidelines for submitting code & software for further information.

## Data

Policy information about availability of data

All manuscripts must include a data availability statement. This statement should provide the following information, where applicable:

- Accession codes, unique identifiers, or web links for publicly available datasets
- A list of figures that have associated raw data
- A description of any restrictions on data availability

Country-specific data used in this study are publicly available and their sources are summarised in Supplementary Table 8, including: population statistics from the United Nations World Prospect Project 2019 (http://population.un.org/wpp/); TB incidence, mortality, and other programmatic indicators from the WHO Global Tuberculosis Database (http://www.who.int/tb/data/en/); HIV prevalence and coverage of antiretroviral treatment from the data tool of UNAIDS (http://aidsinfo.unaids.org/); and secondary school attendance from the United Nations Children's Fund (https://data.unicef.org/topic/education/secondary-education/). For analysis of antibiotic consumption for cough-related diseases, data were obtained from the DHS Program (http://dhsprogram.com/data/, through the rdhs 0.6.3 package in R 4.0.2) and the Global Burden of Disease Study 2017 (http://ghdx.healthdata.org/gbd-2017). Extracted data are also presented in Supplementary Table

7 or available at https://github.com/hfu915/drtb_vacc. Source data for Figs. 1-5 are provided with this paper.

# Field-specific reporting

Please select the one below that is the best fit for your research. If you are not sure, read the appropriate sections before making your selection.

☒ Life sciences ☐ Behavioural & social sciences ☐ Ecological, evolutionary & environmental sciences

For a reference copy of the document with all sections, see nature.com/documents/nr-reporting-summary-flat.pdf

# Life sciences study design

All studies must disclose on these points even when the disclosure is negative.

| | |
|---|---|
| Sample size | No samples were collected. Aggregated data used for the study were drawn from publicly available sources. |
| Data exclusions | No data of particular populations were excluded. |
| Replication | Data sources are publicly available. Codes used for the analysis in this study are also made publicly available. |
| Randomization | Randomization is not applicable to this study, because no primary data was collected, and this is a simulation-based study using only publicly available data. |
| Blinding | Blinding is not applicable to this study, because no primary data was collected, and this is a simulation-based study using only publicly available data. |

# Reporting for specific materials, systems and methods

We require information from authors about some types of materials, experimental systems and methods used in many studies. Here, indicate whether each material, system or method listed is relevant to your study. If you are not sure if a list item applies to your research, read the appropriate section before selecting a response.

## Materials & experimental systems

| n/a | Involved in the study | |
|---|---|---|
| ☒ | ☐ | Antibodies |
| ☒ | ☐ | Eukaryotic cell lines |
| ☒ | ☐ | Palaeontology and archaeology |
| ☒ | ☐ | Animals and other organisms |
| ☒ | ☐ | Human research participants |
| ☒ | ☐ | Clinical data |
| ☒ | ☐ | Dual use research of concern |

## Methods

| n/a | Involved in the study | |
|---|---|---|
| ☒ | ☐ | ChIP-seq |
| ☒ | ☐ | Flow cytometry |
| ☒ | ☐ | MRI-based neuroimaging |
